# Non-Classical Binding Mechanisms of Ferrocene-Modified Imatinib and Nilotinib Analogues in BCR-ABL1 Kinase Revealed by Computational Analysis

**DOI:** 10.3390/molecules31122156

**Published:** 2026-06-18

**Authors:** Rostislava Angelova, Georgi Stavrakov, Danislav S. Spassov, Georgi Momekov, Mariyana Atanasova

**Affiliations:** 1 Chemistry Department, Faculty of Pharmacy, Medical University of Sofia, 2 Dunav Str., 1000 Sofia, Bulgaria; 105645@students.mu-sofia.bg (R.A.); stavrakov@pharmfac.mu-sofia.bg (G.S.); dspassov@pharmfac.mu-sofia.bg (D.S.S.); 2 Department of Pharmacology, Pharmacotherapy and Toxicology, Faculty of Pharmacy, Medical University of Sofia, 2 Dunav Str., 1000 Sofia, Bulgaria; gmomekov@pharmfac.mu-sofia.bg; 3 Institute of Organic Chemistry with Centre of Phytochemistry, Bulgarian Academy of Sciences, Acad. G. Bonchev Str. 9, 1113 Sofia, Bulgaria; 4 Centre of Excellence in Informatics and Information and Communication Technologies, Bulgarian Academy of Sciences, Akad. Georgi Bonchev Str., Block 2 and 25A, 1113 Sofia, Bulgaria

**Keywords:** BCR-ABL1, chronic myeloid leukemia, ferrocene, molecular docking, molecular dynamics, MM-GBSA, tyrosine kinase inhibitors, structure-based drug design

## Abstract

Background: Ferrocene-containing compounds have gained attention in medicinal chemistry due to their unique redox and structural properties. This study investigates ferrocene-based analogues of imatinib and nilotinib to define their binding determinants within the ABL1 kinase domain using an integrated in silico approach, in relation to their previously reported cytotoxic activity. Methods: Ligand geometries were optimized at the B3LYP/def2-TZVP level with D3(BJ) dispersion and SMD solvation. Molecular docking against ABL1 (PDB ID: 2HYY) was performed using Glide SP, validated by re-docking and enrichment screening. Docked poses were refined using MM-GBSA (Prime, VSGB 2.1/OPLS4). The most active compounds (**9** and **15a**), together with the inactive control **15e**, were subjected to three independent 500 ns molecular dynamics simulations (Desmond, OPLS4), followed by trajectory analysis including RMSD, RMSF, radius of gyration, SASA, and polar surface area. Results: Compounds **9** and **15a** maintained stable binding within the ATP-binding pocket despite lacking the canonical hinge interaction with Met318, indicating hinge-independent binding. Their binding was mainly driven by interactions with Asp381 (DFG motif) and cation–π contacts with Lys271. In contrast, the compound **15e** showed unstable binding, increased conformational flexibility, reduced pocket burial, and loss of key stabilizing interactions. Active compounds also preserved stable P-loop dynamics, with Tyr253 engagement suggesting a role in loop stabilization. Compound **9** exhibited the most constrained and reproducible binding mode among all analogues. Conclusions: Ferrocene-based analogues can sustain stable ABL1 binding via non-classical interaction networks independent of hinge recognition. The clear distinction between active compounds and the inactive analogue **15e** supports the robustness of the proposed binding mode and provides a structural basis for their reported cytotoxic activity. These findings support further experimental evaluation of ferrocene-containing scaffolds as potential BCR-ABL1 inhibitors.

## 1. Introduction

Chronic myeloid leukemia (CML) is a malignant hematological disorder characterized by the uncontrolled proliferation of myeloid cells [1,2]. Its etiology is closely associated with the Philadelphia chromosome (Ph), arising from a reciprocal translocation between chromosomes 9 and 22 [t(9;22)(q34;q11)], first described by Nowell and Hungerford in 1960 [3,4,5]. This rearrangement leads to the formation of the BCR-ABL1 fusion gene, encoding an oncoprotein with constitutive tyrosine kinase activity. The BCR-ABL1 protein is expressed in hematopoietic progenitor cells of the myeloid lineage and, in some cases, in lymphoid progenitors [6], driving persistent activation of downstream signaling pathways, including STAT5, PI3K/AKT, and RAS/MAPK. These pathways promote uncontrolled proliferation, impaired apoptosis, and altered adhesion of leukemic cells to the bone marrow microenvironment [7,8,9,10,11,12,13,14].

The identification of the Philadelphia chromosome established the first direct link between a specific genetic alteration and malignant transformation, positioning CML as a paradigm for targeted anticancer therapy. The approval of imatinib by the U.S. Food and Drug Administration (FDA) in 2001 marked a major breakthrough in the development of tyrosine kinase inhibitors (TKIs) [15]. Despite their clinical success, resistance and suboptimal responses remain significant challenges [16]. Resistance frequently arises from point mutations within the kinase domain, with the gatekeeper mutation T315I being particularly problematic due to steric hindrance and disruption of key hydrogen-bonding interactions [17,18,19]. In addition, BCR-ABL1-independent resistance mechanisms have been reported [20,21]. Consequently, structural optimization of ATP-competitive inhibitors remains an active area of research aimed at improving binding affinity, overcoming resistance, and optimizing pharmacokinetic properties. One promising strategy involves incorporating organometallic fragments, particularly ferrocene, into biologically active scaffolds [22].

Ferrocene (Fc) is a versatile motif in medicinal chemistry. As a three-dimensional bioisostere of benzene and related heteroaromatic systems, it can enhance biological activity, modulate physicochemical properties, and reduce toxicity without substantially altering the parent scaffold [23,24,25]. Since its synthesis in 1951 and structural elucidation by Fischer and Wilkinson in 1952, Fc has attracted sustained interest due to its exceptional chemical stability and synthetic flexibility [26,27,28,29]. Notably, Fc exhibits relatively low intrinsic toxicity in preclinical models [30].

**Figure 1:**
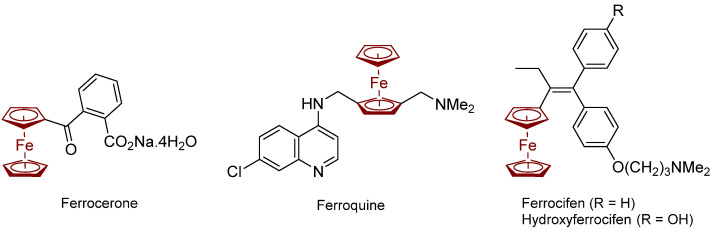
Chemical structures of Fc modified compounds, with the Fc moiety highlighted in dark red.

The anticancer potential of Fc derivatives has been recognized for several decades. Early studies by Fiorina et al. demonstrated that amino- and amide-substituted Fc derivatives possess antitumor activity in the P-388 leukemia model [31]. Since then, Fc-based compounds have been investigated across diverse therapeutic areas. Clinically relevant examples include ferrocerone, used for the treatment of iron-deficiency anemia in the former Soviet Union; ferroquine, evaluated in Phase IIb clinical trials as an antimalarial agent; and ferrocifens, currently under investigation for anticancer applications (Figure 1) [32,33,34,35].

In the context of BCR-ABL1 inhibition, incorporation of an Fc moiety may enhance hydrophobic complementarity within the ATP-binding site and modulate protein–ligand interaction networks. Structurally, Fc consists of an iron (II) center sandwiched between two parallel cyclopentadienyl (Cp) rings in an η^5^-coordination mode, with all carbon atoms positioned at approximately equal distances (~2.064 Å) from the metal center [36]. A distinctive feature of Fc is the nearly free rotation of the Cp rings about the metal axis, with a low rotational barrier (~3.8 kJ/mol), allowing rapid interconversion between eclipsed and staggered conformations in solution. This conformational flexibility, combined with the delocalized nature of Fe–C bonding, presents significant challenges for computational modeling [37,38], as classical biomolecular force fields do not fully capture such interactions.

These intrinsic structural and electronic characteristics highlight a broader methodological limitation, namely the difficulty of accurately describing transition-metal complexes in molecular simulations. In particular, organometallic systems such as Fc remain challenging to model with conventional approaches. Most conventional biomolecular force fields do not adequately reproduce the coordination chemistry and electronic structure associated with η^5^ bonding [39,40]. Alternative strategies, including the combination of general organic force fields with metal-specific parameterization schemes such as MCPB.py, enable the derivation of bonded parameters from quantum-mechanical calculations [41,42,43]. However, these approaches are primarily optimized for classical coordination geometries and are less suitable for sandwich-type complexes such as Fc.

Other methods, such as the GFN-FF (Geometries, Frequencies, and Non-covalent interactions Force Field), provide broader element coverage and are suitable for conformational sampling of metal-containing systems [44]. Nevertheless, their limited compatibility with standard biomolecular simulation frameworks restricts their applicability to large-scale protein–ligand simulations [40]. Modern force fields such as OPLS4 are highly effective for drug-like organic molecules and protein–ligand systems [45], but they have not been specifically parameterized for Fc-type coordination. Consequently, no universally validated classical force-field approach currently exists for routine simulations of Fc-containing ligands.

**Figure 2:**
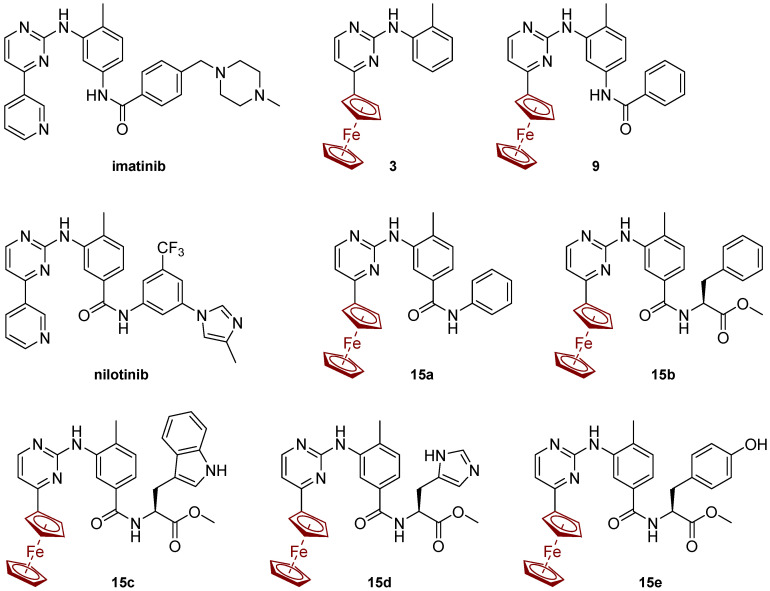
Chemical structures of BCR-ABL1 tyrosine kinase inhibitors, imatinib and nilotinib, and the designed Fc-containing analogs (**3**, **9**, **15a**–**e**) [46]. Fc moiety is highlighted in dark red.

In our previous work, we synthesized a series of seven Fc-containing analogs by replacing the pyridyl moiety of imatinib and nilotinib with a ferrocenyl group [46]. Biological evaluation against BCR-ABL1-positive leukemia cell lines (AR-230, BV-173, LAMA-84, K-562) identified compounds **9** and **15a** as the most potent derivatives (Figure 2). Notably, compound **15a** exhibited approximately 50-fold higher potency than imatinib in K-562 cells (IC_50_ = 0.8 μM vs. 45.5 μM), while compound **9** showed strong activity in the LAMA-84 model (IC_50_ = 0.9 μM). Both compounds demonstrated high selectivity for cancer cells over normal fibroblasts (CCL-1), with selectivity indices exceeding 200. In contrast, compounds **15b**–**e** displayed weaker activity, with IC_50_ values spanning the micromolar range (~10 to >200 μM) [46].

**Figure 3:**
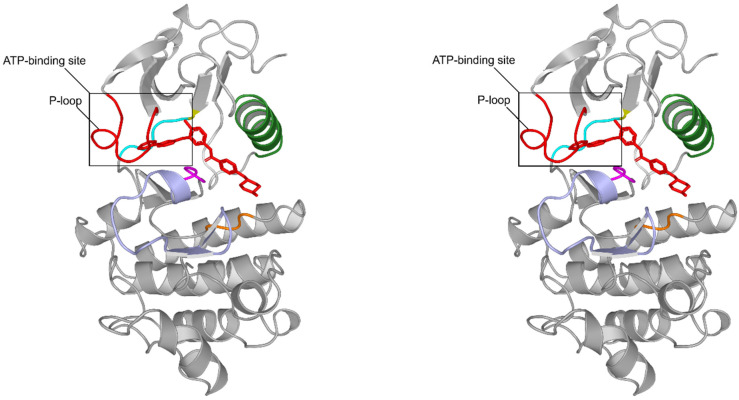
A wall-eye stereo pair presentation of the overall architecture of the BCR-ABL1 kinase domain used as the receptor model in this study. The Abl1 kinase domain (PDB ID: 2HYY, Chain B, [50]) is shown in cartoon representation with the N-lobe in light gray and the C-lobe in dark gray. Functionally important regions are highlighted as follows: P-loop (red), αC-helix (green), hinge region (cyan), HRD motif (orange), DFG motif (magenta), and activation loop (light blue). The gate-keeper residue Thr315 is highlighted in yellow. The crystallographic pose of imatinib is shown in red sticks.

As structural analogs of imatinib and nilotinib, these Fc-containing derivatives are expected to target the ATP-binding site of the ABL1 kinase domain. This domain exhibits the characteristic bilobal architecture of protein kinases, comprising N- and C-terminal lobes connected by a hinge region that forms the ATP-binding pocket (Figure 3). The N-lobe consists of five antiparallel β-sheets and a single α-helix (αC) that plays an important role in enzymatic activity. It also contains the ATP-binding loop (P-loop), a glycine-rich region located between the β1 and β2 sheets. The larger, predominantly α-helical C-terminal lobe contains the catalytic loop (C-loop) and the activation loop (A-loop), within which the DFG motif (Asp381–Phe382–Gly383) and the regulatory phosphorylation site Tyr393 reside [47], which is critical for inhibitor binding. In the active DFG-in conformation, the activation loop is extended, with Asp381 oriented toward the catalytic center, where it coordinates catalytic Mg^2+^ ions. In contrast, the DFG-out conformation induces collapse of the activation loop, exposing an allosteric pocket that is targeted by type II inhibitors such as imatinib [48]. The gatekeeper residue Thr315 plays a key role in modulating inhibitor access; its substitution with isoleucine (T315I) introduces steric hindrance and disrupts essential interactions, thereby conferring resistance to many inhibitors [17,49].

In this context, the present study employs an integrated in silico approach to investigate the binding behavior of novel Fc analogs of imatinib and nilotinib toward BCR-ABL1. By combining ab initio DFT calculations, molecular docking, molecular dynamics simulations, and MM-GBSA analyses, this work provides detailed atomistic insights into binding modes, interaction networks, and dynamic stability within the ABL1 kinase domain. To ensure consistency and enable direct comparison with previous studies, compound numbering has been retained from the original publication.

## 2. Results

### 2.1. Ab Initio Optimization of Studied Ligands

Due to the specific nature of the Fe–cyclopentadiene interactions, the Fc-substituted imatinib and nilotinib analogs were subjected to DFT optimization as described in Section 4. No imaginary frequencies were observed for any ligand, with the exception of compound **9**, which exhibited a single low-magnitude imaginary frequency (−3 cm^−1^). This was attributed to a numerical artifact and was therefore considered acceptable. The optimized ligand structures are presented in Figure S1 (Supplementary Materials). Comparison of the DFT-optimized Fc cores with the crystallographic reference extracted from the structure of avidin in complex with a ferrocene homobiotin derivative (PDB ID: 5MYQ [51]) demonstrated consistently high structural agreement across the studied compounds (Table S1, Supplementary Materials). The Fc moieties of compounds **3**, **9**, and **15a** showed RMSD values of approximately 0.02 Å, indicating near-perfect reproduction of the reference metallocene geometry. In contrast, derivatives **15b**–**e** exhibited moderately higher deviations (RMSD: 0.41–0.42 Å). Although still within an acceptable range, these differences suggest that substituent patterns subtly influence Fc core geometry. In particular, bulkier amino acid–derived fragments in peptidomimetic designs introduce steric and conformational constraints, leading to measurable distortions, whereas less substituted compounds retain geometries closer to the X-ray-derived structure.

### 2.2. Drug-Likeness Evaluation

The compounds studied were evaluated for drug-likeness according to Lipinski’s Rule of Five (Ro5), which also serves as a guideline for oral bioavailability (Table 1). The molecular weights of compounds **3**, **9**, and **15a** remained below 500 Da, while compounds **15b**–**e** exceeded this threshold. The hydrogen-bond donor (HBD) and hydrogen-bond acceptor (HBA) counts fell within ranges generally considered compatible with drug-like molecules. The calculated logP values ranged from 4.13 to 6.30, indicating moderate to high lipophilicity across the investigated ferrocene-containing derivatives. Compounds **9** and **15a** exhibited the highest logP values (6.30 and 6.20, respectively), whereas compounds **15d** and **15e** showed lower values (4.13 and 4.54, respectively).

The calculated physicochemical descriptors indicate that the investigated compounds retain an overall drug-like profile despite the incorporation of the ferrocene moiety. While compounds **9** and **15a** displayed increased lipophilicity, compounds **15d** and **15e** showed lower logP values, suggesting that the introduction of additional polar peptidomimetic structural elements partially counterbalances the hydrophobic contribution of the ferrocene and aromatic fragments.

According to Lipinski’s Rule of Five, most of the investigated compounds exhibited one or two violations, primarily related to molecular weight and/or lipophilicity, which is commonly observed among kinase inhibitors and other targeted anticancer agents.

### 2.3. Validation of the Docking Protocol 

To ensure reliability, predictive performance, and the ability to distinguish active inhibitors from decoys, the docking protocol was validated in two steps. First, the crystallographic ligand imatinib in complex with c-Abl1 (PDB: 2HYY [50]) was redocked as described in Section 4. This was followed by retrospective virtual screening of known BCR-ABL1 actives and decoys.

**Figure 4:**
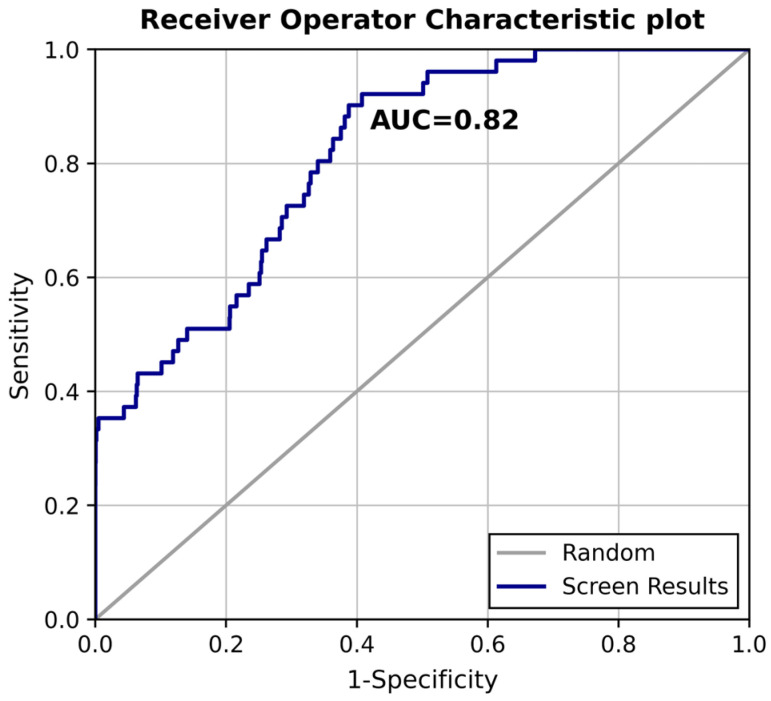
Receiver Operating Characteristic (ROC) curve for the validation of the Glide SP docking protocol against the BCR-ABL1 kinase (PDB: 2HYY) [50], generated using the enrichment analysis module in Schrödinger Release 2025-4 (Glide). The curve is based on a dataset comprising 51 active compounds and 5542 decoys derived from the DUD-E database and DeepCoy-generated decoys [52,53].

The inner grid box size was evaluated at 10, 12, and 14 Å. The 12 Å grid produced the lowest RMSD (0.397 Å) between the redocked and crystallographic poses and was therefore selected for subsequent calculations. The ROC curve from the second validation step yielded an AUC of 0.82 (Figure 4), indicating good discriminatory power. Recovery of active compounds at different thresholds is summarized in Table 2.

Boltzmann-enhanced discrimination of ROC (BEDROC) values of 0.406 (α = 20) and 0.502 (α = 160.9) demonstrated strong early enrichment, with active compounds preferentially ranked at the top of the list. At 1% of the ranked database, 35.3% of active compounds were recovered (sensitivity = 0.35), with a specificity of 0.99 and an enrichment factor of 35.0.

### 2.4. Molecular Docking Analyses

Application of the validated docking protocol to Fc-containing imatinib and nilotinib analogs, combined with pose selection criteria—where the top-ranked pose for each ligand was chosen based on docking score, absence of steric clashes, and consistency within the ATP-binding site—yielded GlideScores ranging from −4.60 to −9.27 kcal/mol (Table 3). In comparison, imatinib exhibited a more favorable score of −12.62 kcal/mol, while nilotinib achieved the most favorable GlideScore among all docked compounds (−13.85 kcal/mol), consistent with its established high-affinity binding to BCR-ABL1 [46,54].

The relatively lower GlideScore values observed for the Fc derivatives compared to imatinib may partly reflect the limited ability of conventional docking scoring functions to describe η^5^ Fe–Cp organometallic bonding and the associated dispersion and electrostatic contributions of the ferrocene fragment. Consequently, fewer interactions may be accounted for, leading to lower overall scores, as the final scoring function represents the sum of intermolecular interaction terms.

**Figure 5:**
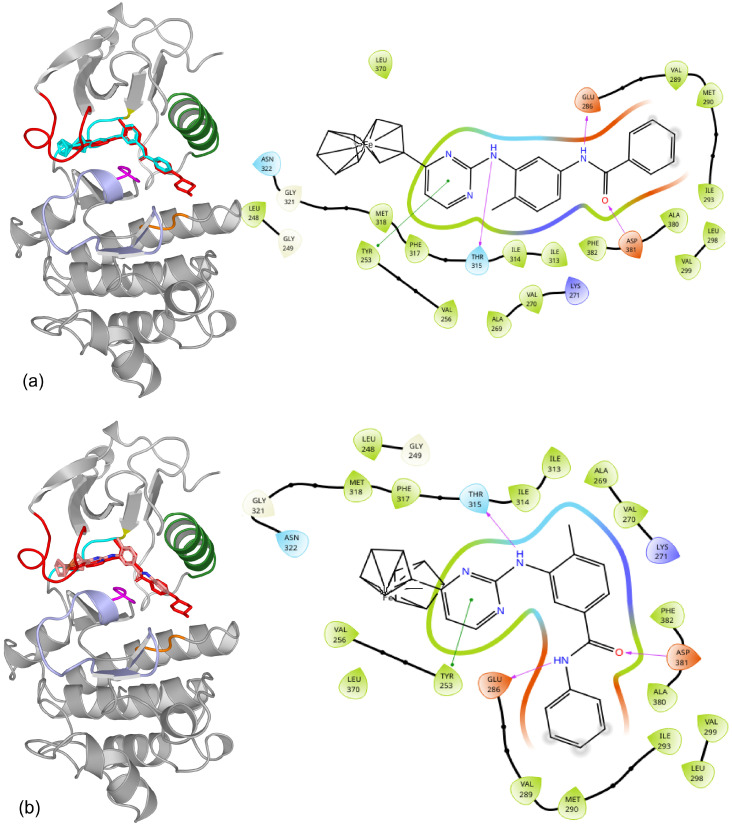
Comparative binding modes (**left panels**) of imatinib (red; crystallographic pose from PDB ID: 2HYY [50] and selected Fc-containing analogues—(**a**) **9** (cyan) and (**b**) **15a** (pink)—within the ATP-binding pocket of the BCR-ABL1 kinase, together with the corresponding ligand–protein interaction patterns (**right panels**). In the right panels, residues are color-coded according to physicochemical properties (hydrophobic, polar, acidic, basic, or glycine). Interaction symbols denote the nature of ligand–protein contacts: magenta arrows indicate hydrogen bonds (from donor to acceptor), green lines represent π–π stacking interactions, and gray spheres indicate solvent exposure or weak van der Waals contacts.

Compounds **9** and **15a** adopted binding modes comparable to imatinib (Figure 5), while lower-ranked compounds **3** and **15b**–**e** frequently exhibited shifted orientations, often accompanied by partial solvent exposure or displacement of the metallocene moiety from the hydrophobic pocket (Figure S2; Supplementary Materials). Interaction fingerprint analysis of representative docking poses revealed distinct interaction profiles between active compounds in the series, including imatinib, and inactive ones (Figure S3, Supplementary Materials). Compounds **9** and **15a** reproduced the core interaction pattern characteristic of ATP-competitive BCR-ABL1 inhibitors. The Fc moiety occupied the hydrophobic pocket formed by the P-loop and hinge region, analogous to the pyridine ring of imatinib, extending into the same region of the binding site. Key stabilizing hydrogen-bonding interactions were preserved, involving the anilino NH and the amide linker NH and carbonyl groups with Thr315 (gatekeeper), Glu286 (αC), and Asp381 (DFG), respectively. In addition, a π–π stacking interaction between the pyrimidine ring and Tyr253 (P-loop) was maintained. Overall, this interaction profile closely mirrors that of the corresponding imatinib scaffold.

In contrast, compounds **15b**–**d** exhibited disrupted interaction networks and peripheral binding. Compound **3** showed partial conservation of interactions, while **15e** adopted an intermediate binding mode lacking full hinge and aromatic network formation. These incomplete interaction profiles are consistent with reduced in vitro cytotoxicity.

### 2.5. MM-GBSA Analysis of Docking Poses

MM-GBSA calculations were performed to compare selected docking poses within a consistent computational workflow (Table 4). The ferrocene moiety was explicitly included in all ligand structures throughout docking, molecular dynamics simulations, and MM-GBSA calculations. Within the OPLS4 force-field framework, the iron center carries an assigned partial charge, while Fe–C interactions with the cyclopentadienyl rings are represented as zero-order bonds. The internal geometry of the ferrocene unit is maintained close to the DFT-optimized reference structure via distance restraints. Consequently, the ferrocene fragment is treated as an effectively rigid body within the molecular mechanics description, rather than through an explicit bonded model of the η^5^ Fe–Cp interaction.

The resulting ΔG_bind_ values were used exclusively as relative, protocol-consistent estimates for ranking ligand poses and were not interpreted as absolute experimental binding free energies. In this context, imatinib and nilotinib yielded highly favorable values (−97.15 and −99.67 kcal/mol, respectively), whereas the ferrocene derivatives showed comparatively weaker binding estimates, with compounds **9** and **15a** exhibiting the most favorable values within this series (−22.92 and −14.07 kcal/mol, respectively).

Although ferrocene is explicitly represented within the OPLS4 force field, standard MM-GBSA methodologies were not originally parameterized for organometallic systems; therefore, the computed energies should be regarded as semi-quantitative and system-dependent rather than directly comparable to experimental binding free energies. Accordingly, these values were not used as the primary basis for mechanistic interpretation.

The robustness of the ferrocene representation is further supported by molecular dynamics simulations, which confirm preservation of the η^5^-sandwich geometry throughout the trajectories (Section 2.6.1).

**Figure 6:**
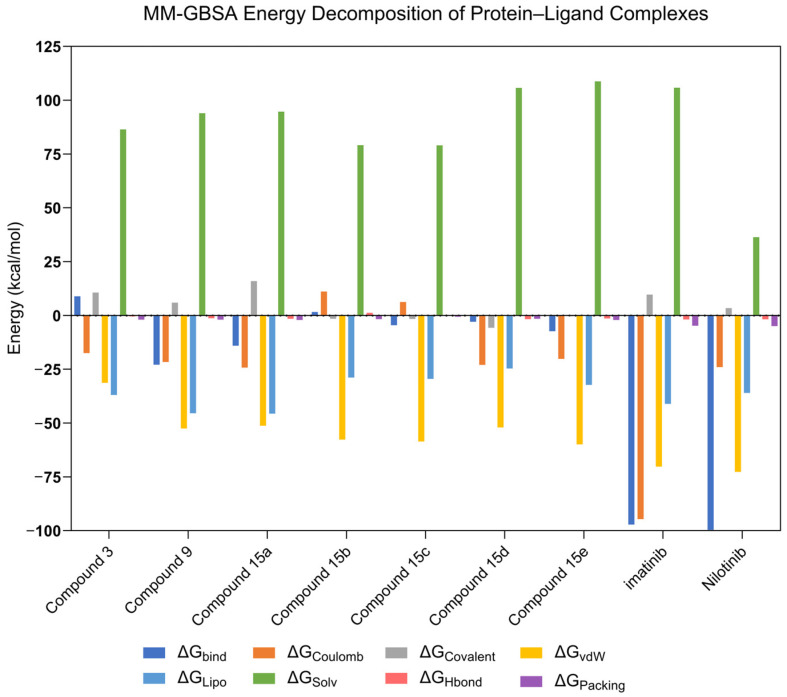
MM-GBSA energy decomposition of protein–ligand complexes for compounds **3**, **9**, **15a**–**e**, nilotinib, and imatinib.

Energy decomposition analysis (Figure 6 and Table S2, Supplementary Materials) indicated that, among the interaction terms captured by the MM-GBSA model, van der Waals and lipophilic interactions were the main favorable contributors. Electrostatic contributions were modest, while hydrogen-bonding contributions were comparatively minor. This trend is consistent with the predominantly hydrophobic nature of the ATP-binding pocket. The solvation term partially counterbalanced the favorable interaction energies and, in some lower-ranked analogues, fully offset them, resulting in less favorable overall MM-GBSA ΔG_bind_ values.

### 2.6. Molecular Dynamics

#### 2.6.1. Protein and Ligand Stability

Backbone RMSD analysis confirmed the structural stability of ABL1 across all simulations (1.2–2.6 Å) (Figure S4, Supplementary Materials). Comparable RMSD profiles indicate that ligand binding does not significantly perturb the kinase architecture. Compound **9** showed a slightly lower RMSD (~1.8 Å), suggesting a more stable complex, while nilotinib (~2.4 Å) and compound **15e** (~2.3 Å) remained within the same stable range.

Ligand RMSD analysis revealed stable binding geometries for compounds **15a** (RMSD_average_~1.9 Å) and **9** (RMSD_average_~1.65 Å) (Figure S5, Supplementary Materials). Notably, imatinib exhibited greater positional variability (RMSD_average_~2.1 Å), suggesting broader conformational sampling within the binding site, whereas nilotinib remained comparatively well anchored (RMSD_average_ ~0.7 Å). In contrast, the inactive control compound **15e** displayed the highest ligand mobility among the ferrocene-containing analogues (RMSD_average_ ~2.1 Å).

Additionally, trajectory analysis focusing on P-loop backbone RMSD was performed to explore a potential mechanism of inhibition recently proposed by our group, namely the inhibitor-trapping mechanism [55,56,57,58]. The average RMSD values for each trajectory are presented in Table 5 and Table S3.

P-loop analysis indicated generally stable behavior across the active and reference systems (Figures S6 and S7, Supplementary Materials). In the complex with compound **9**, the P-loop exhibited the lowest variability, whereas in the complex with compound **15a** it showed slightly increased flexibility, although without significant conformational disruption. Importantly, these fluctuations remained limited and were not accompanied by major P-loop opening or persistent displacement, suggesting that the loop maintained an overall stable, closed conformation in the active and reference complexes. In contrast, the weakly active control compound **15e** showed markedly higher and more variable P-loop RMSD, most pronounced in replica 1, consistent with reduced stabilization of the P-loop relative to the active compounds.

The per-residue Cα root-mean-square fluctuation (RMSF) of the P-loop (residues 247–255) was highest for compound **15e** (1.89 ± 0.48 Å) nd lowest for imatinib (1.26 ± 0.06 Å), while compound **9** (1.31 ± 0.17 Å), nilotinib (1.32 ± 0.15 Å), and **15a** (1.52 ± 0.10 Å) exhibited intermediate values (Table 6). The RMSF profiles also differed in their distribution. In the active and reference complexes, the maximum fluctuation was centered at the glycine-rich apex (Gly250), whereas for **15e** the peak shifted to Gln252, adjacent to Tyr253, reaching 3.85 Å in the most mobile replica. The overall profiles remained highly consistent between the two superposition protocols, with mean differences not exceeding 0.1 Å (Figures S8 and S9; Table S4, Supplementary Materials).

RMSF analysis confirmed similar overall flexibility profiles across systems (Figure S10, Supplementary Materials). Terminal residues showed elevated fluctuations, as expected, and were excluded from mechanistic interpretation. The P-loop region (residues ~247–257) displayed moderate fluctuations, with the imatinib complex showing the lowest mean RMSF (~1.17 Å), indicating loop stabilization through direct ligand contact. Compound **9** exhibited a comparable mean RMSF (~1.21 Å), suggesting a similar degree of P-loop restraint, whereas compound **15a** showed slightly higher values (~1.42 Å), consistent with reduced rigidity in this region. A similar level was observed for nilotinib (~1.50 Å), closely matching its ferrocene analogue **15a**, while the inactive compound **15e** displayed the highest P-loop mean RMSF (~1.72 Å), consistent with the weakest loop restraint among all systems. The αC-helix region (residues ~279–292) also remained stable across the active and reference complexes (~1.11–1.38 Å), with no significant differences between ligands, whereas compound **15e** showed modestly elevated fluctuations (mean ~1.79 Å).

Differences between complexes were observed in the hinge region (residues 317–322) and the activation loop (residues ~380–401). The imatinib complex showed the lowest hinge fluctuations (~0.53–0.63 Å), consistent with the persistent Met318 backbone hydrogen bond identified in the interaction fingerprint analysis (Section 2.6.7). In contrast, compounds **9** and **15a** exhibited greater hinge flexibility (~0.57–0.91 Å and ~0.78–1.19 Å, respectively), with compound **15a** showing higher inter-replica variability. This behavior is consistent with the absence of a persistent canonical Met318 hinge hydrogen bond in the ferrocene-containing complexes. Nilotinib and compound **15e** showed intermediate hinge fluctuations (~0.65–0.89 Å and ~0.70–1.05 Å, respectively).

The imatinib complex also displayed pronounced activation-loop flexibility (peak ~4.78 Å at residue 397, SD ± 1.1 Å), consistent with stabilization of the DFG-out inactive conformation while retaining residual loop mobility. Compound **15a** exhibited similarly high fluctuations, peaking at ~4.26 Å (SD ± 0.7 Å), indicating substantial conformational sampling across replicas. In contrast, compound **9** showed lower activation-loop fluctuations (peak ~2.75 Å, SD ± 0.71 Å), suggesting more effective and reproducible stabilization of this region, likely due to deeper hydrophobic burial of the ferrocene moiety and a high-occupancy cation–π interaction with Lys271. Nilotinib displayed pronounced activation-loop flexibility (peak ~5.86 Å at residue 397, SD ± 2.1 Å), consistent with its role in stabilizing the DFG-out inactive conformation while allowing residual loop mobility, whereas compound **15e** showed intermediate but highly reproducible activation-loop fluctuations (peak ~3.76 Å at residue 397, SD ± 0.14 Å).

The integrity of the ferrocene core throughout the 500 ns simulations was assessed using geometric and conformational descriptors sensitive to the preservation of the η^5^-ferrocene sandwich architecture, including Fe–C bond distances, Fe–Cp centroid distances, the Cp–Cp centroid distance, the interplanar angle between the cyclopentadienyl rings, ring planarity, and the rotational offset between the Cp rings. The ferrocene core remained structurally stable throughout the simulations. The overall ferrocene-core RMSD relative to the corresponding DFT reference structures remained low for both active analogues, with values of 0.107 ± 0.019 Å for compound **9** and 0.115 ± 0.019 Å for compound **15a**. For compound **9**, the mean Fe–C distance was 2.111 ± 0.009 Å, while the mean Fe–Cp centroid distances were 1.677 ± 0.017 Å and 1.672 ± 0.017 Å for the two Cp rings, respectively. The Cp–Cp centroid distance also remained close to the DFT reference value (3.348 ± 0.023 Å versus 3.353 Å), and the mean interplanar angle between the Cp rings was 2.60 ± 1.31°, confirming preservation of the characteristic near-parallel ferrocene sandwich geometry. Similar behavior was observed for compound **15a**, which showed a mean Fe–C distance of 2.108 ± 0.009 Å and a Cp–Cp centroid distance of 3.341 ± 0.023 Å. Comparison with an independent crystallographic ferrocene reference fragment extracted from the structure PDB ID 5MYQ further confirmed the absence of progressive distortion of the ferrocene sandwich architecture (Table S5, Supplementary Materials).

The inactive control compound **15e** displayed equivalent ferrocene-core stability. Its ferrocene-core RMSD relative to the DFT reference was 0.098 ± 0.012 Å, with a mean Fe–C distance of 2.107 ± 0.009 Å, a Cp–Cp centroid distance of 3.332 ± 0.023 Å versus a DFT reference value of 3.351 Å, and a mean Cp–Cp interplanar angle of 2.69 ± 1.36°. These values closely matched those obtained for the active analogues, indicating that the increased ligand RMSD of compound **15e** cannot be attributed to distortion of the ferrocene core. Complete validation data are provided in Supplementary Table S5.

The observed geometric deviations remained small throughout the 500 ns simulations and within ranges consistent with preservation of the characteristic ferrocene structure. Collectively, these analyses demonstrate that the OPLS4 representation preserved the key geometric and conformational features of the ferrocene scaffold throughout the simulations and did not exhibit structural distortions that would indicate force-field instability or breakdown of the η^5^-sandwich architecture.

To assess whether the differences observed in the binding site were reproduced across independent simulations, the trajectories were analyzed using principal component analysis (PCA), cosine content analysis, and free-energy landscape (FEL) reconstruction. For compounds **9** and **15a**, the first two system-specific principal components accounted for 26.7% and 53.4% of the structured-core variance, respectively. The same analysis was performed for imatinib, nilotinib, and the negative control analogue **15e**, for which the first two components accounted for 28.0%, 31.5%, and 51.1% of the variance, respectively. The differences in variance distribution indicate that compound exhibits more anisotropic conformational fluctuations, with motion preferentially distributed along a smaller number of dominant collective modes.

For the two active ferrocene-containing compounds, mean cosine content values remained low. The PC1/PC2 values were 0.14 ± 0.12/0.04 ± 0.06 for compound **9** and 0.10 ± 0.16/0.09 ± 0.15 for compound **15a**. These values suggest an absence of dominant random-walk-like diffusion along the principal components, indicating structured sampling within metastable regions.

The FEL maps further supported differences between the two active ferrocene-containing compounds (Figure S11). Compound **9** occupied a relatively compact low-energy region, consistent with more restricted conformational sampling of the structured kinase core across replicas. Compound **15a**, in contrast, populated multiple broader and partially connected low-energy regions, indicating increased conformational heterogeneity. Accordingly, compound **15a** was not interpreted as a single narrowly defined binding-site state; instead, the mechanistic interpretation focused on interaction patterns and binding-site features consistently observed across all three replicas The reference inhibitors also formed well-defined low-energy regions in their respective FELs. In contrast, the inactive analogue **15e** exhibited a more fragmented landscape with multiple disconnected low-energy basins, consistent with increased conformational instability and the sustained P-loop opening described above. Because separate PCA models were fitted for each system, absolute PC1/PC2 coordinates were not directly comparable between systems; therefore, FEL minima positions are reported only as system-specific descriptors. The corresponding PCA and FEL plots are provided in Supplementary Figures S11 and S12, and quantitative parameters are summarized in Table S6.

#### 2.6.2. Cluster Analysis

RMSD-based clustering of the ligand and surrounding binding-site protein atoms revealed distinct differences in conformational ensemble compactness among the simulated systems.

Imatinib exhibited moderate replica-to-replica variability. In the second simulation, a single dominant cluster accounted for 80.7% of the trajectory (~403.5 ns), with a medoid at 235.8 ns, indicating a relatively stable conformational state. In contrast, the first and third simulations showed broader distributions. The first replica was partitioned between two major clusters, accounting for 35.7% and 32.1% of the trajectory (178.3 ns and 160.7 ns, respectively), while the third replica displayed an even broader distribution across three similarly populated clusters (27.9%, 27.1%, and 25.0%, corresponding to 139.7 ns, 135.7 ns, and 125.1 ns, respectively) (Figure S13, Supplementary Materials).

**Figure 7:**
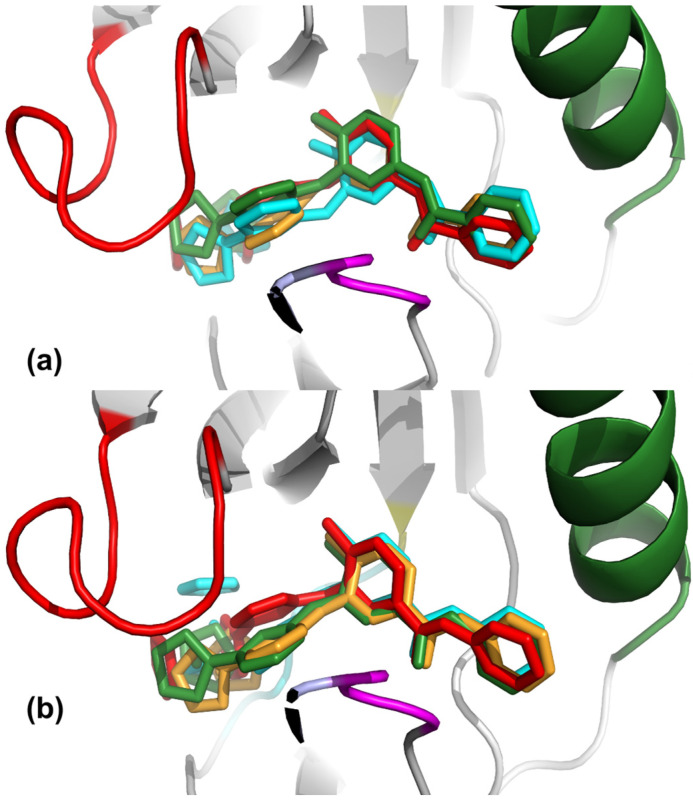
Superposition of representative structures from the most populated MD clusters within the ATP-binding site of BCR-ABL1 (PDB: 2HYY [50]). For each compound, representative frames from replica 1 (cyan), replica 2 (green), and replica 3 (bright orange) are overlaid with the initial docking pose (frame 0, red). Protein secondary structure elements are color-coded by region: P-loop (red), αC-helix (green), hinge region (cyan), DFG motif (magenta), and activation loop (light blue). (**a**) Compound **9**: dominant cluster representatives from replica 1 (304.8 ns), replica 2 (289.8 ns), and replica 3 (258.8 ns); (**b**) Compound **15a**: dominant cluster representatives from replica 1 (388.6 ns), replica 2 (351.0 ns), and replica 3 (337.4 ns).

Compound **9** showed a stronger tendency toward conformational convergence (Figure 7). The first replica converged to a single dominant conformation, with the most populated cluster accounting for 91.1% of the trajectory (455.6 ns) and a medoid at 304.8 ns. The second and third replicas exhibited greater heterogeneity. In the third replica, a dominant cluster occupied 36.2% of the trajectory (180.9 ns; medoid at 258.8 ns), followed by two secondary clusters accounting for 27.4% and 16.1% (137.1 ns and 80.7 ns, respectively). Replica 2 remained more distributed, with three major clusters accounting for 21.0%, 20.1%, and 18.5% of the frames and medoids at 289.8 ns, 139.2 ns, and 415.2 ns, respectively.

Compound **15a** exhibited the most heterogeneous clustering pattern among the active compounds (Figure 7). The first replica was primarily divided between two major clusters accounting for 35.4% and 30.8% of the trajectory (177.1 ns and 154.1 ns, respectively). The third replica was dominated by a single cluster occupying 42.8% of the trajectory (213.8 ns; medoid at 337.4 ns), followed by two secondary clusters accounting for 19.7% and 13.7% (98.3 ns and 68.3 ns, respectively). The second replica showed extensive fragmentation, with the largest cluster accounting for only 15.9% of the trajectory and a total of 32 clusters identified, indicating broad sampling of multiple local conformational states.

Nilotinib displayed a comparatively well-defined conformational ensemble (Figure S13, Supplementary Materials), generating only five to nine clusters per replica. In the first replica, a single dominant cluster accounted for 50.0% of the trajectory (250.2 ns; medoid at 429.7 ns), followed by two secondary clusters accounting for 19.5% and 11.2% (97.7 ns and 56.2 ns, respectively). The second replica showed a similar pattern, with the most populated cluster accounting for 34.4% of the trajectory (172.1 ns; medoid at 464.5 ns) and two additional clusters accounting for 22.9% and 18.9% (114.7 ns and 94.5 ns, respectively). In the third replica, two nearly equally populated clusters dominated the ensemble, occupying 49.4% (247.0 ns; medoid at 423.3 ns) and 44.6% (222.8 ns) of the trajectory, respectively, while a minor third cluster accounted for only 3.0% (15.0 ns). Across all three replicas, a single dominant—or, in the third replica, bistable—binding mode was consistently recovered, indicating relatively stable engagement within the binding pocket.

In contrast, compound **15e** exhibited the most fragmented clustering pattern of all systems (Figure S13, Supplementary Materials), with the number of distinct clusters ranging from 18 to 33 per replica. In the second replica, the largest cluster accounted for only 37.2% of the trajectory (185.9 ns; medoid at 34.8 ns), despite a total of 33 clusters being identified. The first replica was even more dispersed, with the most populated cluster accounting for only 26.6% of the trajectory (133.1 ns; medoid at 257.2 ns) among 30 clusters. The third replica showed extensive fragmentation, with the three largest clusters being nearly equally populated (16.3%, 16.1%, and 15.5%, corresponding to 81.7 ns, 80.7 ns, and 77.3 ns, respectively). This pronounced conformational dispersion is consistent with the elevated ligand RMSD and reduced P-loop stabilization observed for compound **15e**, reflecting the absence of a persistent and well-defined binding mode for the inactive control analogue.

#### 2.6.3. Radius of Gyration (rGyr)

The radius of gyration (Rg) provides a measure of molecular compactness and structural integrity throughout the simulation and is defined as the root mean square distance of all atoms from the molecular center of mass. The Rg values remained stable for all compounds during the production phase (Figure S14, Supplementary Materials).

Both ferrocene analogues exhibited greater compactness than their respective parent drugs: compound **9** displayed an average Rg of 5.76 ± 0.04 Å compared with 7.33 ± 0.08 Å for imatinib, while compound **15a** showed an average Rg of 5.79 ± 0.04 Å compared with 6.79 ± 0.06 Å for nilotinib. This difference reflects the intrinsically more compact and globular geometry of the ferrocene scaffold relative to the more elongated architecture of the parent inhibitors.

The inactive control compound **15e** exhibited a comparable mean Rg value (5.80 ± 0.46 Å); however, its substantially larger standard deviation indicates greater conformational variability and increased divergence between replicas.

#### 2.6.4. Molecular Surface Area (MolSA)

The molecular surface area represents the total surface area of the ligand and was analyzed alongside the solvent-accessible surface area (SASA) and polar surface area (PSA) to assess conformational stability and solvent exposure. This parameter remained essentially constant throughout the trajectories for imatinib (486.16 ± 1.28 Å^2^), nilotinib (478.24 ± 1.69 Å^2^), and the two active ferrocene analogues (compound **9**: 431.84 ± 1.78 Å^2^; compound **15a**: 433.71 ± 1.34 Å^2^), indicating the absence of major conformational distortions (Figure S15, Supplementary Materials). In contrast, compound **15e** exhibited a higher mean molecular surface area and substantially greater variability (502.64 ± 23.79 Å^2^), consistent with the broader conformational ensemble sampled across its replicas.

#### 2.6.5. Solvent-Accessible Surface Area (SASA)

The solvent-accessible surface area (SASA) represents the portion of a molecule’s surface that is accessible to solvent [59]. This parameter was lower for both Fc derivatives than for their respective parent drugs (compound **9**: 36.46 ± 6.23 Å^2^ vs. imatinib: 79.42 ± 21.50 Å^2^; compound **15a**: 43.05 ± 8.26 Å^2^ vs. nilotinib: 59.69 ± 17.73 Å^2^), indicating deeper burial within the ATP-binding pocket and reduced solvent exposure (Figure S16, Supplementary Materials). This trend is consistent with the hydrophobic character of the ferrocene (Fc) moiety and with the docking interaction fingerprint analysis, which revealed high-occupancy contacts with the lipophilic back-pocket residues Leu248 (P-loop) and Leu370 (located between the C-spine and activation loop).

The substantially higher SASA variability observed for imatinib (SD = 21.50 Å^2^) further supports its greater positional flexibility, as reflected in its ligand RMSD profile, and may be attributed to the solvent exposure of its piperazine moiety. In contrast, the inactive control compound **15e** remained considerably more solvent-exposed (117.97 ± 24.92 Å^2^) than either parent inhibitor, consistent with its poorer burial within the binding pocket relative to the active analogues.

#### 2.6.6. Polar Surface Area (PSA)

Polar surface area (PSA) is an important descriptor of drug-likeness, quantifying the fraction of a molecule’s surface contributed by polar atoms [60]. The PSA values of all compounds remained below the 140 Å^2^ threshold throughout the simulations, consistent with favorable passive membrane permeability. The Fc derivatives exhibited lower mean PSA values (compound **9**: 66.80 ± 2.01 Å^2^; compound **15a**: 61.66 ± 1.71 Å^2^) than their parent drugs (imatinib: 103.36 ± 1.60 Å^2^; nilotinib: 113.45 ± 2.43 Å^2^). The low standard deviations observed for these compounds indicate stable polar surface exposure and the absence of significant rearrangements of polar functional groups throughout the trajectories (Figure S17, Supplementary Materials).

Compound **15e** exhibited a higher mean PSA and greater inter-replica variability (132.03 ± 19.70 Å^2^), although it remained below the permeability threshold.

#### 2.6.7. Intermolecular Interactions Analysis

**Figure 8:**
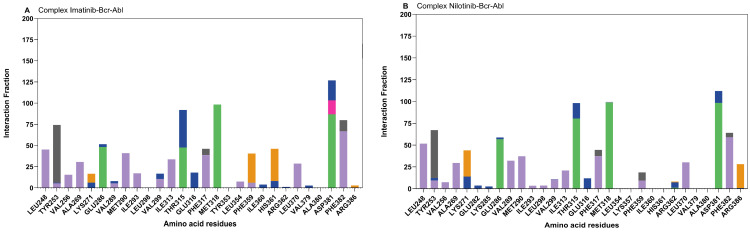

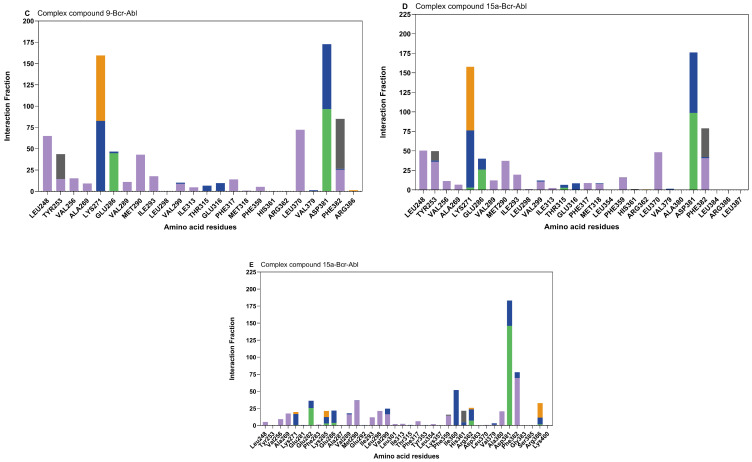
Interaction fingerprint profiles derived from molecular dynamics simulations of ABL kinase complexes with imatinib (**A**), nilotinib (**B**), compound **9** (**C**), compound **15a** (**D**), and compound **15e** (**E**). Stacked bar charts represent the mean interaction occupancy (%) across three independent 500 ns replica simulations. Interaction types are color-coded as follows: hydrogen bonds (green), hydrophobic contacts (light purple), ionic interactions (pink), π–π stacking (gray), water-mediated bridges (blue), and cation–π interactions (orange).

The present section provides an integrated overview of the intermolecular interactions observed across three independent MD replicas for each ligand. Interaction fingerprint analysis of the MD trajectories revealed coherent yet structurally distinct binding profiles for the Fc-containing analogues compared with imatinib (Figure 8). The occupancy of intermolecular interactions is expressed as percentages across the three independent replicas and is reported in detail in Tables S7 and S8 (Supplementary Materials). For clarity, only interactions with occupancies greater than 1% of the simulation time and consistently observed across all three replicas are discussed here; the reported values represent averages across the simulations.

For imatinib, the dominant polar anchor is a persistent backbone hydrogen bond with the hinge residue Met318, in which the pyridine nitrogen accepts a proton from the backbone NH (98.4%). This interaction is characteristic of type II ATP-competitive kinase inhibitors [61,62]. Additional hydrogen bonding is observed with Asp381 (DFG) (87.0%), where the benzamide carbonyl oxygen accepts a hydrogen bond from the backbone NH of Asp381. Concurrently, the protonated piperazine nitrogen donates a hydrogen bond to the side-chain carboxylate of Asp381. A hydrogen bond with the gatekeeper residue Thr315 is present for 47.7% of the simulation time, with the aniline nitrogen donating to the side-chain hydroxyl group of Thr315. This interaction is complemented by a water-mediated contact involving the pyrimidine nitrogen and the Thr315 hydroxyl group (44.3%). Hydrogen bonding with Glu286 (αC) is detected at moderate occupancy (48.3%), with the benzamide NH donating to the side-chain carboxylate. Additional water-mediated contacts are observed with Glu316 (β5, 18.1%), His361 (HRD, 8.0%), Val299 (αC–β4 loop, 6.4%), and Lys271 (β3, 6.2%), involving various ligand heteroatoms bridging backbone or side-chain functionalities. Minor water-bridged contacts (<5%) are also detected with residues across the P-loop, αC helix, HRD motif, DFG region, and A-loop (Table S7, Supplementary Materials).

The protonated piperazine nitrogen of imatinib forms an ionic interaction with the side-chain carboxylate of Asp381 (DFG) (16.4%) and is also expected to participate in cation–π interactions with His361 (38.0%) and Phe359 (34.5%), with weaker contributions from Lys271 (β3, 10.5%) and Arg386 (A-loop, 2.3%). Aromatic interactions are predominantly governed by edge-to-face π–π stacking with Tyr253 (P-loop, 68.3%), with additional, less pronounced contributions from Phe382 (A-loop, 12.9%, both geometries) and Phe317 (hinge, 7.4%). Imatinib also forms extensive hydrophobic contacts, primarily with Phe382 (DFG, 67.1%), Leu248 (P-loop, 45.4%), Met290 (RS3, αC, 41.1%), Phe317 (hinge, 38.8%), Ile313 (β5, 33.8%), Ala269 (β3, 30.7%), and Leu370 (β3, 28.7%), along with additional secondary and minor contacts distributed throughout the binding pocket, including Ile293 (17.2%), Val256 (15.6%), Val299 (10.5%), Leu354 (7.5%), Phe359 (6.0%), Tyr253 (5.5%), and Val289 (5.4%).

For nilotinib, the dominant polar anchor is likewise a persistent backbone hydrogen bond with the hinge residue Met318, in which the pyridine nitrogen accepts a proton from the backbone NH (99.0%), consistent with the canonical type II binding mode. A second high-occupancy hydrogen bond is observed with Asp381 (DFG) (98.5%), where the benzamide carbonyl oxygen accepts a hydrogen bond from the backbone NH of Asp381. Hydrogen bonding with the gatekeeper residue Thr315 is observed for 80.4% of the simulation time, indicating a notably stronger interaction than in imatinib; here, the anilino–pyrimidine linker N–H donates a hydrogen bond to the Thr315 side-chain hydroxyl group. Moderate hydrogen bonding is detected with Glu286 (αC, 56.7%), with the benzamide amide N–H donating to the side-chain carboxylate. A minor contribution involving Arg362, mediated by the methylimidazole nitrogen, is also observed (1.2%).

Water-mediated contacts of notable occupancy are identified with Thr315 (17.9%), Asp381 (13.6%), Lys271 (β3, 14.0%), and Glu316 (β5, 12.1%), with minor contributions from Glu282, Lys285, Glu286, Val299, Arg362, and Phe382 (<5%).

Unlike imatinib, nilotinib lacks a protonatable piperazine nitrogen and therefore does not form ionic interactions with Asp381 (DFG). Aromatic interactions are dominated by face-to-face and edge-to-face π–π stacking with Tyr253 (P-loop, 55.0%), with weaker contributions from Phe359 (9.4%), Phe317 (hinge, 7.1%), and Phe382 (4.9%). Cation–π interactions are observed with Lys271 (β3, 30.0%) and Arg386 (A-loop, 27.4%).

Nilotinib forms extensive hydrophobic contacts, primarily with Phe382 (DFG, 59.1%), Leu248 (P-loop, 51.8%), Met290 (RS3/αC, 37.2%), Phe317 (hinge, 37.4%), Val289 (32.2%), Leu370 (β3, 30.4%), Ala269 (β3, 29.6%), and Ile313 (β5, 20.9%), along with secondary contributions from Val299 (10.6%), Tyr253 (9.4%), Phe359 (9.3%), Val256 (7.5%), Ile293 (3.5%), and Leu298 (3.6%).

For compound **9**, hydrogen bonding involves two principal residues. The dominant polar anchor is a persistent interaction with Asp381 (DFG, 96.5%), in which the backbone NH donates a hydrogen bond to the ligand carbonyl oxygen. A secondary hydrogen bond with Glu286 (αC) is observed at moderate occupancy (44.9%), involving donation from the benzamide NH to the side-chain carboxylate.

Water-mediated interactions are prominent, particularly with Lys271 (β3, 82.8%) and Asp381 (DFG, 76.3%). In both cases, the pyrimidine nitrogen, benzamide carbonyl oxygen, and benzamide NH participate in bridging interactions with the respective side chains. Additional water bridges occur with Glu316 (β5, 9.8%) and Thr315 (6.6%), with minor contributions from Glu286 (αC), Val379 (A-loop, near the DFG motif), Val299 (αC–β4 loop), and Phe382 (DFG) (<2%).

Aromatic interactions are primarily dominated by π–π stacking with Phe382 (58.7%, involving both edge-to-face and face-to-face geometries) and Tyr253 (29.3%, edge-to-face), predominantly mediated by the pyrimidine ring. The phenyl ring is also expected to form a strong cation–π interaction with Lys271 (76.7%), with only minor contributions from Arg386 (1.4%).

Compound **9** maintains an extensive hydrophobic contact network, with primary contacts involving Leu370 (A-loop) (72.3%), Leu248 (P-loop) (65.0%), Met290 (RS3/αC) (43.1%), and Phe382 (DFG) (25.4%). Additional contacts of intermediate and low occupancy are distributed across residues in the P-loop (Val256, 15.3%, and Tyr253, 14.4%), αC helix (Ile293, 17.8%, and Val289, 11.2%), hinge region (Phe317, 14.1%), β3 (Ala269, 9.3%), β5 (Ile313, 4.8%), αC–β4 loop (Val299, 8.9%), and HRD vicinity (Phe359, 5.3%).

For compound **15a**, Asp381 (DFG) again serves as the primary polar anchor, with the amide carbonyl oxygen accepting a hydrogen bond from the backbone NH (98.6%). The hydrogen bond with Glu286 is retained at lower occupancy (26.1%), involving both backbone and side-chain interactions. Notably, direct hydrogen bonding with the hinge residue Met318 is absent, and interaction with the gatekeeper residue Thr315 is negligible (2.8%), confirming the loss of canonical hinge and gatekeeper contacts. A minor direct hydrogen bond with Lys271 (2.9%) is also observed.

Water-mediated interactions form a key compensatory network. The most prominent involve Asp381 (77.6%) and Lys271 (73.3%), mediated primarily by the pyrimidine nitrogen. Additional water bridges are observed with Glu286 (αC, 13.9%) and Glu316 (β5, 8.5%), with minor contributions from Thr315, Val379, Tyr253, Phe382, and Val299.

A defining feature of compound **15a** is a high-occupancy cation–π interaction between the 3-methylphenyl ring and Lys271 (β3, 81.5 ± 5.7%), substantially exceeding that observed for imatinib. π–π stacking is dominated by Phe382 (DFG, 36.7%, both geometries), with secondary contributions from Tyr253 (P-loop, 12.1%) and negligible interaction with His361 (HRD, 1.1%). Hydrophobic contacts are extensive, particularly with Leu248 (P-loop, 50.5%), Leu370 (β3, 48.3%), Phe382 (DFG, 40.7%), Met290 (RS3/αC, 37.3%), and Tyr253 (P-loop, 36.1%), along with additional interactions across the αC helix. Notably, the relatively high standard deviations for interactions with Leu370 (±20.7%) and Phe359 (±21.7%) indicate considerable conformational variability of compound 15a within the binding pocket across simulation replicas.

For compound **15e**, the negative-control analogue, the interaction fingerprint is markedly destabilized and reduced to a single persistent anchoring interaction. Direct hydrogen bonding is dominated by donation from the Asp381 (DFG) backbone NH to an aminopyrimidine nitrogen (88.8 ± 4.8%), representing the only high-occupancy and low-variance polar contact in the entire profile. All remaining direct hydrogen-bonding interactions are weak and highly variable across replicas, including a phenolic hydroxyl group donating to the Asp381 backbone carbonyl (43.5 ± 34.1%), an amide N–H interaction with the Asp381 side-chain carboxylate (12.5 ± 12.1%), and an additional weak interaction between the phenolic hydroxyl group and Glu282 (αC helix) (13.6 ± 10.2%). No hydrogen-bonding interactions with the hinge residue Met318 were observed, while interactions with the gatekeeper residue Thr315 were negligible. The β3 lysine Lys271 is essentially disengaged across all interaction types, indicating a complete loss of both canonical hinge/gatekeeper anchoring and the non-classical Lys271-mediated stabilization observed for the active compounds.

The remaining interactions are redistributed away from the β3/P-loop region toward the DFG, HRD, and activation-loop regions. Water-mediated contacts, which in compounds **9** and **15a** are predominantly centered on Lys271, are instead distributed mainly to the Ile360 backbone (40.1 ± 20.2%), the Asp381 side-chain carboxylate (21.1 ± 13.0%), and Phe382 (DFG), all exhibiting variances comparable to their mean occupancies. The strong Lys271 cation–π interaction observed in the active compounds collapses to 3.0 ± 2.8%. The dominant cation–π interaction in **15e** is instead formed between the distal 4-hydroxyphenyl ring and Arg386 (activation loop) (21.3 ± 8.9%). π–π stacking interactions are weak and largely confined to His361 (HRD motif) (16.8 ± 4.5%), whereas the Phe382 (DFG) and Tyr253 (P-loop) stacking interactions characteristic of the active compounds are absent.

Hydrophobic contacts remain numerous and are primarily contributed by Phe382 (DFG) (69.7 ± 6.4%) and Met290 (RS3/αC) (36.8 ± 9.0%), but P-loop-associated hydrophobic interactions—particularly with Leu248 and Tyr253—are essentially lost (5.3 ± 4.6% and 0.1%, respectively).

To explore a potential mechanism of inhibition via ligand trapping—recently proposed by our group [55,56,57,58]—in which stabilization of the P-loop prevents dissociation of the protein–ligand complex, additional analyses were performed. The P-loop remained predominantly in a closed conformation throughout the MD simulations of ABL complexes with compounds **9** and **15a**, prompting a more detailed analysis of interactions between the Fc moiety and residues within the P-loop. In these systems, the Fc moieties of compounds **9** and **15a** come into proximity with the phenyl ring of Tyr253 from the P-loop, suggesting the possibility of π–π or cation–π interactions with this residue.

Accordingly, distances between the centroids of the cyclopentadienyl (Cp) rings and the phenyl ring of Tyr253, as well as between the Fe^2+^ ion and the Tyr253 ring centroid, were calculated, along with the angle between the corresponding ring planes. To characterize these contacts beyond centroid separation, the minimum inter-ring carbon–carbon distances, lateral offset, and projected ring overlap were additionally evaluated. A permissive centroid–centroid distance threshold of 5.5 Å and an interplanar angle range of 0–90° were used to identify geometries compatible with potential π–π interactions. Additionally, Fe^2+^–aromatic proximity was assessed using a distance threshold of ≤6.0 Å as a criterion for possible cation–π interactions. These thresholds were selected based on literature-reported geometric criteria and were interpreted alongside interaction persistence and ring orientation parameters [63,64,65,66].

**Figure 9:**
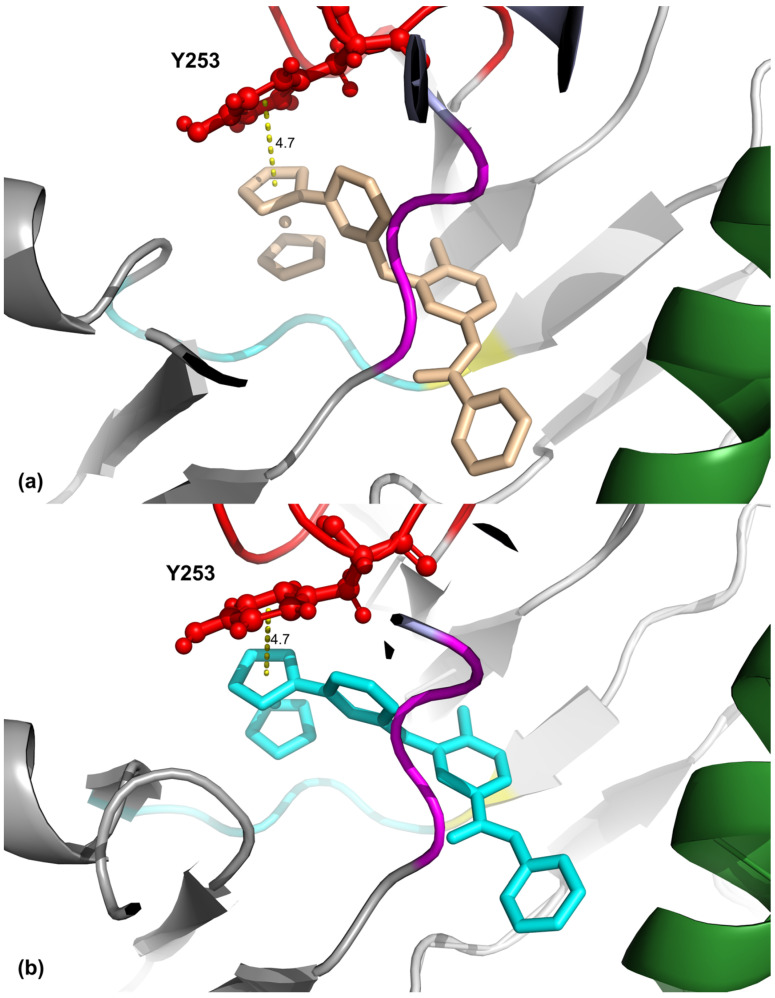
Representative medoid structures of the dominant binding-site clusters for compound **9** (sand color) (**a**) and compound **15a** (cyan) (**b**). Protein structural elements are colored as follows: the P-loop (including Tyr253, shown as ball-and-stick) in red, the αC-helix in green, the DFG motif in magenta, and the hinge region in cyan. Thr315 is highlighted in yellow. (**a**) Compound **9**, medoid at 304.8 ns from replica 1, corresponding to the most populated cluster; (**b**) Compound **15a**, medoid from replica 3, corresponding to the dominant cluster with 42.8% population. Dashed lines indicate the measured distances (Å) between the center of mass of the monosubstituted cyclopentadienyl ring of the ferrocene moiety (Cp1) and the Tyr253 phenyl ring.

For compound **9**, consistent and reproducible proximity was observed between the substituted cyclopentadienyl ring (Cp1) and the phenyl ring of Tyr253 across all three simulations. In the first replica, the Cp1–Tyr253 centroid distance was ≤5.5 Å for approximately 426.5 ns (85.29% of the trajectory), supporting a potential π–π interaction (Figure 9). The mean interplanar angle was 38.63°, and the closest inter-ring carbon–carbon distances averaged ~4.0 Å, together indicating a slipped or intermediate configuration between parallel and T-shaped orientations. The unsubstituted Cp2 ring showed negligible involvement. The Fe–Tyr253 centroid distance was ≤6 Å for approximately 36.4 ns (7.28% of the trajectory) and remained below 10 Å for most of the simulation, suggesting occasional geometries compatible with cation–π interactions. Similar trends were observed in the second and third replicas, with Cp1–Tyr253 proximity maintained for 77.48% and 84.12% of the simulation time, respectively, and mean interplanar angles of ~39°. Cp2 remained minimally involved, while Fe–Tyr253 distances ≤6 Å were observed intermittently (6.2% and 8.48% of simulation time, respectively).

For compound **15a**, all simulations demonstrated sustained proximity between Cp1 and Tyr253, with distances ≤5.5 Å for 74.0%, 80.92%, and 85.24% of the simulation time across the three replicas. Mean interplanar angles ranged from ~39° to ~49°, and the corresponding minimum inter-ring carbon–carbon distances (~3.7–3.9 Å) confirmed genuine close contacts consistent with π–π interactions. Unlike compound **9**, the Cp2 ring exhibited occasional proximity to Tyr253, with occupancies of 17.16%, 6.08%, and 0.32% across the three replicas, suggesting transient additional aromatic contacts. The Fe^2+^ center remained within 10 Å of Tyr253 throughout the simulations. It was within ≤6 Å for 49.52%, 25.0%, and 12.12% of the simulation time, respectively, indicating more frequent geometries compatible with potential cation–π interactions compared with compound **9**.

By contrast, this proximity was absent in the inactive analogue **15e**: the Cp1–Tyr253 centroid distance remained approximately 9.3–9.5 Å across all replicas, with 0% of frames sampling distances ≤5.5 Å. The Fe center similarly remained at ~8.5–9.3 Å from Tyr253, and no frames satisfied either the π–π or cation–π geometric criteria. This behavior is consistent with the sustained P-loop opening observed for this compound and supports the interpretation that Tyr253-mediated anchoring is a specific feature of the active leads.

#### 2.6.8. Trajectory-Based MM-GBSA Analysis

**Figure 10:**
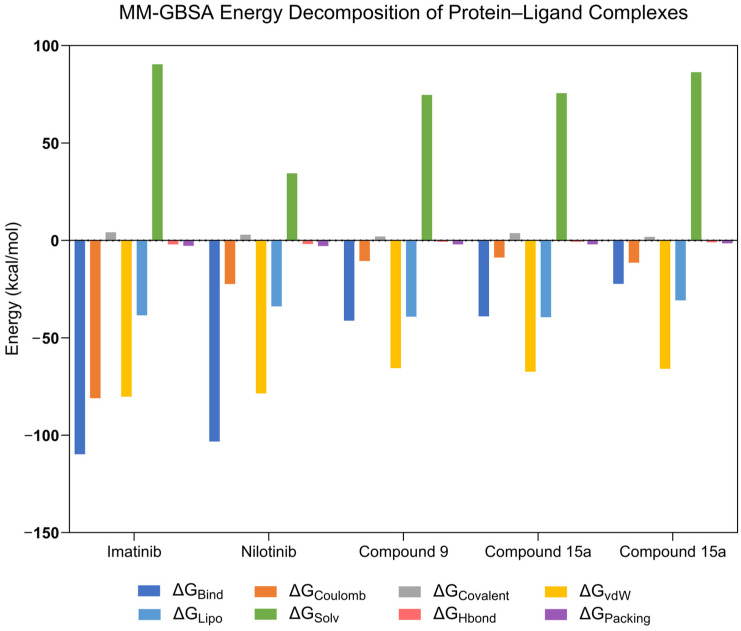
MM-GBSA energy decomposition profiles of the BCR-ABL1 complexes with imatinib, nilotinib, compound **9**, compound **15a**, and compound **15e**, calculated from the last 100 ns of the molecular dynamics simulations. Bars represent the overall mean values averaged across the three independent replicas for the total MM-GBSA binding-energy estimate (ΔG_bind_) and its individual components, including Coulombic, covalent, van der Waals, lipophilic, solvation, hydrogen-bonding, and packing terms.

MM-GBSA calculations, together with energy decomposition analyses, were performed over the final 100 ns of the simulations. Mean values are presented in Figure 10, while detailed results are provided in Table S9 (Supplementary Materials).

Within this computational framework, the BCR-ABL1–imatinib complex showed the most favorable trajectory-based MM-GBSA estimate (ΔG_bind_ = −109.82 ± 6.73 kcal/mol). This reflects substantial contributions from van der Waals and electrostatic interactions, followed by lipophilic effects, which are partially offset by the polar solvation term. Nilotinib, a second-generation BCR-ABL1 inhibitor, exhibited a similarly favorable MM-GBSA profile (ΔG_bind_ = −103.22 ± 3.13 kcal/mol), dominated primarily by van der Waals interactions, with a lower electrostatic contribution relative to imatinib, consistent with its neutral protonation state under the simulation conditions.

The Fc-containing derivatives, compounds **9** and **15a**, displayed less favorable but comparable trajectory-based MM-GBSA estimates, with mean ΔG_bind_ values of approximately −40 kcal/mol for both compounds. Direct quantitative comparison with the reference inhibitors should be interpreted with caution, as reflected in the methodological discussion of the limitations of MM-GBSA for systems containing organometallic fragments. In particular, the more favorable ΔG_bind_ value observed for imatinib is influenced by its protonated state under the simulation conditions, which enhances electrostatic contributions. In addition, the η^5^ Fe–Cp electronic structure of the ferrocene moiety is not explicitly represented in a manner fully optimized for quantitative free-energy estimation within the MM-GBSA/OPLS4 framework. As a result, the reported ΔG_bind_ values should be considered semi-quantitative and primarily suitable for relative ranking within the same computational protocol, rather than as absolute binding free-energy estimates.

Among the ferrocene-containing derivatives, compound **15e** exhibited less favorable ΔG_bind_ values than the active leads **9** and **15a** within the same computational framework (−22.27 ± 2.80 kcal/mol). Energy decomposition analysis indicates that its binding is predominantly dispersion-driven, with a strong van der Waals contribution (−65.88 kcal/mol), a modest electrostatic term (−11.44 kcal/mol), and a negligible hydrogen-bonding contribution (−0.98 kcal/mol). These favorable interactions are strongly counterbalanced by a substantial polar solvation penalty (+86.40 kcal/mol), higher than that observed for nilotinib (+34.48 kcal/mol), consistent with inefficient desolvation of polar groups that remain partially exposed at the binding-site periphery.

Importantly, these MM-GBSA-derived trends are interpreted in a semi-quantitative manner and are not used as the sole basis for mechanistic conclusions. Instead, they are considered in conjunction with trajectory-based analyses. In this context, compound **15e** shows a consistent pattern of reduced binding-site burial, loss of Lys271/Tyr253 anchoring interactions, and diminished stabilization of the P-loop region compared with compounds **9** and **15a**, in agreement with the molecular dynamics results.

## 3. Discussion

The integrated computational workflow employed in this study provides an atomistic framework for rationalizing the observed differences in cytotoxicity among Fc-containing BCR-ABL1 inhibitor analogues.

Retrospective validation of the docking protocol using a combined DUD-E and DeepCoy decoy set yielded an AUC of 0.82 and an enrichment factor of 35.0 at 1% of the dataset. The final benchmark set (51 actives and 5542 decoys; ~1:109) is therefore highly stringent. While DUD-E decoys may introduce bias due to physicochemical dissimilarity, DeepCoy generates structurally similar decoys, thereby reducing trivial discrimination [52,53]. This combined strategy aligns with recent recommendations for robust virtual screening validation [67] and supports the conclusion that the protocol captures meaningful BCR-ABL1 recognition features rather than artifacts of decoy selection [67]. Inactive analogues (compounds **3** and **15b**–**e**) failed to establish productive interaction networks, instead adopting peripheral binding modes and exhibiting unfavorable MM-GBSA energies. The partial activity observed for compound **15b** likely reflects retention of aromatic character, enabling limited hydrophobic interactions. Overall, the docking protocol, supported by qualitative MM-GBSA estimates, broadly separated the most active compounds from weaker analogues within the studied series.

Molecular dynamics (MD) simulations of the active compounds (**9** and **15a**), the inactive control analogue **15e**, and the reference inhibitors imatinib and nilotinib were performed using three independent 500 ns replicas per system. All complexes exhibited stable and compact behavior, as evidenced by protein Cα RMSD and RMSF profiles, ligand heavy-atom RMSD, and radius of gyration. In addition, molecular surface area, solvent-accessible surface area (SASA), and polar surface area analyses consistently supported stable ligand burial within the ATP-binding site.

Docking and MD simulations demonstrated that compounds **9** and **15a** occupy the ATP-binding pocket in a manner broadly similar to imatinib, yet with distinct interaction profiles. Despite lacking the canonical hinge hydrogen bond with Met318, both compounds maintained stable binding modes across all simulations, with ligand RMSD values ranging from ~1.6 Å to ~1.9 Å. This indicates that hinge engagement is not strictly required for stable complex formation in this chemical series. A similar phenomenon has been reported for a potent ABL1 switch-control inhibitor that forgoes hinge interactions and instead anchors to the activation loop, achieving nanomolar potency through alternative polar contacts [68]. The alternative interaction pattern likely reflects the bulkier, more three-dimensional character of the Fc moiety compared with the planar aromatic scaffolds typical of classical tyrosine kinase inhibitors. The relatively high DFT-derived logP values observed for compounds **9** and **15a** further indicate pronounced hydrophobic character, which may facilitate favorable interactions within the predominantly hydrophobic ATP-binding pocket.

Both Fc-containing analogues anchor primarily to Asp381 of the DFG motif, which serves as the dominant polar interaction site. High-occupancy cation–π interactions with Lys271 appear to compensate for the absence of hinge contacts and may contribute to stabilization of the inactive DFG-out conformation via the conserved Lys271–Glu286 interaction network. This interaction is mechanistically significant, as the Lys271–Glu286 salt bridge is a conserved structural feature of the N-lobe and is closely associated with DFG motif positioning [69,70].

Differences between compounds were observed in their interactions with Thr315 and in their overall conformational flexibility. Compound **9** showed negligible interaction with Thr315, whereas compound **15a** exhibited minor contact. This distinction may have implications for differential sensitivity to the T315I gatekeeper mutation. Consistently, compound **9** displayed more rigid binding behavior, while compound **15a** exhibited greater conformational variability, reflected in higher activation-loop RMSF values.

To explore a potential mechanism of inhibition, particular attention was focused on the P-loop region. Previous studies by Spassov and colleagues proposed that a closed P-loop conformation can facilitate ligand retention within the catalytic cleft, whereas an open conformation promotes dissociation [55,56,57,58]. In the present study, P-loop dynamics remained stable in complexes with compounds **9** and **15a**, with no evidence of large-scale opening events. Compound **9** exhibited slightly lower and more reproducible P-loop RMSD values compared with imatinib, whereas compound **15a** showed moderately increased flexibility. These observations suggest that P-loop stabilization may contribute to a trapping-like inhibition mechanism. In contrast, the inactive analogue compound **15e** exhibited markedly higher and more variable P-loop RMSD values, indicating reduced stabilization of this region relative to the active compounds. This clear contrast between the stable P-loop behavior of the active analogues and the increased flexibility of the weakly active analogue is consistent with the proposed ligand trapping mechanism, whereby effective inhibitors restrict P-loop mobility, whereas loss of P-loop stabilization is associated with reduced activity.

Interaction analysis provides a structural explanation for this behavior. Like the active analogues, compound **15e** retains the primary Asp381 (DFG) backbone interaction; however, it loses the two interactions most strongly associated with P-loop stabilization in compounds **9** and **15a**. In particular, the Lys271 cation–π interaction decreases from ~77–82% occupancy in the active compounds to ~3% in **15e**, and the π–π and hydrophobic interactions with Tyr253 are absent. As a result, the ligand is primarily anchored through its aminopyrimidine–ferrocenyl moiety, while interactions with the P-loop are largely lost.

Without these, the ligand is held almost entirely by its aminopyrimidine–ferrocenyl end. The RMSF profile shows the same picture: the anchored end stays restrained (~0.6–1.0 Å), whereas the distal part reaches ~3.9 Å. With its contacts shifted away from the P-loop towards the activation loop and HRD motif, and with large variability between replicas. This displaced binding mode accounts for the loss of Tyr253-mediated P-loop stabilization and, ultimately, for the reduced activity.

P-loop mutations associated with clinical resistance are frequently observed in patients treated with imatinib, including substitutions at Tyr253 (e.g., Y253F and Y253H) [71,72]. The observation that the Fc moiety of compounds **9** and **15a** can form π–π and cation–π interactions with this residue suggests a potential strategy to mitigate resistance conferred by these mutations, as well as other P-loop alterations.

Finally, the Fc-containing analogues exhibited distinct binding behavior at two key mutation hotspots. The minimal interaction with the gatekeeper residue Thr315 suggests potential differences in sensitivity to the T315I mutation. In parallel, the presence of π–π and cation–π interactions with the P-loop residue Tyr253 indicates a possible strategy to counteract resistance associated with P-loop mutations. Together, these features highlight a dual mechanistic aspect of Fc-based binding, involving both reduced dependence on the gatekeeper position and enhanced engagement of the P-loop region. Future work should include explicit modeling of mutant ABL1 variants and experimental validation to further assess these hypotheses.

## 4. Materials and Methods

### 4.1. Ligand and Receptor Modeling

Imatinib and nilotinib analogues containing an Fc moiety were constructed using the open-source molecular builder and visualization tool Avogadro v. 1.2.0 [73,74]. To obtain a physically realistic representation of Fe–cyclopentadienyl bonding, geometry optimization was performed using density functional theory (DFT) as implemented in ORCA v.6.1.0 (FACCTs GmbH, Cologne, Germany) [75]. The B3LYP hybrid functional was employed in combination with the def2-TZVP basis set, which is suitable for metal–organic systems, together with the def2/J auxiliary basis and the RIJCOSX approximation to accelerate the calculations [76,77,78,79,80]. Dispersion interactions were accounted for using the D3BJ correction [81,82]. All geometry optimizations were performed using the VeryTightOpt convergence criteria in ORCA v.6.1.0 [75]. To approximate aqueous conditions, the SMD (Solvation Model based on Density) implicit solvation model for water was applied during geometry optimization, yielding energetically favorable and physically meaningful conformations [83]. Frequency calculations at the same level of theory confirmed that the optimized structures correspond to true local minima on the potential energy surface.

To further validate the accuracy of the Fc geometry, a structural comparison with crystallographic data was performed. A reference Fc fragment was extracted from the ligand in the 5MYQ crystal structure and used as an experimental model [51]. The Fc moieties of the optimized compounds were aligned to this fragment, and the root-mean-square deviation (RMSD) was calculated.

To provide an additional assessment of drug-likeness, the octanol/water partition coefficient (logP) was estimated using a quantum-chemical approach. Single-point energy calculations were performed for the optimized gas-phase geometries and in implicit water and n-octanol using the SMD solvation model implemented in ORCA v.6.1.0 [75]. Solvation free energies were calculated from the energy differences between solvent and gas-phase calculations. The octanol/water partition coefficient was subsequently derived from the difference between the calculated solvation free energies in water and n-octanol. In addition, molecular weight (MW), hydrogen-bond donor (HBD), and hydrogen-bond acceptor (HBA) counts were evaluated to assess compliance with Lipinski’s Rule of Five.

The crystallographic structure of the human Abl1 kinase domain in complex with imatinib (PDB ID: 2HYY) was selected as the receptor model [50]. Missing loop regions (Glu275–Thr277) and incomplete side chains were reconstructed using Prime (release 2025-4, Schrödinger, LLC, New York, NY, USA, 2025) [84,85]. The structure was subsequently prepared using the Protein Preparation Workflow in Schrödinger Release 2025-4, including the addition of hydrogen atoms, assignment of protonation states at pH 7.4, and restrained energy minimization of hydrogen atoms [86].

### 4.2. Molecular Docking Protocol

Docking calculations were carried out using the Glide Standard Precision (SP) protocol, with the protein treated as rigid and ligands treated as flexible [87]. The receptor grid was generated in Glide (release 2025-4, Schrödinger, LLC, New York, NY, USA, 2025) and centered on the centroid of the co-crystallized ligand imatinib [87,88]. Ligand sampling included nitrogen inversions, ring conformations, and torsional sampling, with bias applied to predefined functional groups. Epik state penalties were incorporated into the docking score to account for the relative populations of different protonation states [89]. For the ferrocene-containing ligands, the Fe–Cp sandwich arrangement was preserved in the input docking geometries through explicit Fe–C connectivity to the cyclopentadienyl rings. These Fe–C interactions were defined as zero-order bonds, with partial charges assigned to the iron center based on the DFT-optimized structures. The internal geometry of the ferrocene unit was maintained close to the DFT reference using distance restraints. Accordingly, the fragment was treated as an effectively rigid body during preparation, preserving the η^5^ sandwich architecture while allowing the remainder of each ligand to explore its conformational space.

The inner grid box size ranged from 10 to 14 Å to optimize grid dimensions. Ligands were ranked by predicted binding affinity using GlideScore, an empirical scoring function that incorporates protein–ligand Coulomb and van der Waals interactions, hydrogen bonding, lipophilic contacts, penalties for ligand flexibility, and hydrophobic enclosure effects [87].

### 4.3. Docking Protocol Validation

Protocol validation was initially performed by re-docking imatinib, and the root-mean-square deviation (RMSD) between the docked and crystallographic poses was calculated to assess the accuracy of pose reproduction. Further validation was conducted using a combined active/decoy dataset comprising 4600 decoy molecules obtained from the DUD-E library for ABL1 [52]. The number of DUD-E decoys was chosen to preserve a realistic active-to-decoy ratio for retrospective screening and to ensure stable estimation of early-enrichment metrics. In addition, 600 structure-matched decoys were generated using DeepCoy [53], designed to reproduce the physicochemical properties of the Fc derivatives while remaining topologically distinct, thereby minimizing artificial enrichment due to trivial property-based separation.

As Fc is not natively supported, the Fc moiety in the test compounds was replaced in the SMILES representation by aromatic hydrocarbon linkers with comparable molecular weight and lipophilicity. All 21 SMILES structures were used as input for DeepCoy, and duplicate entries were removed using Open Babel v. 3.1.1, yielding 597 unique decoys [90]. This substitution was applied exclusively for decoy generation and was not used during docking or evaluation of the test compounds.

A set of 50 ABL1 inhibitors was selected from the ChEMBL database (release 36) [91], ensuring structural diversity relative to the investigated Fc analogues; these compounds are hereafter referred to as actives. The combined dataset (50 actives, 4600 DUD-E decoys, and 597 DeepCoy decoys; total = 5247) was prepared using LigPrep (release 2025-4, Schrödinger, LLC, New York, NY, USA, 2025), generating relevant ionization states and tautomers at pH 7.4. This resulted in an expanded set of structures subjected to docking and subsequent ROC analysis. All molecules were processed using the same docking protocol described above, with a single top-ranked pose retained per ligand for validation runs and imatinib re-docking. Enrichment performance was then evaluated using ROC curves, AUC, BEDROC, and enrichment factors, as implemented in Schrödinger Release 2025-4 (Glide) [92].

### 4.4. Docking Analysis

Following protocol validation, all previously prepared Fc-containing ligands were docked alongside the reference inhibitor imatinib. For the test compounds, post-docking minimization was applied to the top 50 poses per ligand, and per-residue interaction scores were recorded. The highest-ranked pose based on GlideScore was selected for each ligand for further analysis and subsequent molecular dynamics simulations. To obtain a detailed characterization of binding interactions, protein–ligand interaction fingerprint (IFP) analysis was performed on the selected poses [93]. A generative artificial intelligence tool (Claude Opus 4.6, Anthropic; https://claude.ai; accessed on 15 February 2026) was used to assist in the development of a Python v. 3.13 script for graphical visualization of the IFP data; all outputs were reviewed and validated by the authors.

### 4.5. MM-GBSA Calculations

Binding free-energy estimates were obtained using the MM-GBSA method as implemented in Prime (release 2025-4, Schrödinger, LLC, New York, NY, USA, 2025) [94]. Calculations employed the variable-dielectric generalized Born (VSGB 2.1) solvation model in combination with the OPLS4 force field [45,94,95]. Residues within 5 Å of the ligand were treated as flexible, while the remainder of the protein was kept rigid. The MM-GBSA results were used exclusively to refine docking poses and to prioritize ligands for subsequent molecular dynamics simulations. For Fc-containing ligands, these values were used only as supportive, semi-quantitative ranking metric within the series, given the limitations of conventional MM-GBSA scoring for η^5^ Fe–Cp organometallic systems.

### 4.6. Molecular Dynamics Simulations

Molecular dynamics simulations were performed for the two most active Fc-containing analogues **9** and **15a**, the inactive analog **15e**, and the reference inhibitors imatinib and nilotinib, using Desmond (release 2025-4, Schrödinger, LLC, New York, NY, USA, 2025) [96]. The OPLS4 force field was applied throughout [45]. Within the classical force-field representation, the ferrocene unit is described using zero-order Fe–C connectivity to the cyclopentadienyl rings, with the metallocene constrained to remain close to its DFT-optimized geometry via distance restraints. This approach reproduces the experimentally observed sandwich-like architecture of the fragment and maintains its structural stability throughout the MD simulations, effectively treating it as a rigid body, while the electronic delocalization intrinsic to η^5^ coordination lies beyond the formal scope of a classical force field.

System neutrality was achieved by adding Na^+^ counterions, followed by NaCl to a final concentration of 0.15 M to approximate physiological ionic strength. Energy minimization was first performed using the steepest descent algorithm. The systems were then equilibrated using the default Desmond relaxation protocol, consisting of five stages: (i) Brownian dynamics under NVT conditions at 10 K with restraints on solute heavy atoms (50 kcal·mol^−1^·Å^−2^, 100 ps), (ii) NVT simulation at 10 K with restraints (12 ps), (iii) NPT simulation at 10 K with restraints (12 ps), (iv) NPT simulation at 300 K with restraints (12 ps), and (v) unrestrained NPT simulation at 300 K (24 ps). Prior to the production phase, each system was solvated in an orthorhombic simulation box containing TIP3P water molecules [97]. Production simulations were carried out in the NPT ensemble at 300 K and 1 atm. Temperature and pressure were controlled using the Nosé–Hoover thermostat (τ = 1 ps) and the Martyna–Tobias–Klein barostat (τ = 2 ps) with isotropic coupling [98,99,100]. Short-range electrostatic interactions were truncated at 9.0 Å, while long-range electrostatics were treated using the particle mesh Ewald (PME) method [101].

For each protein–ligand complex, three independent 500 ns simulations were performed using different random seeds while maintaining identical system setups. Trajectory frames were saved every 200 ps, yielding 2500 frames per replica. Trajectory analysis was conducted using the Simulation Interaction Diagram (SID) tool in Desmond, with the initial frame used as reference. The following metrics were evaluated: protein and ligand RMSDs, protein RMSF, radius of gyration (Rg), molecular surface area (MolSA), solvent-accessible surface area (SASA), polar surface area (PSA), and protein–ligand interaction fingerprints. Interaction occupancies were calculated as the percentage of simulation time during which each interaction was maintained. Mean values and standard deviations were computed across the three replicas and are reported as consolidated results.

Prior to root-mean-square deviation (RMSD) and root-mean-square fluctuation (RMSF) calculations, each trajectory frame was aligned to a reference structure using least-squares fitting on the Cα atoms of the structured kinase core (residues 235–500), excluding the activation loop and highly flexible terminal regions. This procedure ensured that the reported metrics reflect internal conformational dynamics rather than overall rigid-body motion. Protein backbone RMSD and per-residue RMSF were then computed on the same Cα-aligned trajectories. For the P-loop, loop-specific RMSD and per-residue RMSF were calculated using P-loop Cα atoms. To confirm that the resulting flexibility profiles were not biased by the choice of alignment region, the analysis was repeated using two independent superposition schemes: (i) alignment on all kinase-domain Cα atoms, and (ii) alignment on the structured-core Cα subset, excluding flexible N- and C-terminal segments (residues < 235 and >500) as well as the activation loop (residues 381–401).

Both RMSD and RMSF results were consistent across alignment protocols, with only negligible quantitative differences and identical qualitative trends.

Cluster analysis was performed using the MDTraj v. 1.11.1 library on Cα atoms of the kinase domain [102]. Agglomerative hierarchical clustering with average linkage was applied to a pairwise RMSD distance matrix, using a cutoff of 2.0 Å (0.2 nm). The binding site was defined dynamically as the union of all protein atoms located within 5.0 Å of any ligand atom across all trajectory frames. The full trajectories (2500 frames per simulation) were analyzed. For each cluster, the representative structure (medoid) was defined as the frame with the lowest average RMSD to all other cluster members. Cluster populations were expressed as the percentage of frames assigned to each cluster, providing a quantitative measure of conformational stability. Representative structures from the most populated clusters were extracted for structural comparison and visualization.

To assess the reliability of the ferrocene representation during the molecular dynamics simulations, an additional geometric validation procedure was performed for compounds **9**, **15a** and **15e**. Internal ferrocene geometric descriptors were monitored throughout all three independent 500 ns MD replicas and compared against the corresponding DFT-optimized pre-docking reference structures obtained at the B3LYP/def2-TZVP level of theory. The analyzed descriptors included Fe–C bond distances, Fe–cyclopentadienyl (Cp) centroid distances, Cp–Cp centroid distance, interplanar Cp-ring angle, Cp-ring planarity, and rotational offset between the two Cp rings. To account for the fivefold symmetry of the cyclopentadienyl rings, symmetry-equivalent atom permutations were explicitly considered using a Kabsch-based superposition procedure prior to RMSD calculations [103]. In addition, the ferrocene core was compared against an independent crystallographic ferrocene reference fragment extracted from PDB ID 5MYQ [51].

Inter-replica consistency and conformational sampling were additionally evaluated using principal component analysis (PCA), cosine content analysis, and free-energy landscape (FEL) reconstruction. All trajectory processing was performed with MDTraj v.1.11.1 [102]. For each system, the structured kinase core was defined as the Cα atoms of residues 235–500, excluding the activation loop (residues 381–401) and the flexible N- and C-terminal segments, to prevent highly mobile regions from dominating the analysis. The three independent 500 ns replicas of each system were first aligned to a common reference structure (the first frame of replica 1) using this structured-core Cα selection.

Principal component analysis (PCA) was then performed separately for each protein–ligand system. For each system, conformations from all three replicas were combined into a single ensemble, with an equal number of frames contributed by each replica to ensure uniform weighting. The pooled dataset was treated as a collection of independent structural snapshots, and temporal ordering was not used in the construction of the covariance matrix. The covariance matrix was computed over the combined ensemble and diagonalized to obtain system-specific principal components. The coordinates were mean-centered prior to diagonalization. This procedure ensured that all replicas contributed equally to the definition of the conformational space and enabled direct comparison of their sampling within a shared PCA framework. The first two principal components (PC1 and PC2) were retained for analysis and visualization.

Each replica was subsequently projected onto the same system-specific PC1/PC2 space obtained from the pooled ensemble. This allowed direct comparison of conformational sampling across replicas within a common coordinate system. Because PCA was performed separately for each system, absolute PC values are only meaningful within the same system, whereas comparisons between systems were restricted to qualitative features such as distribution overlap, compactness, dispersion, and basin structure.

Cosine content was computed separately for each replica by projecting it onto the shared PC1 and PC2 coordinates, following the definition of Hess [104]. Values approaching unity indicate diffusion-like motion along a given principal component, whereas values approaching zero indicate more constrained sampling. Because cosine content is scale-invariant, it was calculated directly from the projected time series.

Free-energy landscapes were reconstructed for each system using the combined projections of all three replicas onto the PC1/PC2 space. A two-dimensional probability density was estimated on an 80 × 80 grid, and the relative free energy was computed as ΔG = −kBT ln(P), where P is the normalized probability density. The most populated region corresponds to the global minimum of the free-energy landscape. Unsampled regions were left undefined. For visualization purposes only, free-energy values were capped at 20 kJ mol^−1^, without affecting the position of minima.

Cosine-content values were used as a replica-level check of sampling behavior along PC1 and PC2, whereas the pooled FELs were used to identify dominant basins and assess conformational heterogeneity within each system.

For each replica, MM-GBSA calculations were performed on snapshots extracted from the final 100 ns of the production phase at 1 ns intervals (100 frames per replica). Binding free energies were computed using Prime (release 2025-4, Schrödinger, LLC, New York, NY, USA, 2025) with the VSGB 2.1 solvation model and the OPLS4 force field [45,94]. For each complex, the mean binding free energy (ΔG_bind_) and standard deviation were calculated across all frames. This trajectory-based MM-GBSA analysis was used exclusively for comparative evaluation of binding stability.

Visualizations of the simulated complexes were generated using the PyMOL Molecular Graphics System, Version 3.1.6.1 (Schrödinger, LLC, New York, NY, USA, 2025).

## 5. Conclusions

In this study, an integrated computational workflow combining DFT geometry optimization, validated docking, molecular dynamics simulations, and MM-GBSA analysis provided detailed atomistic insight into the binding behavior of Fc-containing BCR-ABL1 inhibitor analogues. The approach demonstrated strong discriminatory power between active and inactive compounds, highlighting its utility for structure-based design.

The active analogues, particularly compounds **9** and **15a**, exhibited stable binding within the ATP-binding pocket despite lacking the canonical hinge interaction with Met318. Instead, binding was driven by alternative interaction networks centered on the DFG motif (Asp381) and reinforced by cation–π interactions with Lys271, supporting stabilization of the inactive kinase conformation. Notably, compound **9** displayed a more constrained and reproducible binding mode, accompanied by reduced P-loop flexibility and persistent Tyr253 engagement, supporting a potential contribution of P-loop stabilization to a trapping-like inhibition mechanism.

In contrast, the inactive analogue **15e** failed to establish a stable and well-defined binding mode, instead showing increased conformational heterogeneity, reduced burial within the ATP-binding site, and loss of key anchoring interactions, including those involving Lys271 and the P-loop region. This behavior is consistent with its diminished predicted binding affinity and reduced interaction persistence across simulations.

Comparative analyses further indicated that differences in conformational flexibility and limited Thr315 engagement may influence sensitivity to gatekeeper mutations, while persistent interactions with Tyr253 in the P-loop point to a potential strategy for mitigating resistance associated with P-loop variants. Importantly, these computational findings provide a structural rationale for the previously reported cytotoxic activity of the investigated compounds, linking their experimentally observed effects to specific binding interactions and conformational features within the BCR-ABL1 active site.

Overall, these findings support the feasibility of achieving effective ABL1 inhibition without classical hinge binding and highlight key structural determinants—including interaction network reorganization, ligand rigidity, and P-loop dynamics—that may guide further optimization of Fc-containing kinase inhibitors. Future studies incorporating mutant kinase systems and experimental validation will be required to confirm these observations and assess their translational relevance.

## Figures and Tables

**Table 1:** Lipinski’s Rule of Five (Ro5) descriptors for the investigated ferrocene-containing compounds, including molecular weight (MW), hydrogen bond donor (HBD) and hydrogen bond acceptor (HBA) counts, and DFT-derived logP values.

Compound	MW (Da)	HBA	HBD	logP	Ro5 Violations
**3**	369.09	3	1	5.73	1 (logP)
**9**	488.13	4	2	6.30	1 (logP)
**15a**	488.13	4	2	6.20	1 (logP)
**15b**	574.17	6	2	5.37	2 (MW, logP)
**15c**	613.18	6	3	5.55	2 (MW, logP)
**15d**	564.16	7	3	4.13	1 (MW)
**15e**	590.16	7	3	4.54	1 (MW)

**Table 2:** Enrichment analysis of the retrospective virtual screening validation. Recovery of known BCR-ABL1 actives at selected screening thresholds using the Glide SP docking protocol.

Dataset Screened (%)	Actives Recovered (*n*)	Actives Recovered (%)	Enrichment Factor
1	18	35.3	35.0
2	18	35.3	18.0
5	19	37.3	7.4
10	22	43.1	4.3
20	26	51.0	2.5

**Table 3:** In vitro cytotoxicity, expressed as IC_50_ values (μM), and corresponding Glide docking scores (kcal/mol) against BCR-ABL1. IC_50_ values for the Fc-containing compounds and imatinib were taken from our previously reported experimental study [46]. IC_50_ values for nilotinib in K562 and LAMA-84 cells were obtained from a separate study in which cytotoxicity was evaluated using a tetrazolium-based MTT assay after 48 h of exposure [54]. Nilotinib was not evaluated against AR-230 and BV-173 cells in that study; therefore, these values are reported as not determined (ND).

Compound	K-562	LAMA-84	AR-230	BV-173	Glide Score
**3**	66.5 ± 4.3	118.6 ± 7.4	>400	70.1 ± 6.4	−6.49
**9**	28.9 ± 4.1	0.9 ± 0.2	5.7 ± 0.9	48.0 ± 7.2	−9.18
**15a**	0.8 ± 0.2	0.4 ± 0.1	30.3 ± 5.5	10.1 ± 2.7	−9.27
**15b**	11.7 ± 1.2	72.9 ± 3.3	123.7 ± 12.2	64.6 ± 4.1	−4.60
**15c**	>200	199 ± 10.1	>200	67.2 ± 5.1	−5.25
**15d**	56.3 ± 4.7	142.5 ± 13.4	96.8 ± 8.3	>400	−5.36
**15e**	74.3 ± 6.2	88.7 ± 5.4	71.4 ± 7.8	36.7 ± 4.3	−5.70
imatinib	45.5 ± 5.0	0.5 ± 0.05	4.7 ± 1.0	22.8 ± 4.8	−12.62
nilotinib	0.10 ± 0.03 *	0.03 ± 0.01 *	ND	ND	−13.85

ND, not determined. * Nilotinib IC_50_ values for K562 and LAMA-84 cells were taken from an independent 48 h MTT-based cytotoxicity study [54]. Corresponding values for AR-230 and BV-173 cells were unavailable and are therefore reported as ND. GlideScore values were generated in the present study.

**Table 4:** Pose-based MM-GBSA ΔG_bind_ estimates for Fc-containing analogues and the reference inhibitors imatinib and nilotinib. All values are reported in kcal/mol.

Compound	3	9	15a	15b	15c	15d	15e	Imatinib	Nilotinib
**ΔG_bind_**	8.86	−22.92	−14.07	1.66	−4.55	−2.96	−7.34	−97.15	−99.67

**Table 5:** Mean P-loop backbone (Cα) RMSD values (Å) obtained after least-squares superposition on the full kinase-domain Cα atoms. The corresponding results using structured-core alignment are provided in Supplementary Table S3.

Compound	Replica 1	Replica 2	Replica 3	Mean Across Replicas (Å)	SD (Å)
imatinib	1.77	2.49	2.16	2.14	0.36
nilotinib	2.91	2.14	1.84	2.30	0.55
**9**	2.29	2.09	1.83	2.07	0.23
**15a**	2.07	3.08	2.62	2.59	0.51
**15e**	3.94	2.39	2.22	2.85	0.95

**Table 6:** Mean per-residue backbone Cα RMSF values (Å) for the P-loop (residues 247–255), calculated after least-squares superposition of the full kinase-domain Cα atom set. The corresponding analysis based on structured-core alignment is provided in Table S4 (Supplementary Materials).

Compound	Replica 1	Replica 2	Replica 3	Mean Across Replicas (Å)	SD (Å)
imatinib	1.308	1.288	1.195	1.263	0.061
nilotinib	1.408	1.398	1.151	1.319	0.145
**9**	1.178	1.497	1.248	1.308	0.168
**15a**	1.536	1.602	1.408	1.515	0.099
**15e**	2.435	1.725	1.514	1.891	0.483

## Data Availability

The data presented in this study is available on request from the corresponding author.

## Supplementary Materials

The following supporting information can be downloaded at: https://www.mdpi.com/article/10.3390/molecules31122156/s1, Figures S1–S17: Figure S1: Three-dimensional structures of the studied Fc-containing compounds optimized at the B3LYP/def2-TZVP level of theory with D3(BJ) dispersion correction and SMD (water) implicit solvation model in ORCA 6.0.1. Molecular visualizations were generated in PyMOL. Carbon atoms are shown in grey, hydrogen in white, nitrogen in blue, oxygen in red, iron in orange; Figure S2: Docking poses of compounds (a) **3** (green), (b) **15b** (yellow), and **15c** (pink), and (c) **15d** (blue), and **15e** (orange), superimposed with the reference ligand imatinib (red); Figure S3: Interaction fingerprint comparison of BCR-ABL1–ligand complexes. Interaction frequency per residue for imatinib, nilotinib, and the studied compounds (**9**, **15a**–**e**) within the BCR-ABL1 kinase binding site. The lower panel shows the occurrence of residue–ligand contacts across complexes, while the upper panel summarizes the overall interaction frequency per residue. Residues are grouped and color-coded by structural motifs: P-loop (red), αC-helix (green), hinge region (cyan), gatekeeper/T315I position (yellow), HRD motif (orange), DFG motif (magenta), A-loop (light blue), N-lobe (light gray), and C-lobe (dark gray). Conserved interaction hotspots are observed in the hinge (notably Phe317 and Met318), gatekeeper region (Thr315), and DFG motif (Asp381–Phe382), supporting a binding mode consistent with ATP-competitive inhibition; Figure S4: Protein backbone Cα RMSD over 500 ns molecular dynamics simulations for (A) imatinib, (B) nilotinib, (C) compound **9**, (D) compound **15a**, (E) compound **15e**. Three independent replicas are shown per compound (first replica—yellow; second replica—pink; third replica—blue). RMSD values were calculated for Cα atoms following least-squares fitting of the protein backbone; Figure S5: Ligand RMSD relative to the protein backbone over 500 ns molecular dynamics simulations for (A) imatinib, (B) nilotinib, (C) compound **9**, (D) compound **15a**, (E) compound **15e**. Three independent replicas are shown per compound (first replica—yellow; second replica—pink; third replica—blue). RMSD values are calculated for all non-hydrogen ligand atoms following least-squares fitting of the protein backbone; Figure S6: Mean P-loop backbone (Cα) RMSD as a function of simulation time during 500 ns molecular dynamics simulations of BCR-ABL1 in complex with imatinib, nilotinib, compound **9**, compound **15a**, and compound **15e**. Each profile represents the average of three independent replicas for the corresponding complex; Figure S7: Equivalent P-loop backbone (Cα) RMSD time profiles obtained using the structured-core alignment, in which the flexible terminal regions (residues < 235 and >500) and the activation loop (residues 381–401) were excluded from the superposition. The close agreement with Figure S6 confirms that the reported P-loop flexibility is robust to the choice of alignment reference; Figure S8: Mean per-residue P-loop backbone (Cα) RMSF from 500 ns molecular dynamics simulations of BCR-ABL1 in complex with imatinib, nilotinib, compound **9**, compound **15a**, and compound **15e**, calculated after superposition on all protein Cα atoms. Each profile represents the average across three independent replicas for the corresponding complex; shaded regions indicate the standard deviation between replicas; Figure S9: Mean per-residue P-loop backbone (Cα) RMSF from 500 ns molecular dynamics simulations of BCR-ABL1 in complex with imatinib, nilotinib, compound **9**, compound **15a**, and compound **15e**, calculated after superposition on the structured-core Cα atoms (residues 235–500), excluding the activation loop (residues 381–401). Each profile represents the average across three independent replicas for the corresponding complex; shaded regions indicate the standard deviation between replicas; Figure S10: Root-mean-square fluctuation (RMSF) profiles of BCR-ABL1 Cα atoms during 500 ns molecular dynamics simulations of the complexes with imatinib, nilotinib, compound **9**, compound **15a**, and compound **15e**. RMSF values represent mean fluctuations averaged over three independent replicas; Figure S11: PCA projections and free energy landscapes for the reference inhibitors imatinib and nilotinib. PCA projections were obtained in the common PC1/PC2 space of each system after alignment on the structured kinase-core Cα atoms. Free energy landscapes were reconstructed from the pooled PC1/PC2 projections of the three independent replicas, and free energy values are shown relative to the most populated bin; Figure S12: PCA projections and free energy landscapes for the ferrocene-containing analogs **9**, **15a**, and **15e**. PCA projections were obtained in the common PC1/PC2 space of each system after alignment on the structured kinase-core Cα atoms. Compound **15e** was included as an inactive negative-control analog. Free energy landscapes were reconstructed from the pooled PC1/PC2 projections of the three independent replicas, and free energy values are shown relative to the most populated bin; Figure S13: Superposition of the initial binding pose (frame 0 of the molecular dynamics simulation, red) and the medoid structures of the most populated ligand clusters identified from the three independent replicas—replica 1 (blue), replica 2 (green), and replica 3 (bright orange)—for (a) imatinib, (b) nilotinib, and (c) compound **15e**. (a) For imatinib, the dominant clusters accounted for 35.7% (178.3 ns, medoid at 160.7 ns), 80.7% (403.5 ns, medoid at 235.8 ns), and 27.9% (139.7 ns, medoid at 125.1 ns) of the respective trajectories. The superposition illustrates the dominant binding geometries sampled during the simulations relative to the starting pose. (b) For nilotinib, the dominant clusters accounted for 50.0% (250.2 ns, medoid at 429.7 ns), 34.4% (172.1 ns, medoid at 464.5 ns), and 49.4% (247.0 ns, medoid at 423.3 ns) of the respective trajectories; in replica 3, a second, near-equally populated cluster (44.6%) corresponded to a closely related sub-state of the same binding mode. (c) For compound **15e**, the most populated clusters accounted for 26.6% (133.1 ns, medoid at 257.2 ns) and 37.2% (185.9 ns, medoid at 34.8 ns) in replicas 1 and 2, respectively, while replica 3 showed no dominant state, with its three leading clusters nearly equally populated (16.3%, 16.1% and 15.5%); the orange structure in panel (c) therefore represents the largest cluster only. The superpositions illustrate the dominant binding geometries sampled during the simulations relative to the starting pose, with the markedly more dispersed ensemble of compound **15e** reflecting the absence of a persistent, well-defined binding mode; Figure S14: Radius of gyration (rGyr) of the ligand over 500 ns molecular dynamics simulations for (A) imatinib, (B) nilotinib, (C) compound **9**, (D) compound **15a**, and (E) compound **15e**. The rGyr values are shown as a function of simulation time to monitor the overall compactness and conformational stability of the ligand throughout the trajectory; Figure S15: Molecular surface area (MolSA) of the ligand over 500 ns molecular dynamics simulations for (A) imatinib, (B) nilotinib, (C) compound **9**, (D) compound **15a**, and (E) compound **15e**. MolSA values were calculated for the ligand and plotted as a function of time to monitor changes in molecular surface throughout the simulations; Figure S16: Solvent-accessible surface area (SASA) of the ligand over 500 ns molecular dynamics simulations for (A) imatinib, (B) nilotinib, (C) compound **9**, (D) compound **15a**, and (E) compound **15e**. SASA values were calculated for the ligand and plotted as a function of time to assess the degree of ligand exposure to the solvent during the simulations; Figure S17: Polar surface area (PSA) of the ligand over 500 ns molecular dynamics simulations for (A) imatinib, (B) nilotinib, (C) compound **9**, (D) compound **15a**, and (E) compound **15e**. PSA values were calculated for the ligand and plotted over time to monitor changes in the exposed polar surface area during the simulations; Tables S1–S9: Table S1: Root-mean-square deviation (RMSD) values obtained from the structural alignment of DFT-optimized Fc cores with the crystallographic reference extracted from the 5MYQ structure; Table S2: MM-GBSA energy decomposition (kcal/mol) of the docked poses for imatinib, nilotinib, and the ferrocene-containing analogs; Table S3: Mean P-loop backbone (Cα) RMSD values (Å) from three independent 500 ns molecular dynamics replicas of BCR-ABL1 in complex with imatinib, nilotinib, compound 9, compound 15a, and compound 15e, obtained using the structured-core alignment protocol. Each trajectory frame was superposed by least-squares fitting on a structured-core Cα subset excluding the flexible terminal regions (residues < 235 and >500) and the activation loop (residues 381–401). Reported values include per-replica means, overall means across replicas, and standard deviations (SD); Table S4: Mean P-loop backbone (Cα) RMSF values (Å), averaged over residues 247–255 and calculated after least-squares superposition on the structured-core Cα subset (kinase-domain Cα atoms excluding flexible N- and C-terminal segments (residues < 235 and >500) and the activation loop (residues 381–401); Table S5: Geometric validation of the ferrocene moiety during the molecular dynamics simulations. Mean values ± standard deviations for Fe–C distances, Fe–Cp centroid distances, Cp–Cp centroid distance, Cp-ring interplanar angle, ferrocene-core RMSD relative to the DFT-optimised reference structure, and Cp-ring planarity calculated over three independent 500 ns MD replicas; Table S6: Summary of PCA variance, cosine content, and FEL minima for the analysed systems. Cosine content values are reported as mean ± standard deviation across three independent replicas. FEL minimum coordinates correspond to the global minimum in the reconstructed PC1/PC2 free-energy landscape; Table S7: Quantitative interaction fingerprint analysis derived from molecular dynamics trajectories of imatinib and the Fc-containing analogs. The table summarizes interaction frequencies across three independent MD replicas for each ligand, including hydrogen bonds, hydrophobic contacts, ionic interactions, aromatic interactions, and water bridges. Percentage values represent the proportion of analyzed trajectory frames in which a given interaction is present, calculated over 2500 frames per replica, and thus reflect the occupancy of each interaction over the sampled simulation time; Table S8: Quantitative interaction fingerprint analysis derived from molecular dynamics trajectories of nilotinib and compound 15e. The table summarizes interaction frequencies across three independent MD replicas for each ligand, including hydrogen bonds, hydrophobic contacts, ionic interactions, aromatic interactions, and water bridges. Percentage values represent the proportion of analyzed trajectory frames in which a given interaction is present, calculated over 2500 frames per replica, and thus reflect the occupancy of each interaction over the sampled simulation time; Table S9: MM-GBSA energy decomposition analysis for imatinib, nilotinib, compound 9, 15a, and 15e based on the last 100 ns of the molecular dynamics simulations (400–500 ns). Values are presented as mean ± SD across trajectory frames for each independent replica, together with the overall mean ± SD across the three replicas. The table reports the total binding free energy estimate (ΔG_bind_) and its individual components, including the Coulombic component, covalent, van der Waals, Lipophilic, solvation (GB), hydrogen-bonding, and packing terms, all expressed in kcal/mol.

## Abbreviations

The following abbreviations are used in this manuscript:

ABL1	Abelson murine leukemia viral oncogene homolog 1
ATP	Adenosine triphosphate
AUC	Area under the curve
B3LYP	Becke, 3-parameter, Lee–Yang–Parr (exchange-correlation functional)
BCR-ABL1	BCR-ABL fusion gene/protein
BEDROC	Boltzmann-enhanced discrimination of receiver operating characteristic
CML	Chronic myeloid leukemia
Cp	Cyclopentadienyl
D3BJ	Grimme’s dispersion correction with Becke–Johnson damping
def2-TZVP	Def2 triple-zeta valence polarization (basis set)
DFG	Asp-Phe-Gly motif
DFT	Density functional theory
DUD-E	Directory of Useful Decoys, Enhanced
EF	Enrichment factor
FDA	U.S. Food and Drug Administration
Fc	Ferrocene
GAFF2	General AMBER Force Field 2
GFN-FF	Geometries, Frequencies, and Non-covalent interactions Force Field
GenAI	Generative artificial intelligence tools
Qt2	His-Arg-Asp motif
IC_50_	Half maximal inhibitory concentration
IFP	Interaction fingerprint
MD	Molecular dynamics
MDR1	Multidrug resistance protein 1
MM-GBSA	Molecular mechanics–generalized Born surface area
MolSA	Molecular surface area
MTT	3-(4,5-dimethylthiazol-2-yl)-2,5-diphenyltetrazolium bromide (cytotoxicity assay)
NaCl	Sodium chloride
NPT	Isothermal-isobaric ensemble
NVT	Canonical ensemble
OCT1	Organic cation transporter 1
OPLS4	Optimized potentials for liquid simulations 4
PDB	Protein Data Bank
P-gp	P-glycoprotein
Ph	Philadelphia chromosome
PI3K/AKT	Phosphoinositide 3-kinase/protein kinase B
PME	Particle mesh Ewald method
PSA	Polar surface area
QM	Quantum mechanical
RAS/MAPK	Rat sarcoma/mitogen-activated protein kinase
rGyr	Radius of gyration
RIJCOSX	Resolution of identity with chain-of-spheres exchange approximation
RMSD	Root mean square deviation
RMSF	Root mean square fluctuation
ROC	Receiver operating characteristic
SAR	Structure–activity relationship
SASA	Solvent-accessible surface area
SD	Standard deviation
SID	Simulation Interaction Diagram
SMD	Solvation model based on density
SMILES	Simplified molecular-input line-entry system
SP	Standard precision (Glide docking protocol)
STAT5	Signal transducer and activator of transcription 5
T315I	Threonine-to-isoleucine substitution at position 315
TIP3P	Transferable intermolecular potential with 3 points
TKI	Tyrosine kinase inhibitor
vdW	Van der Waals
VSGB	Variable-dielectric generalized Born solvation model
ΔG_bind_	Binding free energy
