# Supplementary material for: Non-Classical Binding Mechanisms of Ferrocene-Modified Imatinib and Nilotinib Analogues in BCR-ABL1 Kinase Revealed by Computational Analysis
Source: Molecules. 2026 Jun 18;31(12):2156. doi: 10.3390/molecules31122156 (PMC13304655; doi:10.3390/molecules31122156)
Supplement: Supplementary file 1 [file molecules-31-02156-s001.zip › molecules-4333026-supplementary.pdf]

Supplementary file

# Non-Classical Binding Mechanisms of Ferrocene-Modified Imatinib and Nilotinib Analogues in BCR-ABL1 Kinase Revealed by Computational Analysis

**Rostislava Angelova[1,2], Georgi Stavrakov[1,3,4], Georgi Momekov[2], Danislav S. Spassov[1,4], and Mariyana Atanasova[1,4]***

[1] Chemistry Department, Faculty of Pharmacy, Medical University of Sofia, 2 Dunav Str., 1000 Sofia, Bulgaria; 105645@students.mu-sofia.bg (R.A.); stavrakov@pharmfac.mu-sofia.bg (G.S.);

[2] Department of Pharmacology, Pharmacotherapy and Toxicology, Faculty of Pharmacy, Medical University of Sofia, 2 Dunav Str., 1000 Sofia, Bulgaria gmomekov@pharmfac.mu-sofia.bg (G.M)

[3] Institute of Organic Chemistry with Centre of Phytochemistry, Bulgarian Academy of Sciences, Acad. G. Bonchev Str. 9, 1113 Sofia, Bulgaria;

[4] Centre of Excellence in Informatics and Information and Communication Technologies, Bulgarian Academy of Sciences, Akad. Georgi Bonchev Str., Block 2 and 25A, 1113 Sofia, Bulgaria

* Correspondence: matanasova@pharmfac.mu-sofia.bg (M.A.)

## S1. RMSD comparison of DFT-optimized Fc fragments with the crystallographic Fc reference from 5MYQ

**Table S1.** Root-mean-square deviation (RMSD) values obtained from the structural alignment of DFT-optimized Fc cores with the crystallographic reference extracted from the 5MYQ structure.

| Compound | RMSD (Å) |
|---|---|
| **3** | 0.022 |
| **9** | 0.024 |
| **15a** | 0.023 |
| **15b** | 0.421 |
| **15c** | 0.414 |
| **15d** | 0.420 |
| **15e** | 0.412 |

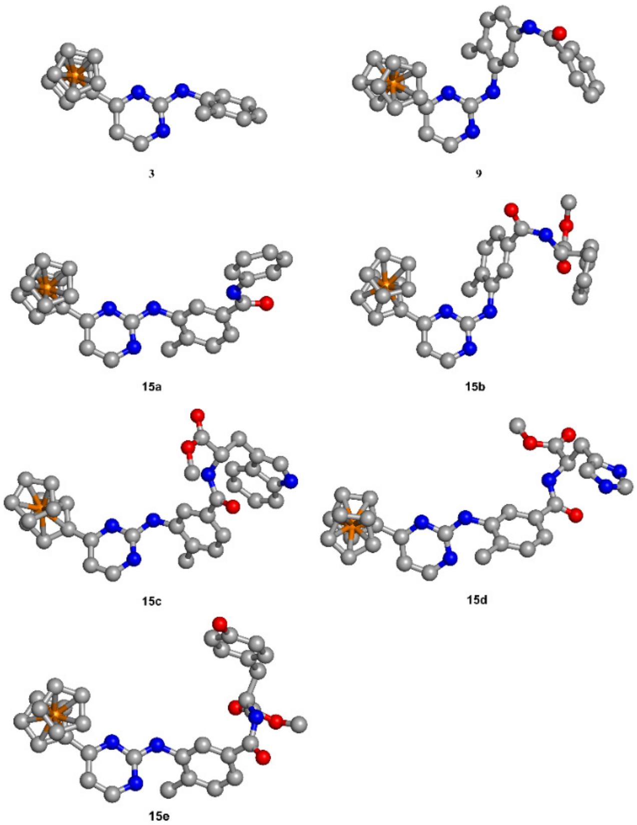


**Figure S1.** Three-dimensional structures of the studied Fc-containing compounds optimized at the B3LYP/def2-TZVP level of theory with D3(BJ) dispersion correction and SMD (water) implicit solvation model in ORCA 6.0.1. Molecular visualizations were generated in PyMOL. Carbon atoms are shown in grey, hydrogen in white, nitrogen in blue, oxygen in red, iron in orange.

## Brief analysis of docking poses of lower-ranked compounds

Compound **3** lacked the aromatic amide anchor. resulting in loss of hinge alignment and incomplete interaction networks (Figure S2). Compounds **15b–e**, which differ only in amino acid ester residues, consistently adopted peripheral binding orientations that prevented key aromatic and backbone interactions. Despite modest cytotoxicity observed for compound **15b**, increased flexibility and polarity of amino acid–derived linkers likely reduce hydrophobic complementarity within the ATP-binding pocket. For inactive compounds, the selected pose represents the most plausible non-artefactual conformation and is intended for structural comparison rather than as evidence of productive binding.

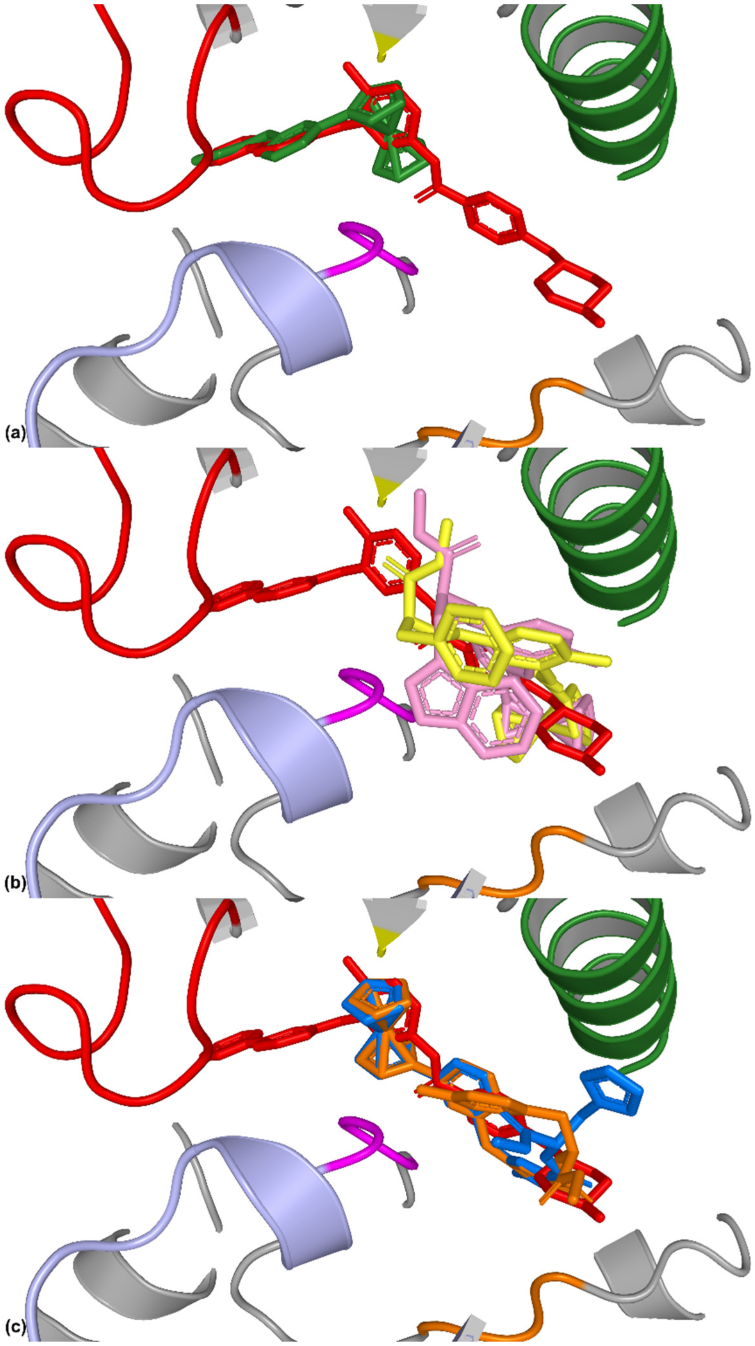


**Figure S2**. Docking poses of compounds (a) **3 (green)**, (b) **15b** (yellow), and **15c** (pink), and (c) **15d** (blue), and **15e** (orange), superimposed with the reference ligand imatinib (red).

**Interaction fingerprint comparison of BCR-ABL1 –ligand complexes**

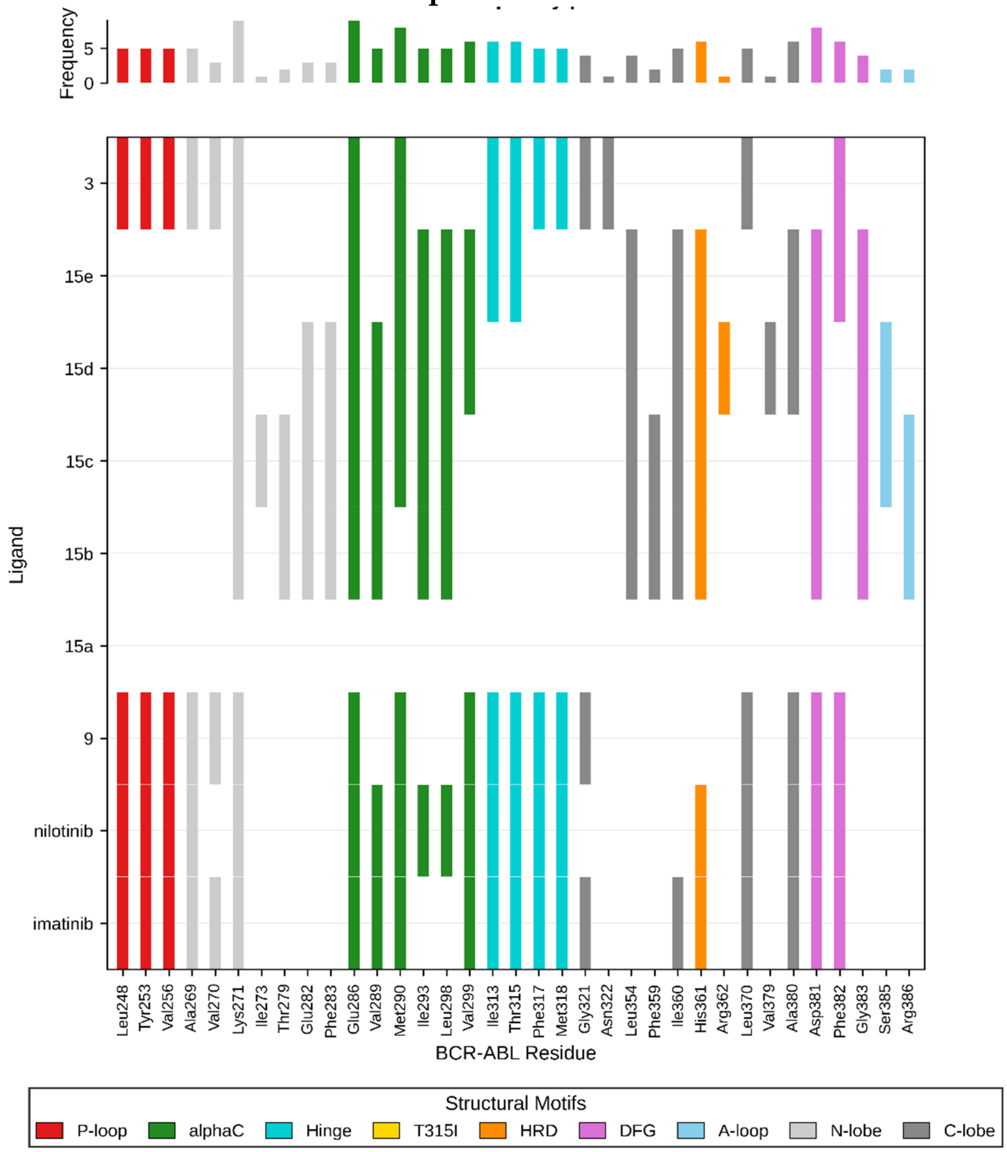


**Figure S3.** Interaction fingerprint comparison of BCR-ABL1–ligand complexes. Interaction frequency per residue for imatinib, nilotinib, and the studied compounds (**9**, **15a–e**) within the BCR-ABL1 kinase binding site. The lower panel shows the occurrence of residue–ligand contacts across complexes. while the upper panel summarizes the overall interaction frequency per residue. Residues are grouped and color-coded by structural motifs: P-loop (red). $\alpha$C-helix (green). hinge region (cyan). gatekeeper/T315I position (yellow). HRD motif (orange). DFG motif (magenta). A-loop (light blue). N-lobe (light gray). and C-lobe (dark gray). Conserved interaction hotspots are observed in the hinge (notably Phe317 and Met318). gatekeeper region (Thr315). and DFG motif (Asp381–Phe382). supporting a binding mode consistent with ATP-competitive inhibition.

## MM-GBSA energy decomposition of docked poses

**Table S2.** MM-GBSA energy decomposition (kcal/mol) of the docked poses for imatinib, nilotinib, and the ferrocene-containing analogs.

| Compound | $\Delta G_{bind}$ | $\Delta G_{Coulomb}$ | $\Delta G_{Covalent}$ | $\Delta G_{vdW}$ | $\Delta G_{Lipo}$ | $\Delta G_{Solv}$ | $\Delta G_{Hbond}$ | $\Delta G_{Packing}$ |
|---|---|---|---|---|---|---|---|---|
| **3** | 8.86 | -17.49 | 10.62 | -31.32 | -36.97 | 86.41 | -0.45 | -1.90 |
| **9** | -22.92 | -21.61 | 5.98 | -52.54 | -45.45 | 94.01 | -1.26 | -1.92 |
| **15a** | -14.07 | -24.22 | 15.96 | -51.27 | -45.61 | 94.72 | -1.52 | -2.11 |
| **15b** | 1.66 | 11.08 | -1.54 | -57.72 | -28.87 | 79.15 | 1.25 | -1.69 |
| **15c** | -4.55 | 6.29 | -1.62 | -58.53 | -29.45 | 79.07 | 0.22 | -0.53 |
| **15d** | -2.96 | -23.00 | -5.73 | -52.09 | -24.66 | 105.71 | -1.67 | -1.51 |
| **15e** | -7.34 | -20.17 | -0.36 | -59.90 | -32.25 | 108.72 | -1.34 | -2.04 |
| imatinib | -97.15 | -94.61 | 9.67 | -70.28 | -41.09 | 105.78 | -1.82 | -4.81 |
| nilotinib | -99.67 | -24.04 | 3.39 | -72.68 | -36.04 | 36.39 | -1.73 | -4.97 |

# Molecular Dynamics Trajectory Analyses

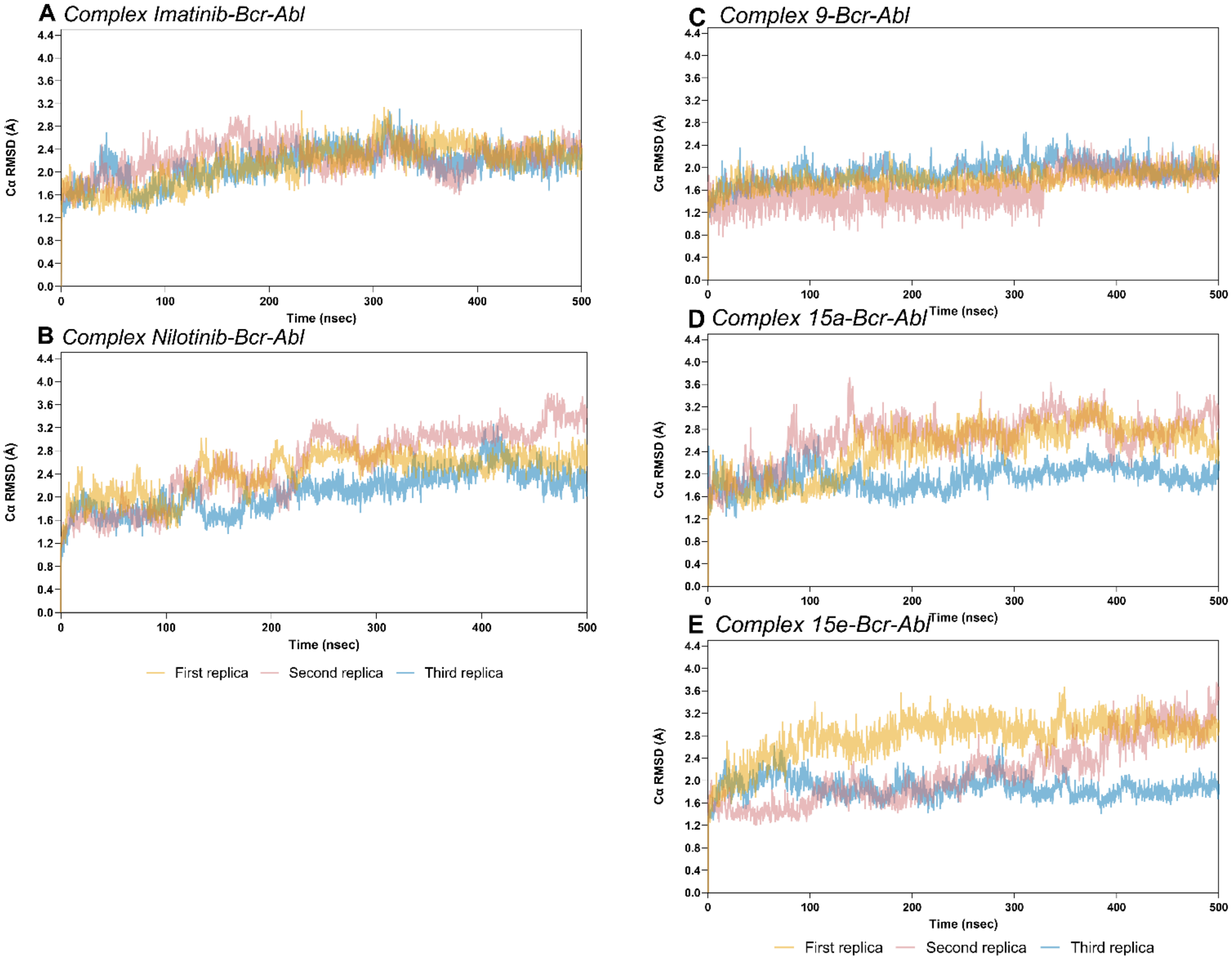


**Figure S4.** Protein backbone C$\alpha$ RMSD over 500 ns molecular dynamics simulations for (A) imatinib, (B) nilotinib, and (C) compound **9,** (D) compound **15a**, (E) compound **15e**. Three independent replicas are shown per compound (first replica — yellow; second replica — pink; third replica — blue). RMSD values were calculated for C$\alpha$ atoms following least-squares fitting of the protein backbone.

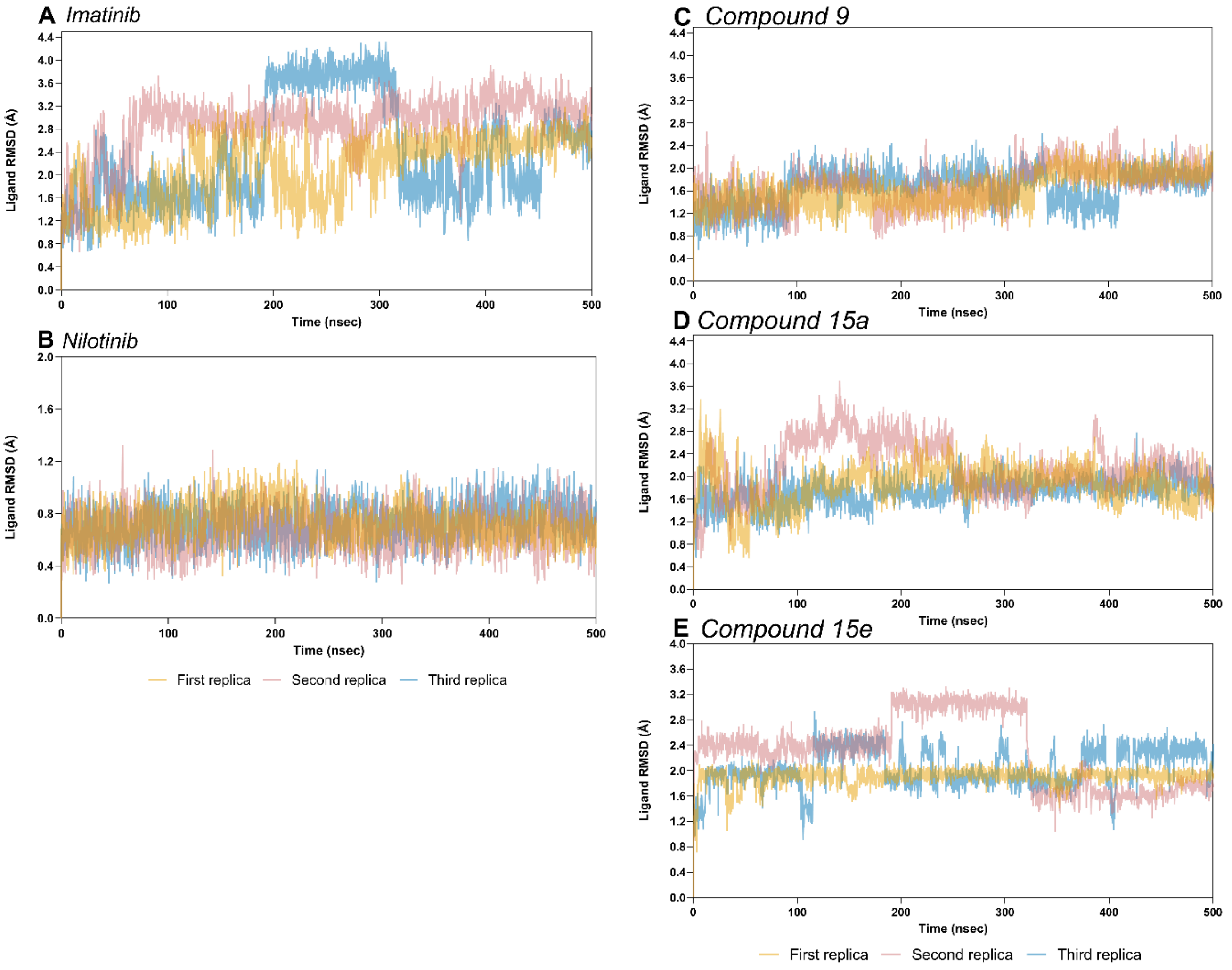


**Figure S5.** Ligand RMSD relative to the protein backbone over 500 ns molecular dynamics simulations for (A) imatinib, (B) nilotinib, and (C) compound **9,** (D) compound **15a**, (E) compound **15e**. Three independent replicas are shown per compound (first replica — yellow; second replica — pink; third replica — blue). RMSD values are calculated for all non-hydrogen ligand atoms following least-squares fitting of the protein backbone.

**Table S3.** Mean P-loop backbone (C$\alpha$) RMSD values (Å) from three independent 500 ns molecular dynamics replicas of BCR-ABL1 in complex with imatinib, nilotinib, compound **9**, compound **15a**, and compound **15e**, obtained using the structured-core alignment protocol. Each trajectory frame was superposed by least-squares fitting on a structured-core C$\alpha$ subset excluding the flexible terminal regions (residues <235 and >500) and the activation loop (residues 381–401). Reported values include per-replica means, overall means across replicas, and standard deviations (SD).

| Compound | Replica 1 | Replica 2 | Replica 3 | Mean across replicas (Å) | SD (Å) |
|---|---|---|---|---|---|
| imatinib | 1.88 | 2.64 | 2.34 | 2.28 | 0.38 |
| nilotinib | 2.98 | 1.90 | 1.96 | 2.28 | 0.61 |
| **9** | 2.64 | 2.28 | 2.11 | 2.34 | 0.27 |
| **15a** | 2.10 | 3.49 | 2.87 | 2.82 | 0.70 |
| **15e** | 4.17 | 2.46 | 2.29 | 2.97 | 1.04 |

The ranking and magnitudes are consistent with the full-C$\alpha$ results presented in Table 4 (differences <0.3 Å). confirming that the reported P-loop flexibility is not an artefact of including highly mobile regions in the superposition.

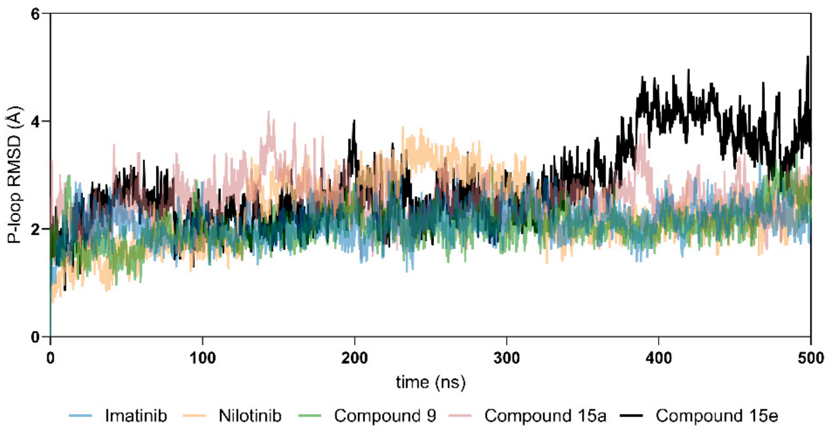


**Figure S6**. Mean P-loop backbone (C$\alpha$) RMSD as a function of simulation time during 500 ns molecular dynamics simulations of BCR-ABL1 in complex with imatinib, nilotinib, compound **9**, compound **15a**, and compound **15e**. Each profile represents the average of three independent replicas for the corresponding complex.

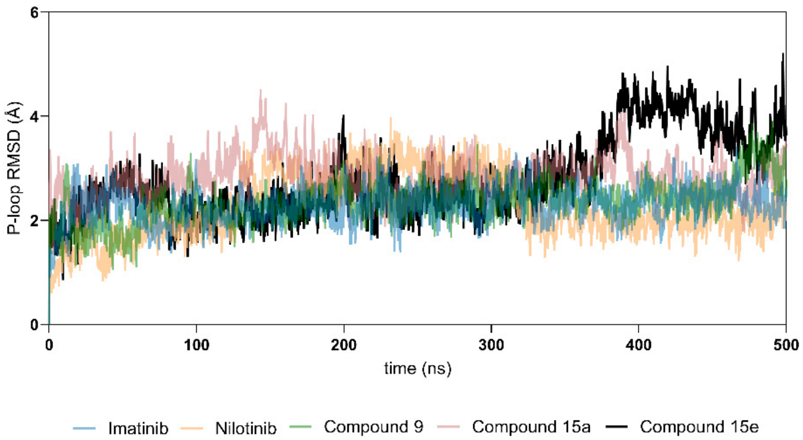


**Figure S7**. Equivalent P-loop backbone (C$\alpha$) RMSD time profiles obtained using the structured-core alignment, in which the flexible terminal regions (residues <235 and >500) and the activation loop (residues 381–401) were excluded from the superposition. The close agreement with Figure S6 confirms that the reported P-loop flexibility is robust to the choice of alignment reference.

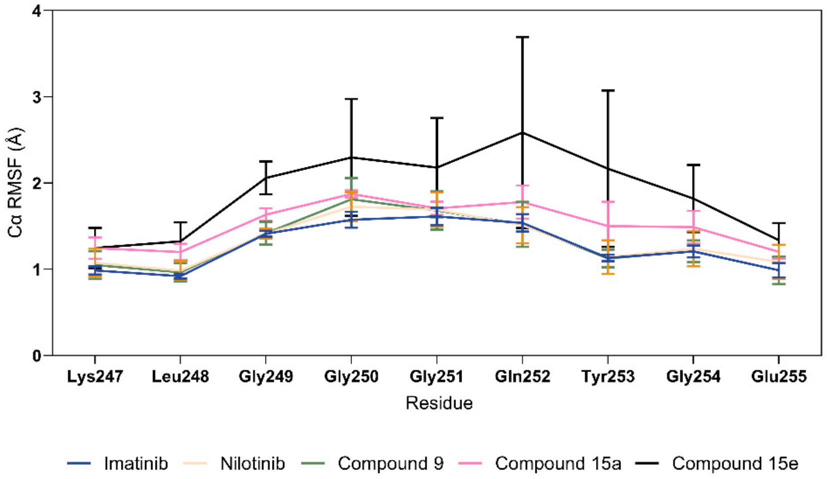


**Figure S8**. Mean per-residue P-loop backbone (C$\alpha$) RMSF from 500 ns molecular dynamics simulations of BCR-ABL1 in complex with imatinib, nilotinib, compound **9**, compound **15a**, and compound **15e**, calculated after superposition on all protein C$\alpha$ atoms. Each profile represents the average across three independent replicas for the corresponding complex; shaded regions indicate the standard deviation between replicas.

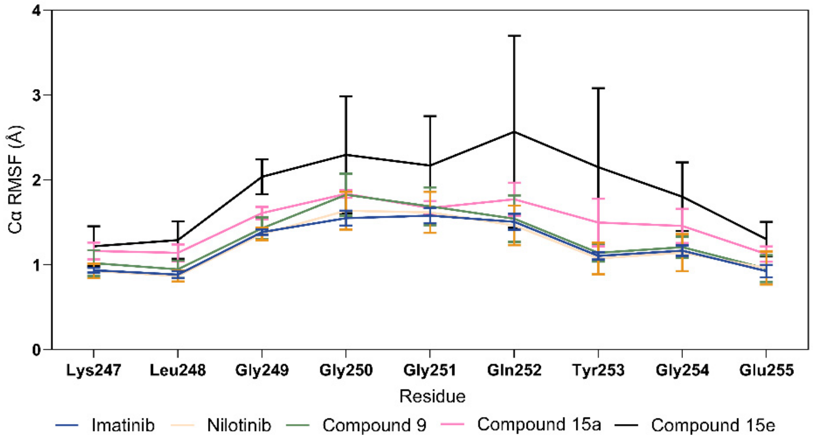


**Figure S9**. Mean per-residue P-loop backbone (C$\alpha$) RMSF from 500 ns molecular dynamics simulations of BCR-ABL1 in complex with imatinib, nilotinib, compound **9**, compound **15a**, and compound **15e**, calculated after superposition on the structured-core C$\alpha$ atoms (residues 235–500), excluding the activation loop (residues 381–401). Each profile represents the average across three independent replicas for the corresponding complex; shaded regions indicate the standard deviation between replicas.

**Table S4.** Mean P-loop backbone (C$\alpha$) RMSF values (Å), averaged over residues 247–255 and calculated after least-squares superposition on the structured-core C$\alpha$ subset (kinase-domain C$\alpha$ atoms excluding flexible N- and C-terminal segments (residues <235 and >500) and the activation loop (residues 381–401)).

| Compound | Replica 1 | Replica 2 | Replica 3 | Mean across replicas (Å) | SD (Å) |
|---|---|---|---|---|---|
| imatinib | 1.279 | 1.226 | 1.173 | 1.226 | 0.053 |
| nilotinib | 1.240 | 1.386 | 1.058 | 1.228 | 0.164 |
| **9** | 1.179 | 1.495 | 1.243 | 1.306 | 0.167 |
| **15a** | 1.500 | 1.565 | 1.357 | 1.474 | 0.107 |
| **15e** | 2.427 | 1.692 | 1.489 | 1.869 | 0.494 |

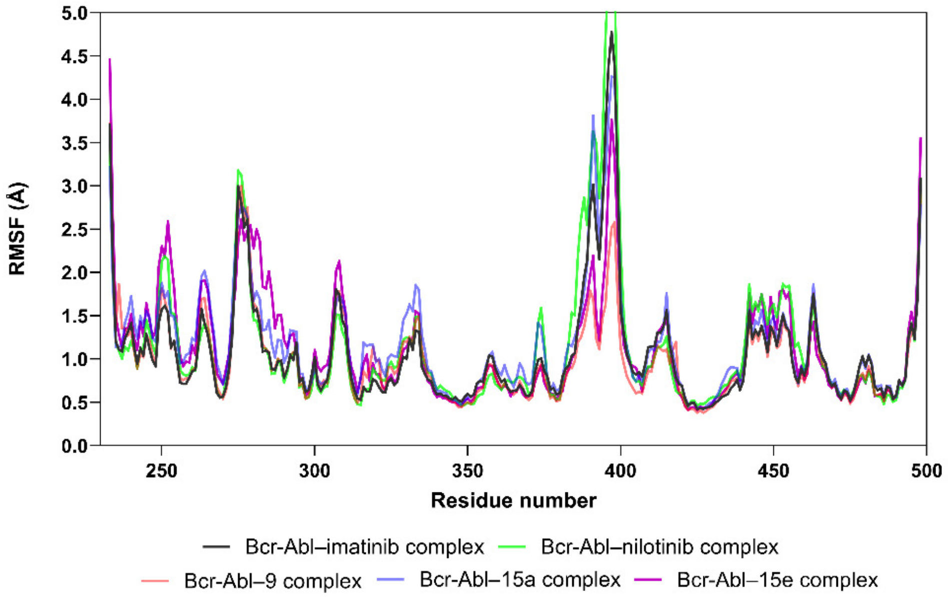


**Figure S10.** Root-mean-square fluctuation (RMSF) profiles of BCR-ABL1 C$\alpha$ atoms during 500 ns molecular dynamics simulations of the complexes with imatinib, nilotinib, compound **9**, compound **15a**, and compound **15e**. RMSF values represent mean fluctuations averaged over three independent replicas.

**Table S5.** Geometric validation of the ferrocene moiety during the molecular dynamics simulations. Mean values ± standard deviations for Fe–C distances, Fe–Cp centroid distances, Cp–Cp centroid distance, Cp-ring interplanar angle, ferrocene-core RMSD relative to the DFT-optimised reference structure, and Cp-ring planarity calculated over three independent 500 ns MD replicas.

| System | **Fe–C (Å)** | **Fe–Cp1 (Å)** | **Fe–Cp2 (Å)** | **Cp–Cp (Å)** | **Cp angle (°)** | **Fc RMSD vs DFT (Å)** | **Cp1 planarity (Å)** | **Cp2 planarity (Å)** |
|---|---|---|---|---|---|---|---|---|
| 9_rep1 | 2.1113 ± 0.0091 | 1.6775 ± 0.0172 | 1.6723 ± 0.0167 | 3.3492 ± 0.0229 | 2.5698 ± 1.3262 | 0.1078 ± 0.0189 | 0.0218 ± 0.0113 | 0.0243 ± 0.0128 |
| 9_rep2 | 2.1111 ± 0.0091 | 1.677 ± 0.0175 | 1.672 ± 0.0168 | 3.3483 ± 0.0226 | 2.6014 ± 1.3097 | 0.1084 ± 0.0191 | 0.0223 ± 0.0114 | 0.0252 ± 0.0128 |
| 9_rep3 | 2.1112 ± 0.0089 | 1.6767 ± 0.017 | 1.6728 ± 0.0171 | 3.3488 ± 0.0229 | 2.598 ± 1.3951 | 0.1066 ± 0.0185 | 0.0222 ± 0.0114 | 0.0249 ± 0.0128 |
| 15a_rep1 | 2.1082 ± 0.0091 | 1.6678 ± 0.0167 | 1.6724 ± 0.0167 | 3.3396 ± 0.0228 | 2.6183 ± 1.3488 | 0.1154 ± 0.0188 | 0.0375 ± 0.0153 | 0.0219 ± 0.0116 |
| 15a_rep2 | 2.1085 ± 0.0091 | 1.6681 ± 0.0171 | 1.673 ± 0.0169 | 3.3405 ± 0.0226 | 2.5553 ± 1.3107 | 0.1168 ± 0.0193 | 0.0381 ± 0.0151 | 0.0221 ± 0.0115 |
| 15a_rep3 | 2.1083 ± 0.0092 | 1.6686 ± 0.0172 | 1.6731 ± 0.0169 | 3.3411 ± 0.0227 | 2.5171 ± 1.2851 | 0.1117 ± 0.0182 | 0.038 ± 0.0152 | 0.0221 ± 0.0115 |
| 15e_rep1 | 2.1064 ± 0.0091 | 1.6674 ± 0.017 | 1.6647 ± 0.0167 | 3.3311 ± 0.0225 | 2.5895 ± 1.3285 | 0.0988 ± 0.0128 | 0.0209 ± 0.011 | 0.0251 ± 0.013 |
| 15e_rep2 | 2.1067 ± 0.0091 | 1.6674 ± 0.0173 | 1.6662 ± 0.0169 | 3.3324 ± 0.0232 | 2.7615 ± 1.3742 | 0.0985 ± 0.0123 | 0.0220 ± 0.0114 | 0.0250 ± 0.0125 |
| 15e_rep3 | 2.1066 ± 0.0090 | 1.6672 ± 0.0166 | 1.6657 ± 0.0171 | 3.3318 ± 0.0222 | 2.7249 ± 1.3614 | 0.0982 ± 0.0125 | 0.0212 ± 0.0113 | 0.0247 ± 0.0123 |
| Crystal Fc reference | 2.084 | 1.701 | 1.703 | 3.398 | 6.30 | n.a. | 0.010 | 0.005 |

n.a., not applicable. The Fc RMSD was calculated by comparing each MD trajectory frame with the corresponding compound-specific DFT-optimized pre-docking structure. Therefore, RMSD is not applicable to the crystallographic Fc reference, which was used only as an external benchmark for internal ferrocene geometric descriptors.

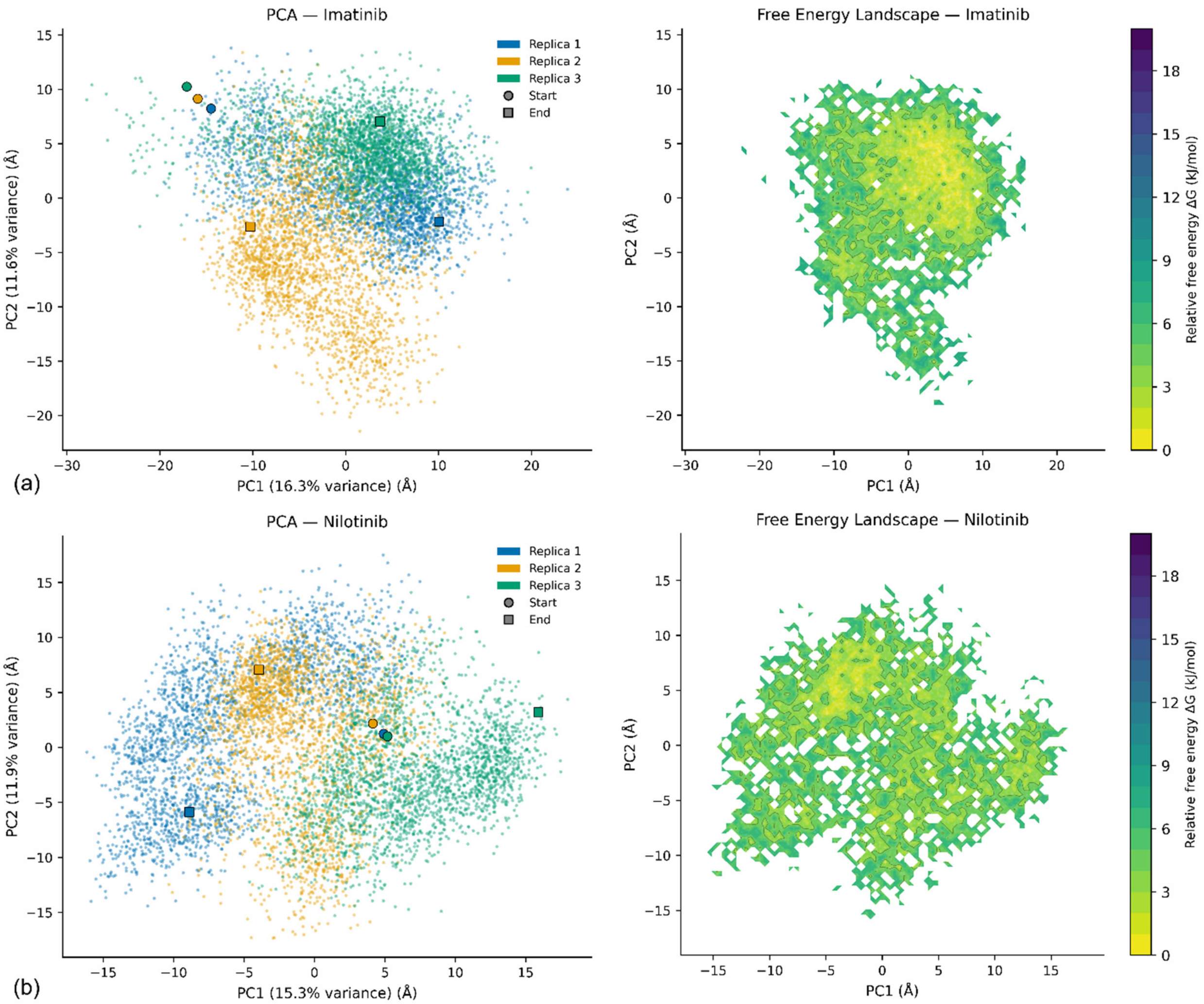


**Figure S11.** PCA projections and free energy landscapes for the reference inhibitors imatinib and nilotinib. PCA projections were obtained in the common PC1/PC2 space of each system after alignment on the structured kinase-core C$\alpha$ atoms. Free energy landscapes were reconstructed from the pooled PC1/PC2 projections of the three independent replicas, and free energy values are shown relative to the most populated bin.

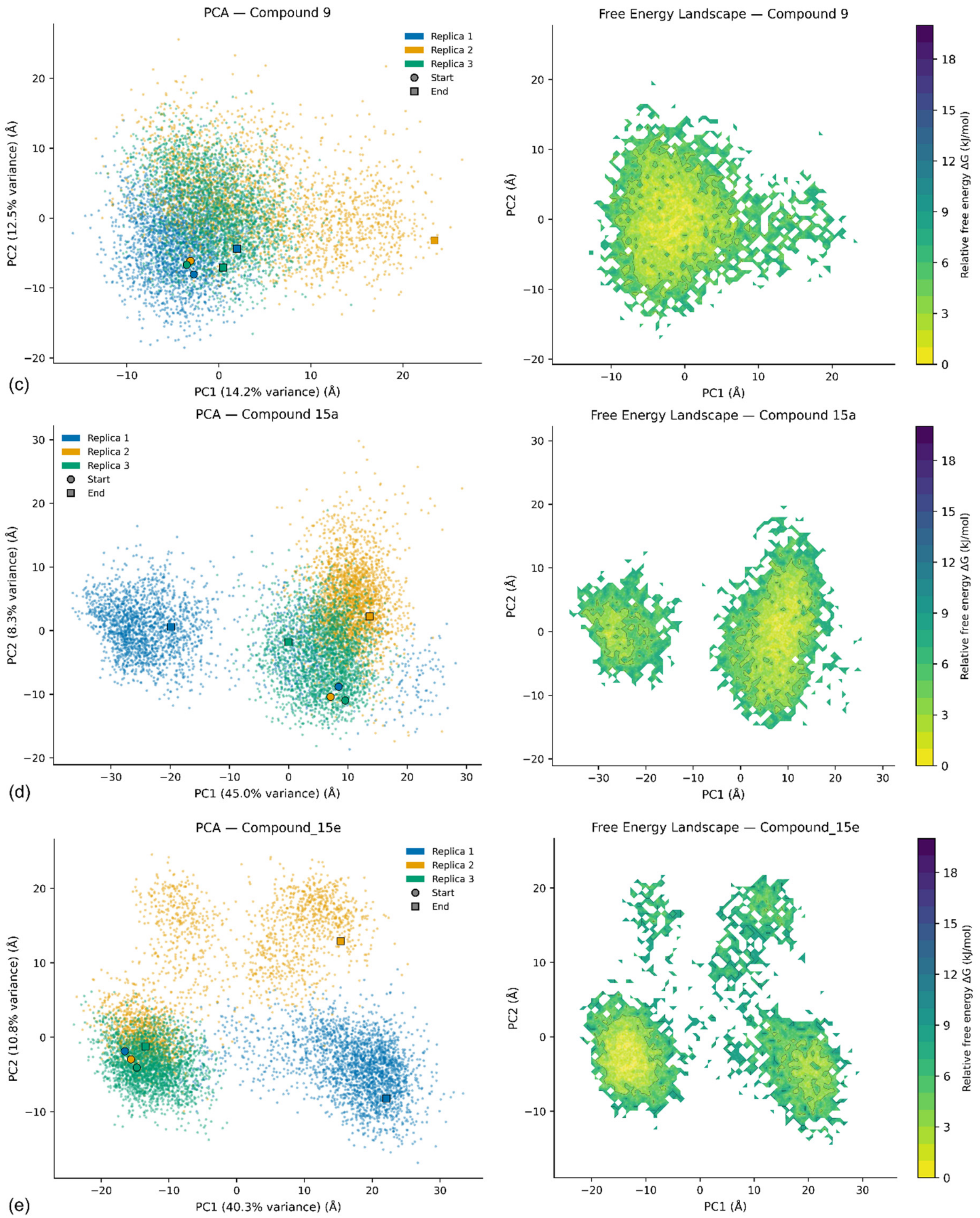


**Figure S12.** PCA projections and free energy landscapes for the ferrocene-containing analogs 9, 15a, and 15e. PCA projections were obtained in the common PC1/PC2 space of each system after alignment on the structured kinase-core C$\alpha$ atoms. Compound 15e was included as an inactive negative-control analog. Free energy landscapes were reconstructed from the pooled PC1/PC2 projections of the three independent replicas, and free energy values are shown relative to the most populated bin.

**Table S6.** Summary of PCA variance, cosine content, and FEL minima for the analysed systems. Cosine content values are reported as mean ± standard deviation across three independent replicas. FEL minimum coordinates correspond to the global minimum in the reconstructed PC1/PC2 free-energy landscape.

| PCA variance. cosine content and FEL minima for the analyzed systems | | | | | | | |
|---|---|---|---|---|---|---|---|
| System | **PC1 variance (%)** | **PC2 variance (%)** | **Total PC1+PC2 (%)** | **Cosine content PC1** | **Cosine content PC2** | **FEL min PC1** | **FEL min PC2** |
| Compound 9 | 14.2 | 12.5 | 26.7 | 0.14 ± 0.12 | 0.04 ± 0.06 | -1.33 | -2.27 |
| Compound 15a | 45.0 | 8.3 | 53.4 | 0.10 ± 0.16 | 0.09 ± 0.15 | 7.49 | 0.42 |
| Compound 15e | 40.3 | 10.8 | 51.1 | 0.25 ± 0.38 | 0.17 ± 0.19 | -12.19 | -3.66 |
| Imatinib | 16.3 | 11.6 | 28.0 | 0.31 ± 0.21 | 0.11 ± 0.14 | -0.39 | 0.81 |
| Nilotinib | 20.5 | 11.0 | 31.5 | 0.22 ± 0.37 | 0.12 ± 0.19 | 8.91 | -1.20 |

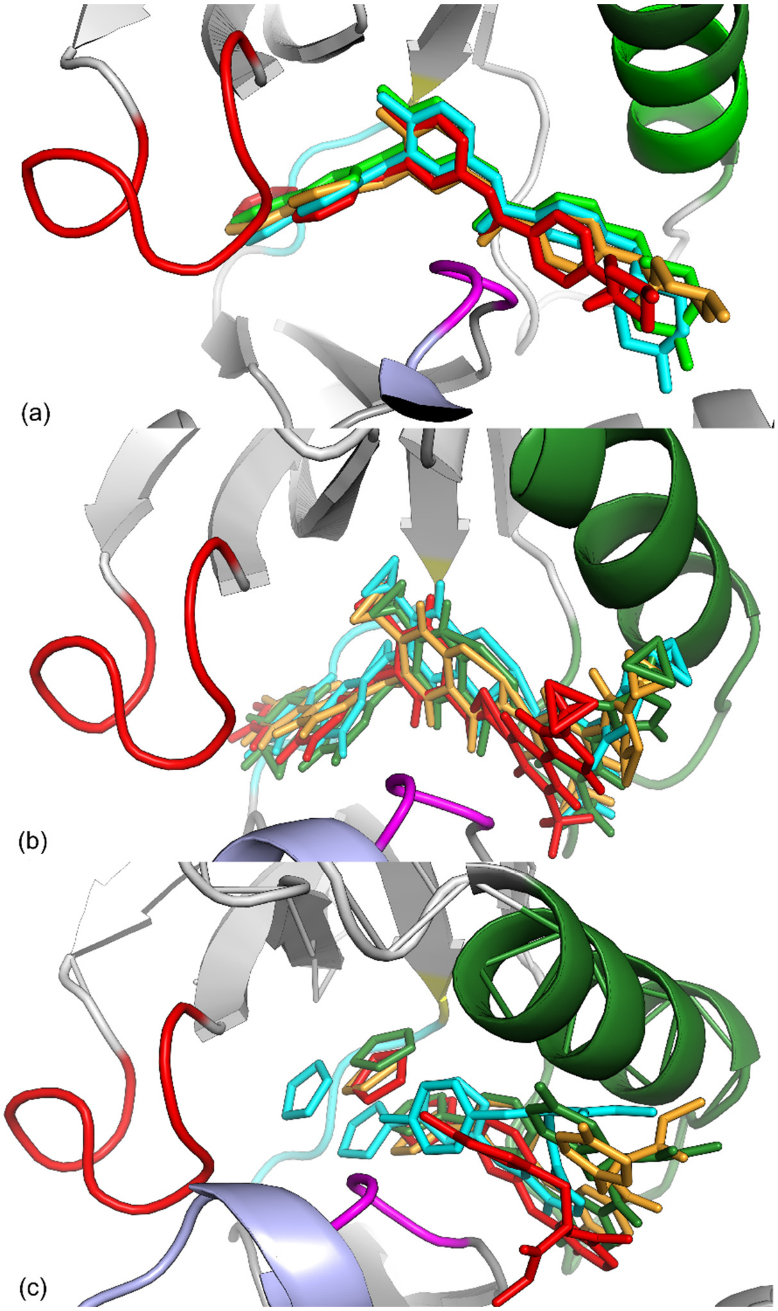


**Figure S13.** Superposition of the initial binding pose (frame 0 of the molecular dynamics simulation, red) and the medoid structures of the most populated ligand clusters identified from the three independent replicas — replica 1 (blue), replica 2 (green), and replica 3 (bright orange) — for (a) imatinib, (b) nilotinib, and (c) compound **15e.** (a) For imatinib, the dominant clusters accounted for 35.7% (178.3 ns, medoid at 160.7 ns), 80.7% (403.5 ns, medoid at 235.8 ns), and 27.9% (139.7 ns, medoid at 125.1 ns) of the respective trajectories. The superposition illustrates the dominant binding geometries sampled during the simulations relative to the starting pose. (b) For nilotinib, the dominant clusters accounted for 50.0% (250.2 ns, medoid at 429.7 ns), 34.4% (172.1 ns, medoid at 464.5 ns), and 49.4% (247.0 ns, medoid at 423.3 ns) of

the respective trajectories; in replica 3, a second, near-equally populated cluster (44.6%) corresponded to a closely related sub-state of the same binding mode. (c) For compound **15e**, the most populated clusters accounted for 26.6% (133.1 ns, medoid at 257.2 ns) and 37.2% (185.9 ns, medoid at 34.8 ns) in replicas 1 and 2, respectively, while replica **3** showed no dominant state, with its three leading clusters nearly equally populated (16.3%, 16.1% and 15.5%); the orange structure in panel (c) therefore represents the largest cluster only. The superpositions illustrate the dominant binding geometries sampled during the simulations relative to the starting pose, with the markedly more dispersed ensemble of compound **15e** reflecting the absence of a persistent, well-defined binding mode.

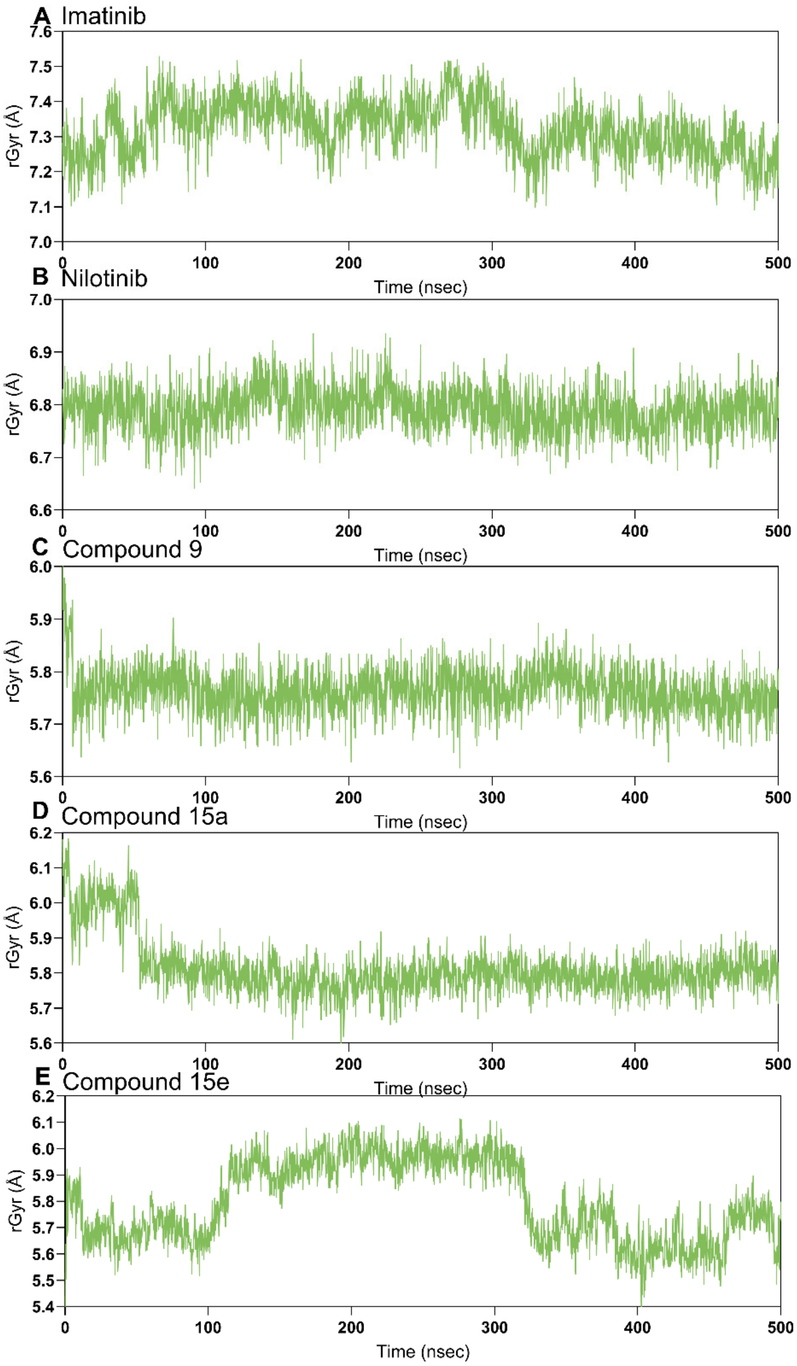


**Figure S14**. Radius of gyration (rGyr) of the ligand over 500 ns molecular dynamics simulations for (A) imatinib, (B) nilotinib, (C) compound **9**, (D) compound **15a**, and **(**E) compound **15e**. The rGyr values are shown as a function of simulation time to monitor the overall compactness and conformational stability of the ligand throughout the trajectory.

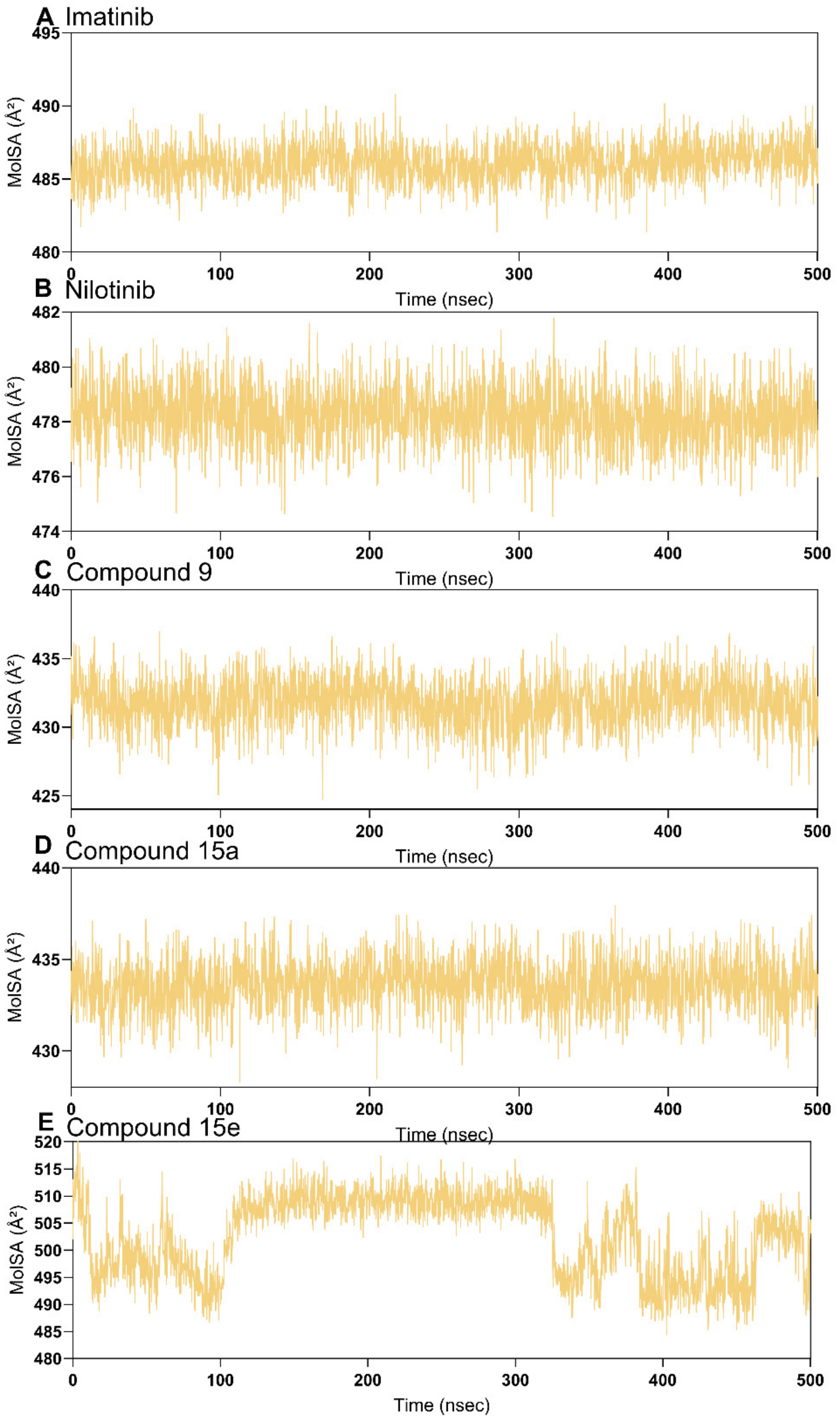


**Figure S15.** Molecular surface area (MolSA) of the ligand over 500 ns molecular dynamics simulations for (A) imatinib, (B) nilotinib, (C) compound **9**, (D) compound **15a**, and **(**E) compound **15e**. MolSA values were calculated for the ligand and plotted as a function of time to monitor changes in molecular surface throughout the simulations.

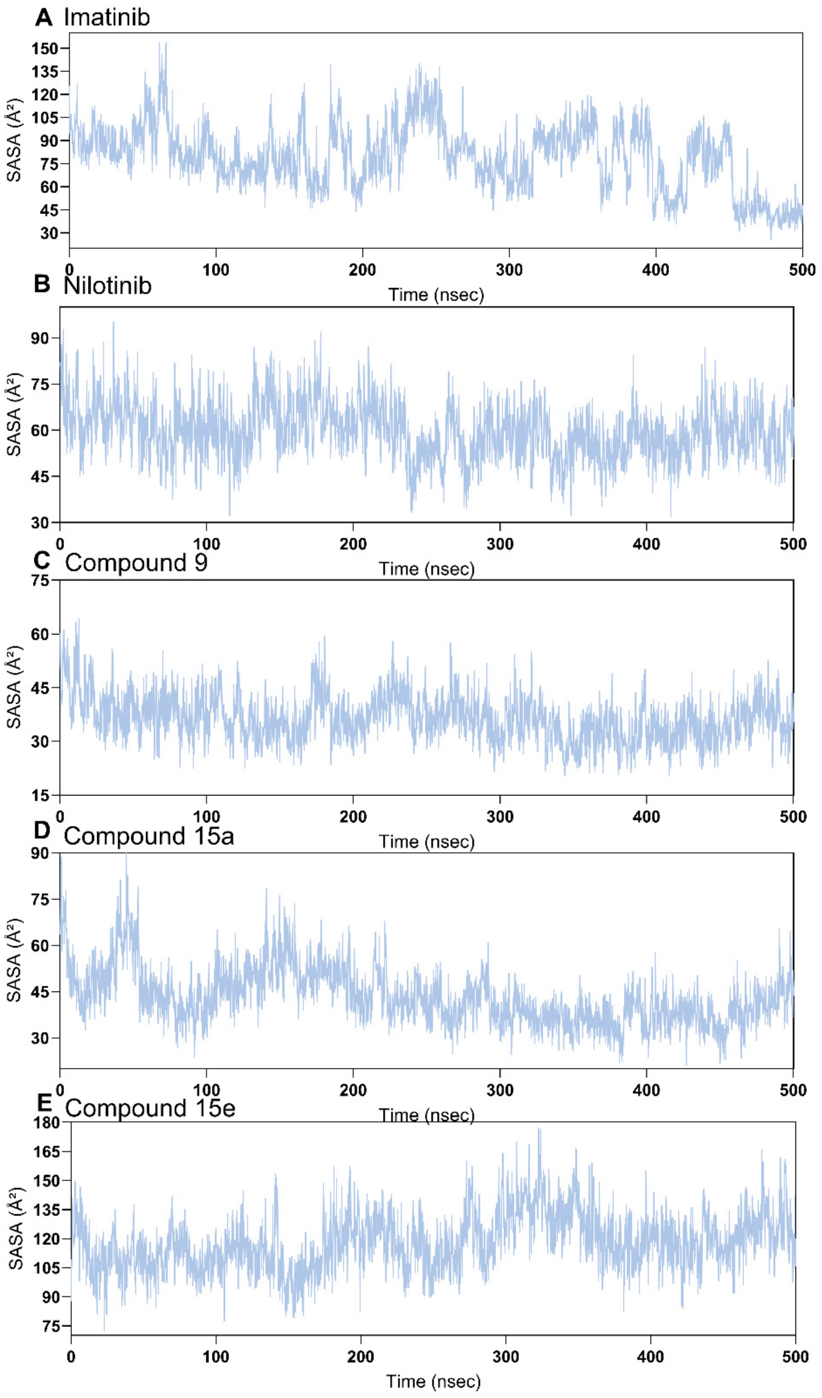


**Figure S16.** Solvent-accessible surface area (SASA) of the ligand over 500 ns molecular dynamics simulations for (A) imatinib, (B) nilotinib, (C) compound **9**, (D) compound **15a**, and **(**E) compound **15e**. SASA values were calculated for the ligand and plotted as a function of time to assess the degree of ligand exposure to the solvent during the simulations.

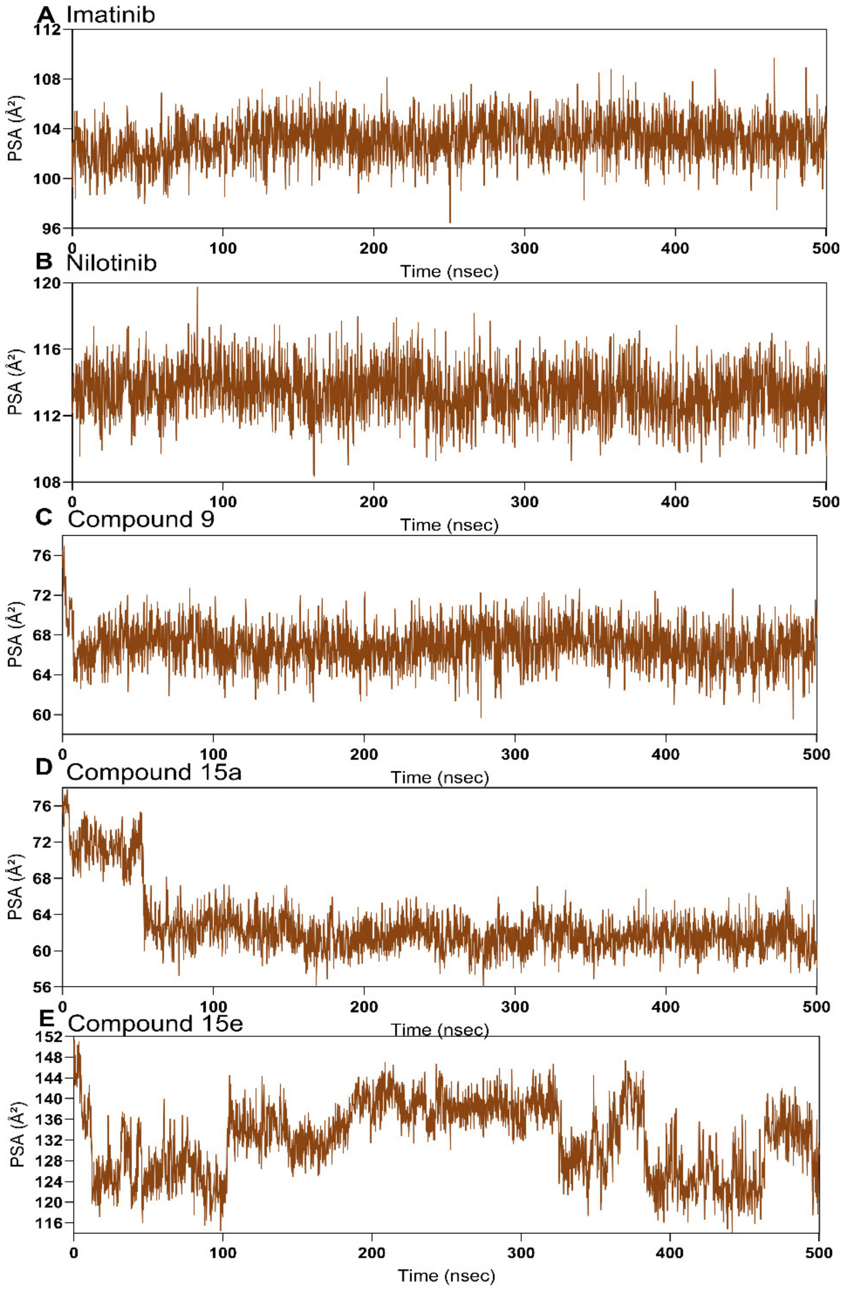


**Figure S17.** Polar surface area (PSA) of the ligand over 500 ns molecular dynamics simulations for (A) imatinib, (B) nilotinib, (C) compound **9**, (D) compound **15a**, and **(**E) compound **15e**. PSA values were calculated for the ligand and plotted over time to monitor changes in the exposed polar surface area during the simulations.

**Table S7**. Quantitative interaction fingerprint analysis derived from molecular dynamics trajectories of imatinib and the Fc-containing analogs. The table summarizes interaction frequencies across three independent MD replicas for each ligand, including hydrogen bonds, hydrophobic contacts, ionic interactions, aromatic interactions, and water bridges. Percentage values represent the proportion of analyzed trajectory frames in which a given interaction is present, calculated over 2500 frames per replica, and thus reflect the occupancy of each interaction over the sampled simulation time.

| Residue | Imatinib | | | | Compound 9 | | | | Compound 15a | | | |
|---|---|---|---|---|---|---|---|---|---|---|---|---|
| | Atom contacts | Rep 1 (%) | Rep 2 (%) | Rep 3 (%) | Atom contacts | Rep 1 (%) | Rep 2 (%) | Rep 3 (%) | Atom contacts | Rep 1 (%) | Rep 2 (%) | Rep 3 (%) |
| **H-bond Interactions** | | | | | | | | | | | | |
| **LYS271** | | 0.0 | 0.0 | 0.0 | pyrimidine N(lig) – HZ1(Lys271); pyrimidine N(lig) – HZ2(Lys271) | 0.0 | 0.1 | 0.0 | pyrimidine N(lig) – HZ1(Lys271); pyrimidine N(lig) – HZ2(Lys271); pyrimidine N(lig) – HZ3(Lys271) | 3.9 | 4.7 | 0.2 |
| **GLU286** | benzamide / piperazine NH(lig) – OE1/OE2(Glu286) | 49.4 | 34.5 | 60.9 | amide NH(lig) – OE1/OE2(Glu286) | 55.1 | 40.4 | 39.1 | amide NH(lig) – O/OE1/OE2(Glu286) | 12.8 | 27.2 | 38.2 |
| **THR315** | aniline NH(lig) – OG1(Thr315) | 55.2 | 55.8 | 32.1 | aniline-type NH(lig) – OG1(Thr315) | 0.2 | 0.0 | 0.5 | aniline-type NH(lig) – OG1(Thr315) | 4.6 | 3.9 | 0.0 |
| **MET318** | pyridine N(lig) – HN(Met318) | 98.3 | 98.6 | 98.2 | | 0.0 | 0.0 | 0.0 | | 0.0 | 0.0 | 0.0 |
| **ASP381** | benzamide carbonyl O(lig) – HN(Asp381) ; protonated piperazine NH(lig) – OD1/OD2(Asp381) | 90.2 | 95.4 | 75.3 | carbonyl O(lig) – HN(Asp381) | 96.3 | 96.0 | 97.2 | amide carbonyl O(lig) – HN(Asp381) | 98.4 | 97.7 | 99.6 |
| **Hydrophobic interactions** | | | | | | | | | | | | |
| **LEU248** | | 48.4 | 46.2 | 41.7 | | 70.4 | 62.6 | 62.1 | | 37.8 | 50.7 | 63.0 |
| **TYR253** | | 5.0 | 4.8 | 6.7 | | 13.9 | 14.0 | 15.4 | | 45.7 | 34.4 | 28.2 |
| **VAL256** | | 13.4 | 16.6 | 16.8 | | 13.1 | 19.8 | 13.1 | | 10.5 | 11.9 | 12.0 |
| **ALA269** | | 30.3 | 26.0 | 35.6 | | 10.8 | 10.2 | 7.0 | | 7.3 | 2.7 | 10.4 |
| **VAL289** | | 6.4 | 5.3 | 4.4 | | 12.4 | 12.0 | 9.2 | | 8.2 | 15.0 | 13.6 |
| **MET290** | | 43.2 | 38.8 | 41.3 | | 43.8 | 46.4 | 39.2 | | 35.1 | 39.8 | 37.0 |
| **ILE293** | | 14.6 | 29.6 | 7.5 | | 19.8 | 15.2 | 18.4 | | 15.8 | 22.7 | 20.3 |
| **LEU298** | | 0.4 | 0.4 | 0.7 | | 0.4 | 0.4 | 0.7 | | 1.8 | 1.2 | 0.4 |
| **VAL299** | | 9.4 | 8.2 | 13.8 | | 6.6 | 9.5 | 10.6 | | 13.4 | 6.7 | 12.0 |

| | | | | | | | | | | | |
|---|---|---|---|---|---|---|---|---|---|---|---|
| **LEU301** | | 0.1 | 0.0 | 0.0 | | 0.0 | 0.0 | 0.0 | | 0.0 | 0.0 | 0.0 |
| **ILE313** | | 34.2 | 36.0 | 31.1 | | 5.4 | 4.2 | 4.9 | | 5.2 | 1.3 | 1.1 |
| **PHE317** | | 36.3 | 40.3 | 39.7 | | 13.9 | 16.7 | 11.7 | | 6.8 | 4.2 | 15.3 |
| **MET318** | | 0.1 | 0.0 | 0.1 | | 0.8 | 1.0 | 1.0 | | 16.7 | 6.3 | 0.5 |
| **LEU354** | | 7.9 | 6.4 | 8.4 | | 0.0 | 0.0 | 0.0 | | 0.3 | 0.0 | 0.0 |
| **PHE359** | | 6.6 | 5.3 | 6.1 | | 3.8 | 7.3 | 4.8 | | 40.8 | 2.0 | 4.8 |
| **LEU370** | | 27.0 | 28.0 | 31.1 | | 81.4 | 65.9 | 69.6 | | 34.3 | 38.4 | 72.1 |
| **PHE382** | | 66.2 | 66.6 | 68.5 | | 19.6 | 29.2 | 27.5 | | 31.6 | 49.4 | 41.0 |
| **Ionic interactions** | | | | | | | | | | | | |
| **ASP381** | protonated piperazine $N^{+}$(lig) – OD2(Asp381) | 16.8 | 17.6 | 14.8 | | 0.0 | 0.0 | 0.0 | | 0.0 | 0.0 | 0.0 |
| **Pi-Cation Interactions** | | | | | | | | | | | | |
| **LYS271** | | 9.8 | 11.3 | 10.4 | | 80.4 | 70.9 | 78.8 | | 81.9 | 87.0 | 75.7 |
| **PHE359** | | 31.6 | 30.8 | 41.1 | | 0.0 | 0.0 | 0.0 | | 0.0 | 0.0 | 0.0 |
| **HIS361** | | 51.8 | 42.2 | 19.9 | | 0.0 | 0.0 | 0.0 | | 0.0 | 0.0 | 0.0 |
| **ARG386** | | 3.1 | 1.2 | 2.7 | | 1.1 | 1.1 | 2.0 | | 0.1 | 0.5 | 1.1 |
| **Pi-Pi Interactions** | | | | | | | | | | | | |
| **TYR253** | e2f(lig) – π-stacking(prot) | 68.7 | 67.6 | 68.5 | e2f(lig) – π-stacking(prot) | 31.4 | 28.9 | 27.8 | e2f(lig) – π-stacking(prot); f2f(lig) – π-stacking(prot) | 8.6 | 7.9 | 20.0 |
| **PHE317** | e2f(lig) – π-stacking(prot); f2f(lig) – π-stacking(prot) | 6.0 | 8.2 | 7.9 | | 0.0 | 0.0 | 0.0 | | 0.0 | 0.0 | 0.0 |
| **HIS361** | e2f(lig) – π-stacking(prot) | 0.1 | 0.1 | 0.0 | e2f(lig) – π-stacking(prot) | 0.4 | 0.4 | 0.4 | e2f(lig) – π-stacking(prot); f2f(lig) – π-stacking(prot) | 0.4 | 2.1 | 0.9 |
| **PHE382** | e2f(lig) – π-stacking(prot); f2f(lig) – π-stacking(prot) | 12.6 | 12.8 | 13.4 | e2f(lig) – π-stacking(prot); f2f(lig) – π-stacking(prot) | 62.2 | 59.3 | 54.6 | e2f(lig) – π-stacking(prot); f2f(lig) – π-stacking(prot) | 36.4 | 32.1 | 41.6 |
| **Water Bridge Interactions** | | | | | | | | | | | | |
| **TYR253** | pyrimidine N(lig) – O(Tyr253) | 0.7 | 0.9 | 0.2 | pyrimidine N(lig) – O(Tyr253) | 0.1 | 0.3 | 0.0 | pyrimidine N(lig) – O(Tyr253) | 4.3 | 0.3 | 0.1 |
| **LYS271** | pyrimidine N / carbonyl O(lig) – HZ1/HZ2/HZ3(Lys271) | 5.8 | 7.0 | 5.9 | pyrimidine N / carbonyl O / amide NH(lig) – HZ1/HZ2/HZ3(Lys271) | 89.3 | 78.3 | 80.9 | pyrimidine N / carbonyl O / amide NH(lig) – HZ1/HZ2/HZ3(Lys271) | 64.0 | 80.8 | 75.2 |

| | | | | | | | | | | | | |
|---|---|---|---|---|---|---|---|---|---|---|---|---|
| **GLU286** | amide NH / piperazine NH / pyrimidine N / carbonyl O(lig) – O/OE1/OE2(Glu286) | 2.8 | 3.6 | 4.0 | amide NH / pyrimidine N / carbonyl O(lig) – O/OE1/OE2(Glu286) | 1.1 | 2.0 | 2.6 | amide NH / pyrimidine N / carbonyl O(lig) – O/OE1/OE2(Glu286) | 11.6 | 20.8 | 9.2 |
| **VAL299** | pyrimidine N / carbonyl O(lig) – H/O(Val299) | 4.7 | 2.2 | 12.3 | carbonyl O(lig) – H/O(Val299) | 1.3 | 2.2 | 0.7 | carbonyl O(lig) – H/O(Val299) | 2.3 | 1.4 | 0.7 |
| **THR315** | pyrimidine N / aniline NH(lig) – HG1/OG1(Thr315) | 42.6 | 43.7 | 46.7 | pyrimidine N / aniline-type NH(lig) – HG1/OG1(Thr315) | 5.5 | 6.9 | 7.2 | pyrimidine N / aniline-type NH / amide NH(lig) – HG1/OG1(Thr315) | 5.0 | 2.4 | 4.0 |
| **GLU316** | aniline NH / pyrimidine N(lig) – O(Glu316) | 14.8 | 19.0 | 20.4 | aniline-type NH / pyrimidine N(lig) – O(Glu316) | 8.6 | 13.6 | 7.3 | aniline-type NH / pyrimidine N(lig) – O(Glu316) | 9.3 | 2.4 | 13.9 |
| **ILE360** | piperazine NH / pyrimidine N(lig) – H/O(Ile360) | 5.5 | 2.8 | 3.2 | | 0.0 | 0.0 | 0.0 | | 0.0 | 0.0 | 0.0 |
| **HIS361** | piperazine NH / pyrimidine N(lig) – ND1/O(His361) | 10.5 | 4.4 | 9.0 | | 0.0 | 0.0 | 0.0 | | 0.0 | 0.0 | 0.0 |
| **ARG362** | pyrimidine N(lig) – HE/HH11/HH12/HH21/HH22(Arg362) | 0.1 | 1.0 | 1.8 | | 0.0 | 0.0 | 0.0 | | 0.0 | 0.0 | 0.0 |
| **VAL379** | carbonyl O(lig) – O(Val379) | 2.2 | 0.4 | 5.7 | carbonyl O(lig) – O(Val379) | 1.7 | 1.6 | 0.8 | carbonyl O(lig) – O(Val379) | 2.7 | 1.3 | 0.5 |
| **ASP381** | carbonyl O / amide NH / pyrimidine N / piperazine NH(lig) – O/OD1/OD2 (Asp381) | 25.4 | 22.4 | 23.2 | amide NH / pyrimidine N / carbonyl O(lig) – O/OD1/OD2(Asp381) | 88.6 | 60.2 | 80.1 | amide NH / pyrimidine N / carbonyl O(lig) – O/OD1/OD2(Asp381) | 75.2 | 80.6 | 77.0 |
| **PHE382** | pyrimidine N(lig) – O(Phe382) | 0.0 | 0.0 | 0.0 | pyrimidine N(lig) – O(Phe382) | 0.7 | 1.4 | 0.9 | pyrimidine N(lig) – O(Phe382) | 0.2 | 3.6 | 0.8 |

**Table S8.** Quantitative interaction fingerprint analysis derived from molecular dynamics trajectories of nilotinib and compound **15e**. The table summarizes interaction frequencies across three independent MD replicas for each ligand, including hydrogen bonds, hydrophobic contacts, ionic interactions, aromatic interactions, and water bridges. Percentage values represent the proportion of analyzed trajectory frames in which a given interaction is presen, calculated over 2500 frames per replica, and thus reflect the occupancy of each interaction over the sampled simulation time.

| Residue | Nilotinib | | | | Compound 15e | | | |
|---|---|---|---|---|---|---|---|---|
| | Atom contacts | Rep 1 (%) | Rep 2 (%) | Rep 3 (%) | Atom contacts | Rep 1 (%) | Rep 2 (%) | Rep 3 (%) |
| | | | | **Hydrogen-bond interactions** | | | | |
| **LYS271** | — | 0 | 0 | 0 | — | 0.4 | 0.8 | 0 |
| **GLU286** | benzamide amide N–H(lig) – OE1/OE2(Glu286) | 50.8 | 68.7 | 50.6 | amide N–H(lig) – OE1/OE2(Glu286) | 0.8 | 3.9 | 0.7 |
| **THR315** | anilino-pyrimidine N–H(lig) – OG1(Thr315) | 78.8 | 81 | 81.1 | — | 0.4 | 0 | 0 |
| **MET318** | pyridine N(lig) – HN(Met318) | 99.2 | 98.5 | 99.2 | — | 0 | 0 | 0 |
| **ASP381** | benzamide carbonyl O(lig) – HN(Asp381) | 98.8 | 98.3 | 98 | amide N(lig) – HN(Asp381); phenolic O–H(lig) – OD1/OD2/O(Asp381) | 98.8 | 89.5 | 90.7 |
| **ARG362** | — | 0.5 | 0.1 | 1.4 | O4328(lig) – HH22(Arg362) | 9.2 | 7.6 | 2.8 |
| **ARG386** | — | 0 | 0 | 0 | O4328(lig) – HH12(Arg386) | 0.8 | 0.2 | 2.8 |
| **GLU282** | — | 0 | 0 | 0 | phenolic O–H(lig) – OE1/OE2(Glu282) | 0 | 6.5 | 44.7 |
| **LYS285** | — | 0 | 0 | 0.2 | phenolic O(lig) – HZ1/HZ2/HZ3(Lys285) | 0 | 1.3 | 4.6 |
| | | | | **Hydrophobic interactions** | | | | |
| **LEU248** | | 47.4 | 53.3 | 54.6 | | 11.2 | 4.8 | 0 |
| **TYR253** | | 8 | 13.1 | 6.9 | | 0.1 | 0 | 0 |
| **VAL256** | | 10.3 | 4.6 | 7.6 | | 9.8 | 8.4 | 9.7 |
| **ALA269** | | 39.2 | 21.5 | 28.1 | | 38.6 | 13.3 | 0.9 |
| **VAL289** | | 34.9 | 30.7 | 30.9 | | 12.2 | 19 | 17.7 |
| **MET290** | | 51.4 | 20.6 | 39.5 | | 24.4 | 40.6 | 45.3 |
| **ILE293** | | 2.2 | 5.3 | 2.9 | | 15.2 | 13.1 | 8 |
| **LEU298** | | 4.2 | 2.3 | 4.4 | | 17.3 | 23.3 | 23.3 |
| **VAL299** | | 15.6 | 5.6 | 10.5 | | 17.8 | 20.2 | 11.1 |
| **LEU301** | | 0 | 0 | 0 | | 2.1 | 0 | 0 |
| **ILE313** | | 14.4 | 21.7 | 26.7 | | 0.2 | 2.8 | 4.2 |
| **PHE317** | | 41.2 | 31.4 | 39.5 | | 12 | 0.7 | 0 |
| **MET318** | | 0.2 | 1.6 | 0.2 | | 0 | 0 | 0 |
| **LEU354** | | 0 | 0 | 0 | | 0.3 | 2.5 | 3.2 |
| **PHE359** | | 3.4 | 18.9 | 5.6 | | 19.4 | 16.6 | 6.7 |
| **LEU370** | | 26.8 | 35.3 | 29.1 | | 1.3 | 0.5 | 0.7 |
| **PHE382** | | 69.1 | 32.3 | 75.8 | | 78.7 | 65.3 | 64.9 |

| | | | | | | | | |
|---|---|---|---|---|---|---|---|---|
| **VAL379** | | 0 | 0 | 0 | | 0.7 | 1.2 | 1.2 |
| **ALA380** | | 0 | 0.1 | 0 | | 15.2 | 20.4 | 26.9 |
| **Ionic interactions** | | | | | | | | |
| **ASP381** | protonated N+(lig) – OD2(Asp381) | 0 | 0 | 0 | — | 0 | 0 | 0 |
| **π–Cation interactions** | | | | | | | | |
| **LYS271** | | 35 | 35.7 | 19.1 | | 2 | 6.9 | 0.2 |
| **PHE359** | | 0 | 0 | 0 | | 0 | 0 | 0 |
| **HIS361** | | 0 | 0 | 0 | | 0 | 0 | 0 |
| **ARG386** | | 50.4 | 6.8 | 24.9 | | 33.8 | 15.7 | 14.4 |
| **ARG362** | | 0.6 | 0.2 | 1.8 | | 0.2 | 5.9 | 0.8 |
| **LYS285** | | 0 | 0 | 0 | | 0 | 9.9 | 7.8 |
| **π–π interactions** | | | | | | | | |
| **TYR253** | e2f(lig) – π-stacking(prot) | 42.2 | 63 | 59.7 | — | 0 | 0 | 0 |
| **PHE317** | e2f / f2f(lig) – π-stacking(prot) | 6.8 | 8 | 6.7 | — | 0 | 0 | 0 |
| **PHE359** | e2f(lig) – π-stacking(prot) | 8.8 | 10.7 | 8.6 | e2f(lig) – π-stacking(prot) | 0.1 | 2 | 2.8 |
| **PHE382** | e2f / f2f(lig) – π-stacking(prot) | 3.9 | 4.6 | 6.3 | — | 0 | 0 | 0 |
| **HIS361** | — | 0 | 0 | 0 | e2f / f2f(lig) – π-stacking(prot) | 10.6 | 21 | 18.7 |
| **Water-bridge interactions** | | | | | | | | |
| **TYR253** | | 7.5 | 0.2 | 1.2 | | 0.1 | 0 | 0 |
| **LYS271** | | 19 | 15.9 | 7.3 | | 37.4 | 7.3 | 3.4 |
| **GLU286** | | 1.5 | 0.6 | 4.3 | | 17.3 | 12.5 | 23.3 |
| **VAL299** | | 0 | 0.6 | 0.2 | | 6.7 | 7.8 | 9.5 |
| **THR315** | | 26.1 | 6.5 | 21.2 | | 0.4 | 0 | 0 |
| **GLU316** | | 17.6 | 5.9 | 12.9 | | 0 | 0 | 0 |
| **ILE360** | | 0.1 | 0.3 | 0.1 | | 14.4 | 51.7 | 78.4 |
| **HIS361** | | 0 | 0.2 | 0 | | 2.2 | 5 | 6.9 |
| **ARG362** | | 7 | 1.1 | 10 | | 23.3 | 14.7 | 7.5 |
| **VAL379** | | 0 | 0.7 | 0.4 | | 0 | 2.8 | 1.5 |
| **ASP381** | | 5 | 24.8 | 10.9 | | 23.7 | 42 | 43.6 |
| **PHE382** | | 0.1 | 0 | 0 | | 21.9 | 2.2 | 1.4 |
| **ARG386** | | 0.8 | 0.3 | 0.9 | | 10.7 | 6.7 | 11 |
| **GLU282** | | 1.4 | 6.2 | 3.1 | | 0 | 9.1 | 12 |
| **LYS285** | | 2.1 | 2 | 3.7 | | 1.3 | 12.3 | 13.3 |

**Table S9**. MM-GBSA energy decomposition analysis for imatinib, nilotinib, compound **9**, **15a**, and **15e** based on the last 100 ns of the molecular dynamics simulations (400–500 ns). Values are presented as mean ± SD across trajectory frames for each independent replica, together with the overall mean ± SD across the three replicas. The table reports the total binding free energy estimate ($\Delta G_{bind}$) and its individual components, including the Coulombic component, covalent, van der Waals. Lipophilic, solvation (GB), hydrogen-bonding, and packing terms, all expressed in kcal/mol.

| MM-GBSA Energy Decomposition – molecular dynamics (400–500 ns. mean ± SD. kcal/mol) | | | | | | | | |
|---|---|---|---|---|---|---|---|---|
| Imatinib | | | | | | | | |
| Replica | $\Delta G_{bind}$ | $\Delta G_{Coulomb}$ | $\Delta G_{Covalent}$ | $\Delta G_{vdW}$ | $\Delta G_{Lipo}$ | $\Delta G_{Solv}$ | $\Delta G_{Hbond}$ | $\Delta G_{Packing}$ |
| | Mean ± SD | Mean ± SD | Mean ± SD | Mean ± SD | Mean ± SD | Mean ± SD | Mean ± SD | Mean ± SD |
| Replica 1 | -115.56 ± 3.82 | -84.06 ± 13.34 | 4.33 ± 1.09 | -83.03 ± 2.10 | -39.65 ± 1.48 | 91.75 ± 13.32 | -2.22 ± 0.29 | -2.68 ± 0.65 |
| Replica 2 | -111.48 ± 4.75 | -82.96 ± 12.21 | 4.13 ± 1.09 | -81.62 ± 2.56 | -39.20 ± 1.63 | 93.02 ± 11.94 | -1.95 ± 0.34 | -2.91 ± 0.46 |
| Replica 3 | -102.42 ± 9.94 | -75.93 ± 14.07 | 4.11 ± 1.49 | -76.13 ± 5.65 | -36.46 ± 3.28 | 86.59 ± 13.10 | -1.81 ± 0.40 | -2.80 ± 0.64 |
| Overall | -109.82 ± 8.68 | -80.98 ± 13.67 | 4.19 ± 1.24 | -80.26 ± 4.81 | -38.44 ± 2.68 | 90.45 ± 13.06 | -1.99 ± 0.39 | -2.80 ± 0.59 |
| Nilotinib | | | | | | | | |
| Replica 1 | −106.80 ± 4.01 | −24.38 ± 2.27 | 1.93 ± 1.11 | −81.17 ± 2.22 | −33.96 ± 1.33 | 36.40 ± 2.31 | −1.97 ± 0.17 | −3.65 ± 0.57 |
| Replica 2 | −101.00 ± 4.59 | −21.13 ± 2.19 | 0.42 ± 1.24 | −75.68 ± 2.66 | −33.56 ± 1.44 | 33.17 ± 2.00 | −1.85 ± 0.20 | −2.37 ± 0.56 |
| Replica 3 | −101.85 ± 4.56 | −21.73 ± 3.04 | 3.11 ± 1.06 | −78.65 ± 2.39 | −33.96 ± 1.38 | 33.87 ± 2.53 | −1.76 ± 0.24 | −2.73 ± 0.66 |
| Overall | −103.22 ± 3.13 | −22.41 ± 1.73 | 1.82 ± 1.35 | −78.50 ± 2.75 | −33.83 ± 0.23 | 34.48 ± 1.70 | −1.86 ± 0.10 | −2.92 ± 0.66 |
| Compound **9** | | | | | | | | |
| Replica 1 | -40.38 ± 4.49 | -8.63 ± 5.71 | 4.09 ± 1.17 | -66.95 ± 2.23 | -39.83 ± 1.48 | 73.83 ± 7.74 | -0.77 ± 0.18 | -2.12 ± 0.33 |
| Replica 2 | -39.20 ± 4.48 | -10.20 ± 5.66 | 3.68 ± 1.11 | -68.50 ± 2.73 | -39.23 ± 2.27 | 77.63 ± 7.56 | -0.69 ± 0.16 | -1.90 ± 0.33 |
| Replica 3 | -37.33 ± 4.30 | -7.47 ± 5.83 | 3.51 ± 0.98 | -66.82 ± 2.40 | -39.19 ± 2.00 | 75.19 ± 7.26 | -0.56 ± 0.14 | -2.00 ± 0.41 |
| Overall | -38.97 ± 4.59 | -8.77 ± 5.82 | 3.76 ± 1.11 | -67.42 ± 2.57 | -39.41 ± 1.96 | 75.55 ± 7.66 | -0.67 ± 0.18 | -2.01 ± 0.37 |
| Compound **15a** | | | | | | | | |
| Replica 1 | -45.44 ± 3.52 | -11.49 ± 5.38 | 1.10 ± 0.62 | -65.01 ± 2.31 | -40.18 ± 1.99 | 72.84 ± 7.40 | -0.57 ± 0.10 | -2.13 ± 0.55 |
| Replica 2 | -39.67 ± 4.24 | -12.10 ± 5.11 | 2.55 ± 1.36 | -65.11 ± 2.44 | -37.84 ± 1.93 | 75.49 ± 6.67 | -0.84 ± 0.29 | -1.82 ± 0.52 |
| Replica 3 | -38.51 ± 4.21 | -8.14 ± 5.83 | 2.60 ± 0.94 | -66.66 ± 2.60 | -39.41 ± 1.83 | 75.73 ± 7.13 | -0.63 ± 0.24 | -1.98 ± 0.39 |
| Overall | -41.21 ± 5.02 | -10.58 ± 5.70 | 2.08 ± 1.23 | -65.59 ± 2.56 | -39.14 ± 2.15 | 74.69 ± 7.17 | -0.68 ± 0.25 | -1.98 ± 0.50 |
| Compound **15e** | | | | | | | | |
| Replica 1 | −24.35 ± 5.43 | −14.10 ± 5.88 | 1.20 ± 0.97 | −63.47 ± 3.17 | −29.01 ± 1.53 | 83.28 ± 6.64 | −1.04 ± 0.20 | −1.22 ± 0.58 |
| Replica 2 | −19.08 ± 5.77 | −9.84 ± 7.45 | 1.46 ± 1.35 | −62.90 ± 3.13 | −31.29 ± 2.03 | 84.73 ± 7.26 | −0.75 ± 0.38 | −0.49 ± 0.43 |
| Replica 3 | −23.37 ± 6.03 | −10.39 ± 6.54 | 3.06 ± 1.66 | −71.27 ± 3.08 | −32.18 ± 1.84 | 91.18 ± 7.34 | −1.17 ± 0.52 | −2.61 ± 0.67 |
| Overall | −22.27 ± 2.80 | −11.44 ± 2.32 | 1.91 ± 1.00 | −65.88 ± 4.67 | −30.82 ± 1.63 | 86.40 ± 4.20 | −0.98 ± 0.21 | −1.44 ± 1.08 |
